# Homeostatic controllers compensating for growth and perturbations

**DOI:** 10.1371/journal.pone.0207831

**Published:** 2019-08-12

**Authors:** Peter Ruoff, Oleg Agafonov, Daniel M. Tveit, Kristian Thorsen, Tormod Drengstig

**Affiliations:** 1 Centre for Organelle Research, University of Stavanger, Stavanger, Norway; 2 Department of Electrical Engineering and Computer Science, University of Stavanger, Stavanger, Norway; INRA, FRANCE

## Abstract

Cells and organisms have developed homeostatic mechanisms which protect them against a changing environment. How growth and homeostasis interact is still not well understood, but of increasing interest to the molecular and synthetic biology community to recognize and design control circuits which can oppose the diluting effects of cell growth. In this paper we describe the performance of selected negative feedback controllers in response to different applied growth laws and time dependent outflow perturbations of a controlled variable. The approach taken here is based on deterministic mass action kinetics assuming that cell content is instantaneously mixed. All controllers behave ideal in the sense that they for step-wise perturbations in volume and a controlled compound *A* are able to drive *A* precisely back to the controllers’ theoretical set-points. The applied growth kinetics reflect experimentally observed growth laws, which range from surface to volume ratio growth to linear and exponential growth. Our results show that the kinetic implementation of integral control and the structure of the negative feedback loop are two properties which affect controller performance. Best performance is observed for controllers based on derepression kinetics and controllers with an autocatalytic implementation of integral control. Both are able to defend exponential growth and perturbations, although the autocatalytic controller shows an offset from its theoretical set-point. Controllers with activating signaling using zero-order or bimolecular (antithetic) kinetics for integral control behave very similar but less well. Their performance can be improved by implementing negative feedback structures having repression/derepression steps or by increasing controller aggressiveness. Our results provide a guide what type of feedback structures and integral control kinetics are suitable to oppose the dilution effects by different growth laws and time dependent perturbations on a deterministic level.

## Introduction

The term *homeostasis* was defined by Walter B. Cannon [1] to describe the coordinated ability of organisms and cells to maintain an internal stability by keeping concentrations of cellular components within certain tolerable limits [2]. Cannon’s emphasis on *homeo* indicates that he considered the internal physiological state not as a constant, as suggested earlier by Bernard’s concept of a fixed “milieu intérieur” [2, 3], but conceives homeostasis as a dynamic adaptable system which allows variations within certain limits. Dependent on the controlled components, the homeostatic limits in which one or several controllers operate can vary considerably. For example, while the negative feedback regulation of cellular sodium shows an apparently changing and less well-defined set-point [4, 5], the regulation of other metal ions have more strict limits [6–8].

Growth, an essential aspect of all living beings is a highly regulated process. According to Bertalanffy [9, 10], the different observed growth kinetics of organisms can be related to the organisms’ metabolism. For example, when respiration is proportional to the surface of the organism linear growth kinetics are obtained. On the other hand, if respiration is proportional to the organism’s weight/volume, exponential growth occurs. Growth kinetics of bacteria [11, 12] appear closely related to the bacterial form or shape. Rod-shaped bacteria show exponential growth rates, i.e.

$$\begin{array}{c} \dot{V}=\kappa V\;;\;\;\kappa >0 \end{array}$$ (1)

whereas spherical bacteria increase their cellular volume by a rate law related to the surface to volume ratio, i.e.,

$$\begin{array}{c} \dot{V}=\eta \cdot V^{\frac{2}{3}}-\xi \cdot V \end{array}$$ (2)

where *η* and *ξ* are constants reflecting anabolism and catabolism, respectively [13].

Although the protective functions of homeostasis need to be in place during growth, the interacting mechanisms between homeostasis and growth are not well understood. In principle, there are two aspects of growth to consider. The first one, which is focused on in this paper is how homeostatic mechanisms can compensate for growth without affecting it. The second aspect, which will be treated in another paper, is how homeostatic mechanisms can influence growth. In this paper we consider growth as an increase of the cellular volume. As a continuous process growth represents a time-dependent perturbation which would lead to the dilution of cellular/cytosolic compounds unless other mechanisms counteract for it.

**Fig 1:**
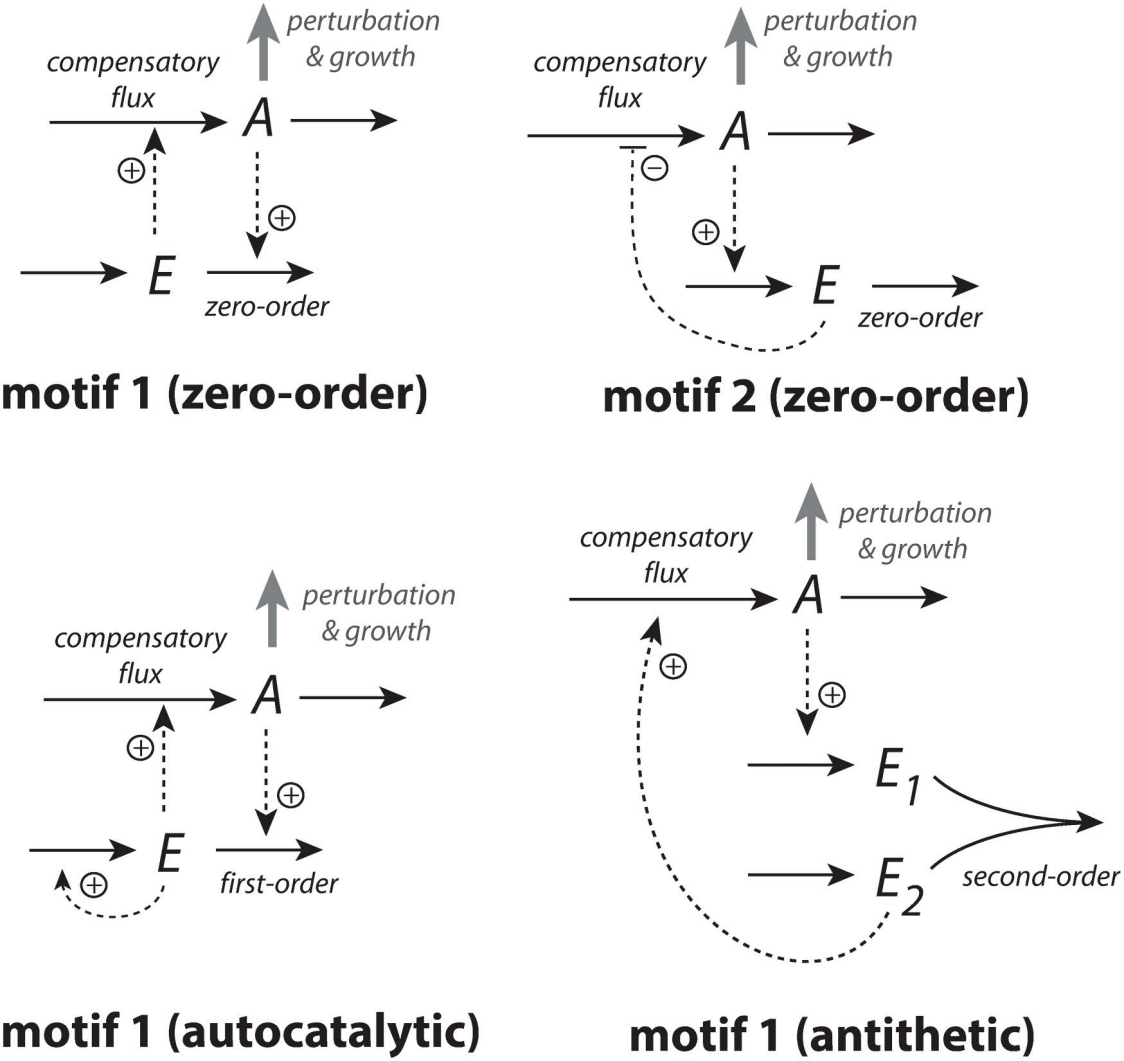
The controllers investigated in this study. Reaction orders are with respect to *E*. The reaction between *E*_1_ and *E*_2_ in the antithetic controller is an overall second-order process. The controllers behave ideal in the sense that they for step-wise changes in *A* and/or *V*, are able to keep *A* precisely at their defined theoretical set-points $A_{set}^{theor}$.

Integral control is a concept from control engineering [14], which enables robust regulation for step-wise perturbations and has been implicated to occur in a variety of homeostatic regulated systems [5, 15–17]. How different integral controllers will perform under (nonlinear) time-dependent growth is little investigated. Based on a previous study [18] we have chosen four controller motifs, which are shown in Fig 1. The most promising controllers which are able to handle nonlinear time dependent growth are a motif 2 zero-order type of controller based on derepression and a motif 1 first-order controller based on autocatalysis [19–21]. A relatively new discovered integral feedback mechanism, the so-called antithetic motif [22], has also been included. For comparison, we have also included a motif 1 zero-order type of controller. The controllers were investigated with respect to their capabilities to compensate for time-dependent outflow perturbations in *A* and in the presence of different growth laws (increase in the reaction volume *V*) according to Bertalanffy’s classifications [9, 10]. The growth kinetics that will be considered include linear (constant) as well as saturating and exponential growth laws. We focus here primarily on outflow perturbations, because together with the diluting effects of the different growth laws these perturbations represent the most severe conditions for testing the controllers.

## Materials and methods

To arrive at controller candidates which can oppose various dilution and perturbation kinetics a couple of simplifications have been made, which are discussed in more detail below. One is the assumption that compounds in a growing cells undergo instantaneous and ideal mixing, thereby ignoring the spatial organization of the cell. In addition, we ignore stochastic effects due to diffusion or low molecule numbers (however, see Discussion). Deterministic computations were performed by using the Fortran subroutine LSODE [23]. Plots were generated with gnuplot (www.gnuplot.info) and Adobe Illustrator (adobe.com). To make notations simpler, concentrations of compounds are denoted by compound names without square brackets. Time derivatives are generally indicated by the ‘dot’ notation. Concentrations and rate parameter values are given in arbitrary units (au). Rate parameters are presented as *k*_*i*_’s (*i* = 1, 2, 3, …) irrespective of their kinetic nature, i.e. whether they represent turnover numbers, Michaelis constants, or inhibition constants. A set of MATLAB (mathworks.com) calculations with instructions are provided in the Supporting Information as a combined zip-file (S1 Matlab).

### Overview of treated cases and analytical steady state expressions

The four controller motifs are studied for internal and transporter-based compensatory fluxes, different growth laws, and different removal kinetics of the controlled variable *A*. In the following we give a brief summary how the paper is structured and under what conditions the four motifs are tested. The paper divides into the following major parts.

In chapter “Reaction kinetics during volume changes” the rate equations during volume changes are derived.

The results are divided into two major cases:

In Case A: “Controllers with transporter-based compensatory fluxes” the behaviors of the four negative feedback motifs are shown when the compensatory fluxes are transporter based and when systems are exposed to linear and exponential growth with corresponding removal kinetics in *A* during growth. The transporter-based compensatory fluxes consist of an (by controller molecule *E* activated or derepressed) zero-order inflow of *A* molecules with respect to the transporter, $\dot{n_{A}}$, which for each time point is divided by the volume to get the contribution to the concentration of *A* due to the inflow.

In Case B: “Controllers with cell-internal compensatory fluxes” results are described when the compensatory fluxes are generated cell-internally and when the systems are exposed to linear, exponential, and surface-to-volume ratio related growth. Also here, during growth, *A* is subject to linear and exponential removal kinetics.

For most of the numerically studied control structures analytical steady state expressions for *A* are derived in the Supporting Information. The analytical expressions in *A*_*ss*_ are derived by writing first down the rate equations for *A* and *E* (*E*_1_ and *E*_2_ for the antithetic controller), while treating fluxes coming from precursor species as constants, i.e., rates are zero-order with respect to these species. Then the second time-derivative *Ä* is calculated and the rate equation of *E* (*E*_2_ for the antithetic controller) is inserted into the *Ä* equation which is set to zero. This leads to an analytical expression for *A*_*ss*_ showing how different parameters influence the steady state.

In “Overview of results” the four motifs are ranked according to their abilities to oppose the different growth laws and outflow perturbations. The motif 2 based controller with repression/derepression kinetics clearly outperforms the other motifs, followed by the autocatalytic motif 1 controller. The performance of the four motifs is discussed in terms of the internal model principle, which reflects the kinetic limits controllers can handle.

We also demonstrate the influence the feedback structure (termed motifs in [7]) has in relationship with the integral controller part. Using an antithetic integral controller together with a motif 2 repression/derepression structure as an example, we show how the motif 2 structure improves controller performance, but also point to the limitations which are caused by the kinetics of the integral controller.

## Reaction kinetics during volume changes

To describe concentration changes during cell growth we have to consider the concentration changes due to the increasing reaction volume *V*. If *A* denotes the concentration of *n*_*A*_ moles of compound *A* in volume *V*, the overall change of concentration *A* is composed of two terms, one that describes the changes of *A* while *V* is kept constant, ${\left(\dot{A}\right)}_{V}$, and of a second term, $A(\dot{V}/V)$, which describes the influence of the volume changes on the concentration of *A*, i.e.,

$$\begin{array}{c} \dot{A}=\frac{\dot{n_{A}}}{V}-A(\frac{\dot{V}}{V})={\left(\dot{A}\right)}_{V}-A(\frac{\dot{V}}{V}) \end{array}$$ (3)

Eq 3 will be used as a “template” when formulating the rate equations of cellular compounds in the presence of changing *V*. Before we turn to the actual controller examples we show how growth ($\dot{V}$) affects the concentration of a given species *A* (which will be later our controlled variable) when *A* is unreactive, being produced internally within the cell, or being produced by a transporter-mediated process.

### Unreactive *A*

**Fig 2:**
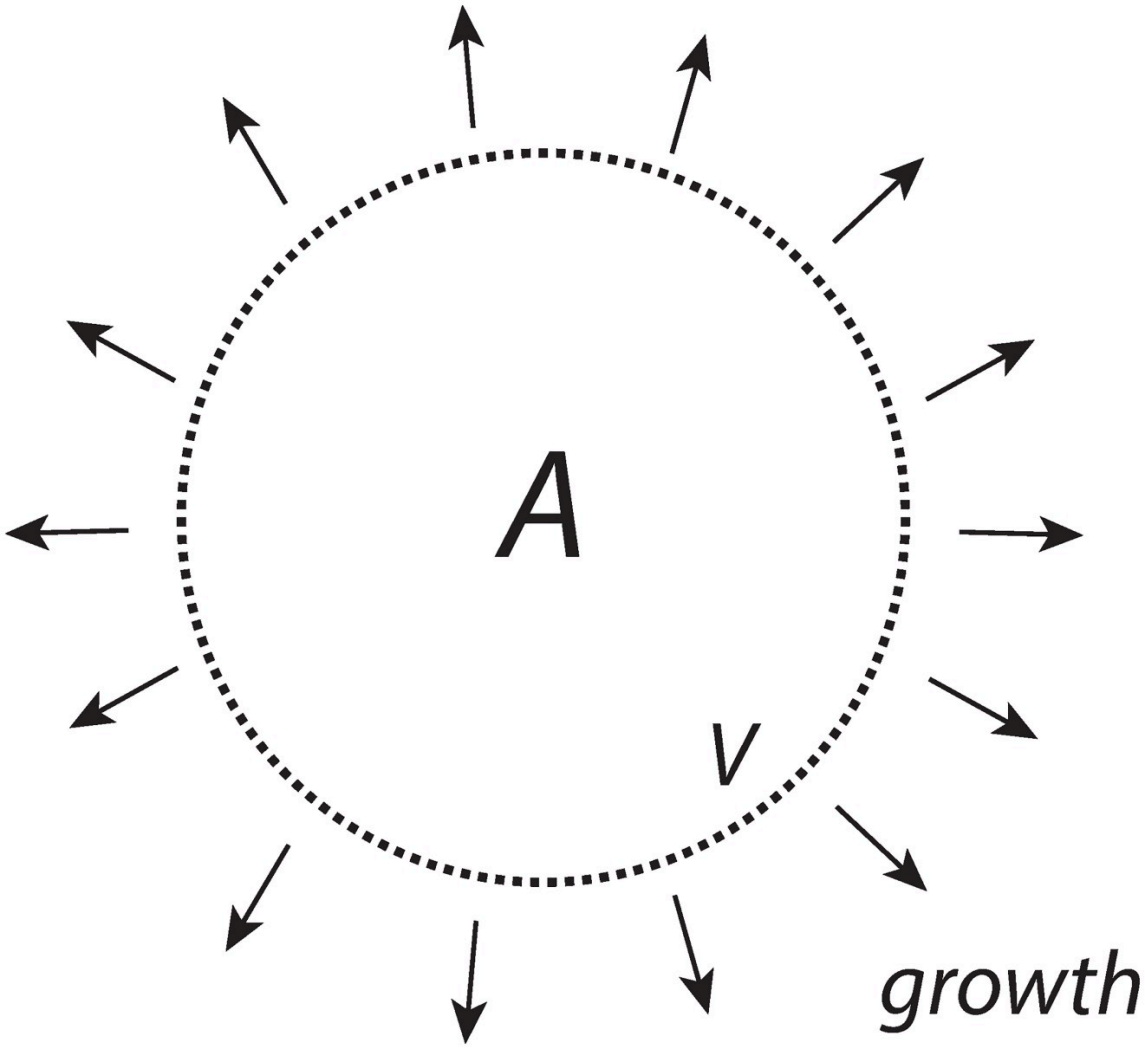
*A* is present inside the cell with a constant amount of *n*_*A*_ moles, while the cellular volume *V* increases with rate $\dot{V}$.

In this example (Fig 2) *n*_*A*_ is kept constant, but the volume *V* increases with rate $\dot{V}$.

As *V* increases the concentration of *A* will decrease, i.e.,

$$\begin{array}{c} A=\frac{n_{A}}{V}\;\;\Rightarrow \;\;\dot{A}=\frac{\dot{n_{A}}}{V}+n_{A}\cdot \frac{\left(\text{d}\frac{1}{V}\right)}{\text{d}t}=\frac{\dot{n_{A}}}{V}-n_{A}\cdot \frac{\dot{V}}{V^{2}}=\frac{\dot{n_{A}}}{V}-A\cdot \frac{\dot{V}}{V} \end{array}$$ (4)

Since we assume that *n*_*A*_ is constant, we have that $\dot{n_{A}}=0$ and the concentration of *A* decreases according to

$$\begin{array}{c} \dot{A}=-A\cdot \frac{\dot{V}}{V}\;\;\Rightarrow \;\;\frac{\dot{A}}{A}=-\frac{\dot{V}}{V}\;\;\Rightarrow \;\;\frac{\text{d}\log(A)}{\text{d}t}=-\frac{\text{d}\log(V)}{\text{d}t} \end{array}$$ (5)

Integrating Eq 5 leads to:

$$\begin{array}{c} \log\left(A\left(t\right)\right)-\log\left(A_{0}\right)-\{\log V\left(t\right)-\log V_{0}\}\;\;\Rightarrow \;\;\log(\frac{A(t)}{A_{0}})=\log(\frac{V_{0}}{V(t)}) \end{array}$$ (6)

which can be rewritten as

$$\begin{array}{c} A\left(t\right)=A_{0}(\frac{V_{0}}{V(t)})\;\;\Leftrightarrow \;\;A\left(t\right)V\left(t\right)=A_{0}V_{0} \end{array}$$ (7)

Eq 7 can also be derived by noting that *A*_0_ = *n*_*A*_/*V*_0_ and *A*(*t*) = *n*_*A*_/*V*(*t*). Solving for *n*_*A*_ from one of the equations and inserting it into the other leads to Eq 7.

### Cell internal generated *A*

In order to counteract diminishing levels of a controlled compound *A* compensatory fluxes can be generated by a cell internal compound (assumed here to be homogeneously distributed inside *V*) or by the help of transporters from stores outside of the cell or from cell-internal (organelle) stores. We will investigate both ways to generate compensatory fluxes.

**Fig 3:**
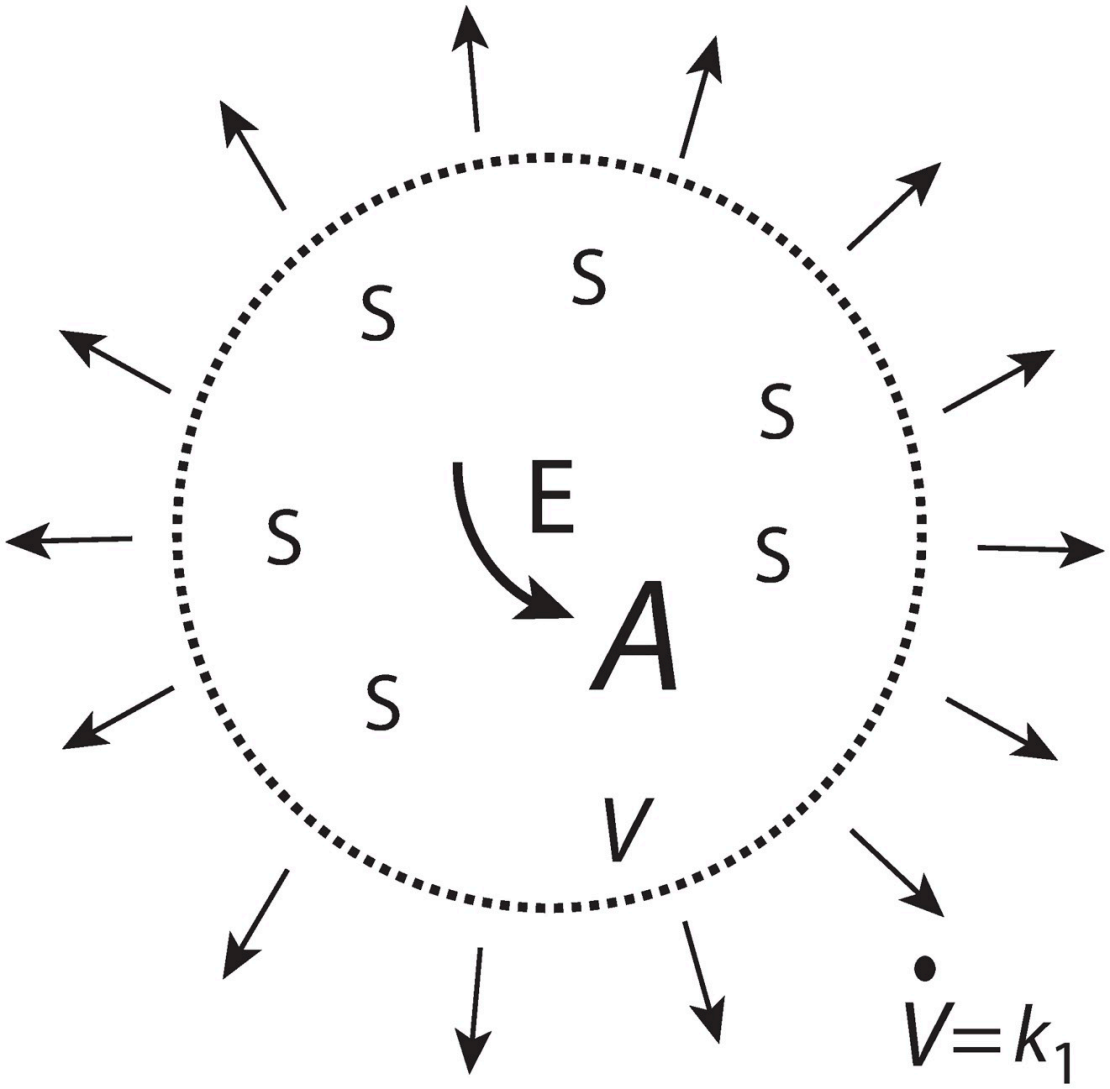
*A* is formed by zero-order kinetics within the cell while the cellular volume increases with a constant rate $\dot{V}=k_{1}$.

To achieve a constant level of *A* from a cell internal source, despite increasing *V*, we consider first a zero-order enzymatic reaction where enzyme *E* converts a species *S* (assumed to be present in sufficiently high amounts) to *A*, where *V* is assumed to increase by a constant rate (Fig 3).

We assume that *E* is not subject to any synthesis, but that during the increase of *V*, *E* remains always saturated with *S* and produces *A* by zero-order kinetics with respect to *A*. The initial production rate of *A* at time *t* = 0 is given as

$$\begin{array}{c} {\dot{A}}_{0}=\frac{v_{max,0}\cdot S_{0}}{K_{M}+S_{0}} \end{array}$$ (8)

Since *E* is considered to be saturated by *S* at all times we have that *K*_*M*_ ≪ *S*(*t*) leading to

$$\begin{array}{c} {\dot{A}}_{0}=v_{max,0}=k_{2}\cdot E_{0} \end{array}$$ (9)

where *k*_2_ is the turnover number of the enzymatic process generating *A*, and *E*_0_ is the enzyme concentration at time *t* = 0. As volume *V* increases, the concentrations of *E* and *A* are subject to dilution as described by the rate equations

$$\begin{array}{c} \dot{E}=-E\cdot \frac{\dot{V}}{V} \end{array}$$ (10)

$$\begin{array}{c} \dot{A}=k_{2}\cdot E-A\cdot \frac{\dot{V}}{V} \end{array}$$ (11)

For $\dot{V}$ = *k*_1_ = constant, *E*(*t*) and *A*(*t*) are described by the equations (S1 Text)

$$\begin{array}{c} E\left(t\right)=E_{0}\cdot \frac{\alpha }{t+\alpha }\;;\;\;\;\;\;\;\alpha =\frac{V_{0}}{k_{1}} \end{array}$$ (12)

$$\begin{array}{c} A\left(t\right)=k_{2}\cdot E_{0}\cdot \alpha -(k_{2}\cdot E_{0}\cdot \alpha -A_{0})\cdot \frac{\alpha }{t+\alpha } \end{array}$$ (13)

From Eq 13 we see that *A* will approach a final concentration *A*_final_ = *k*_2_⋅*E*_0_⋅*α* even when *V* continues to grow. The time needed of *A* to approach *A*_final_ is determined by the term *α*/(*t*+*α*).

**Fig 4:**
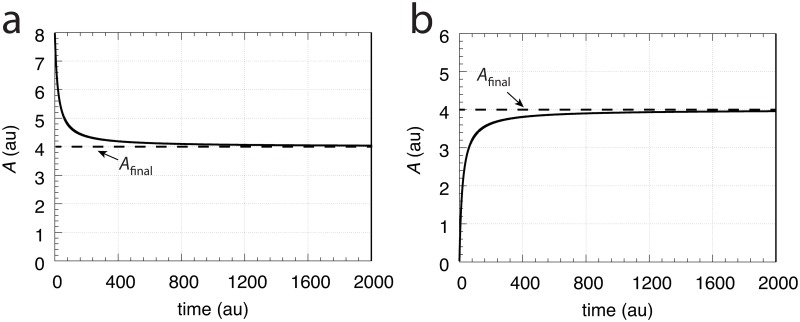
*A* approaches *A*_final_ independent of the initial concentration of *A*. (a) *A*_0_ = 8.0; (b) *A*_0_ = 0.0. All other rate parameters are: $k_{1}=\dot{V}=1.0$, *k*_2_ = 2.0, *E*_0_ = 0.1, *V*_0_ = 20.0.

Fig 4 shows that *A*_final_ is independent of the initial values of *A*. However, the system is not stable against perturbations which remove *A*. In such a case *A* will go to zero (S1 Text).

### Transporter generated *A*

**Fig 5:**
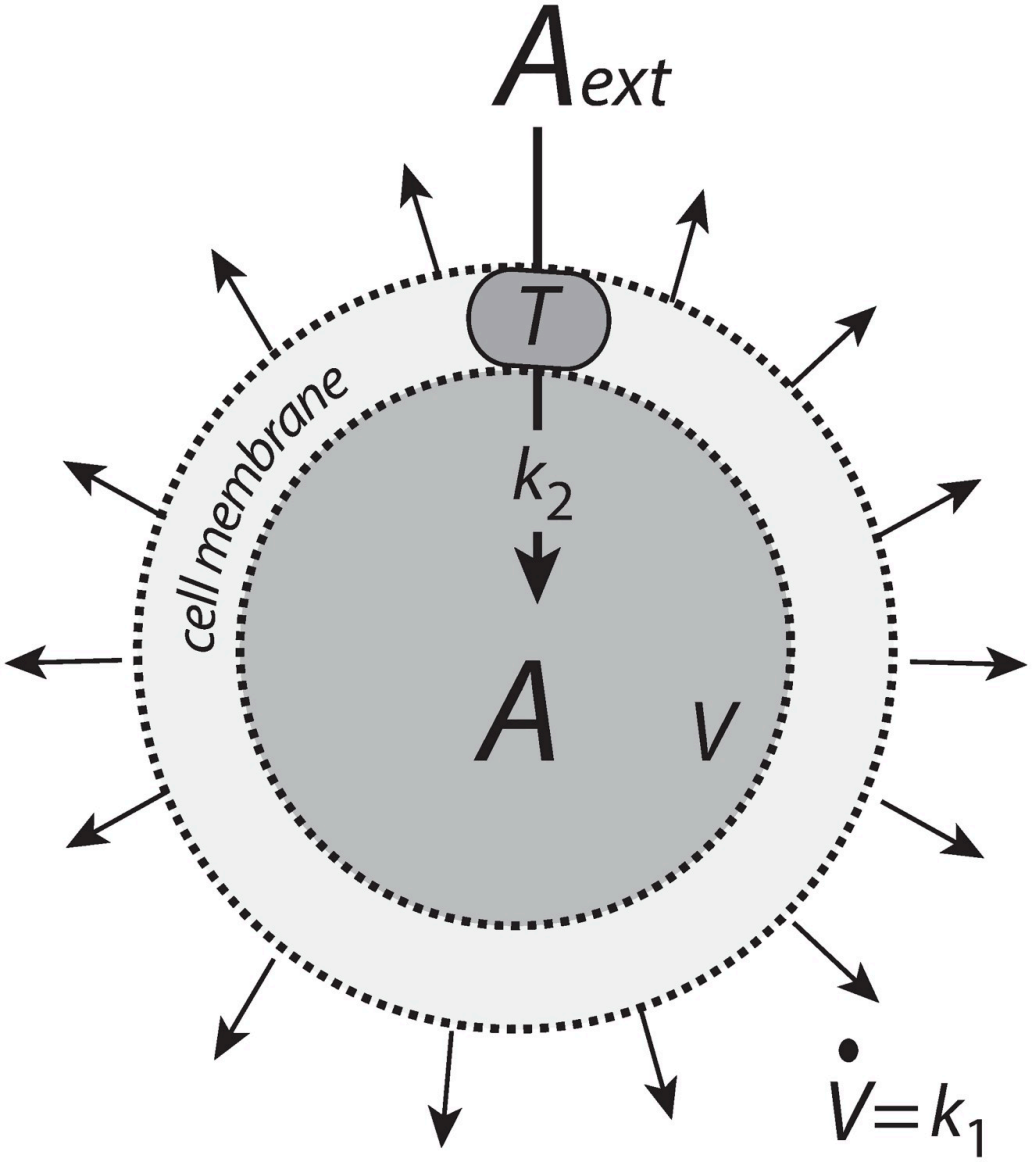
*A* is imported into the cell by transporter *T*.

Alternatively, *A* may be imported into the cell by a transporter *T* (Fig 5).

Also here we consider that the transporter works under saturation (zero-order) conditions adding $\dot{n_{A}}$ moles of *A* per time unit into the cellular volume *V*

$$\begin{array}{c} \dot{n_{A}}=\frac{k_{2}\cdot T\cdot A_{ext}}{K_{M}^{T}+A_{ext}}≃k_{2}\cdot T \end{array}$$ (14)

where *T* denotes the (surface/membrane) concentration of the transporter, $K_{M}^{T}$ is a dissociation constant between external *A* (*A*_*ext*_) and *T*, and *k*_2_ is the turnover number of the transporter-mediated uptake of *A*.

The change in the concentration of *A* inside an expanding cell is given by (see Eq 3)

$$\begin{array}{c} \dot{A}=\frac{\dot{n_{A}}}{V}-A(\frac{\dot{V}}{V})=\frac{k_{2}\cdot T}{V}-A(\frac{\dot{V}}{V}) \end{array}$$ (15)

For constant $\dot{V}$, *k*_2_, and *T* the steady state of *A* ($\dot{A}=0$) is $k_{2}T/\dot{V}$ independent of the initial concentration of *A*. However, also in the transporter-based inflow of *A*, the steady state in *A* is not stable against perturbations removing *A*. Any reaction within the cell removing *A* while growth occurs will drive *A* to zero (S2 Text). To get a steady state that is stable against perturbations a negative feedback controller needs to be included.

## Case A.1: Controllers with transporter-based compensatory fluxes and linear time-dependent perturbations

In this section the four controller motifs (Fig 1) are tested using a transporter-based compensatory flux with respect to constant growth, $\dot{V}=k_{1}$. In addition, an outflow perturbation with a time-dependent rate parameter *k*_3_ is invoked, which removes *A* as a first-order reaction with respect to *A*.

### Motif 1 zero-order controller

**Fig 6:**
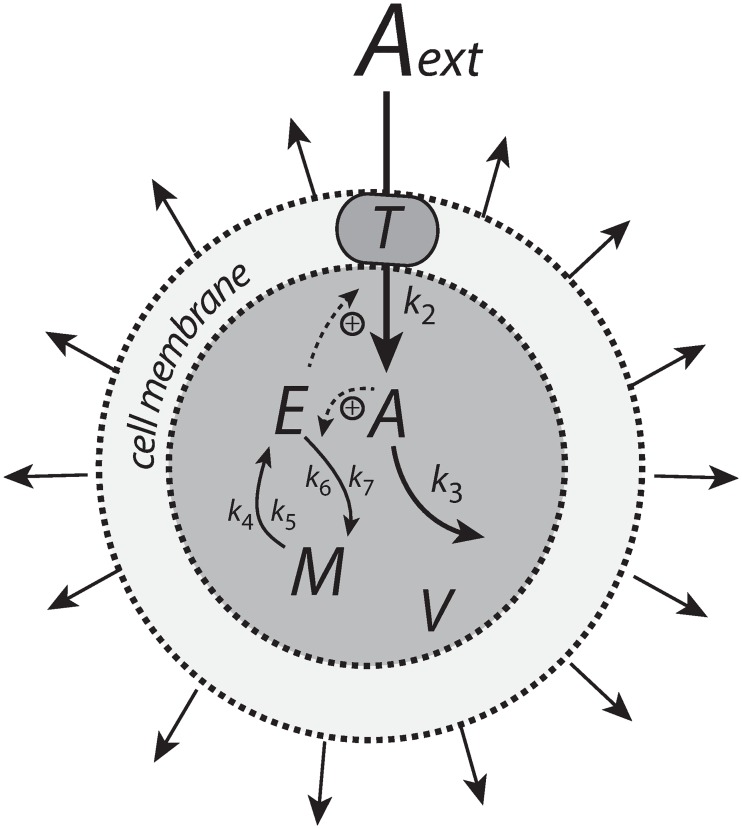
Motif 1 based zero-order integral controller with a transporter (T) generated compensatory flux. The controller species *E* is produced by an enzymatic zero-order process from compound *M*. *E* is recycled by another zero-order process (with respect to *E*) but the rate of *E*-removal is proportional to the concentration of *A*. Outflow perturbations are represented by the rate *r*_3_ = *k*_3_⋅*A*, where *k*_3_ is either constant or increases linearly with time.

Fig 6 shows the motif 1 controller with zero-order implementation of integral control [7]. *A* is the controlled compound and *E* is the controller molecule which concentration (in the ideal controller case) is proportional to the integrated error between *A* and $A_{set}^{theor}$. *M* is considered as a store/precursor into which “consumed” *E* is recycled to. *M* is included to make it explicit that even under recycling conditions the increasing demand for *E* under growth and other time-dependent perturbations leads to a continuous reduction in *M*. This may lead to controller breakdown once all *M* is consumed. A situation when this occurs will be shown below for the motif 1 autocatalytic controller.

The rate equations for this system are:

$$\begin{array}{c} \dot{A}=\frac{\dot{n_{A}}}{V}-k_{3}\cdot A-A(\frac{\dot{V}}{V})=\frac{k_{2}\cdot E\cdot T}{V}(\frac{A_{ext}}{K_{M}^{T}+A_{ext}})-k_{3}\cdot A-A(\frac{\dot{V}}{V}) \end{array}$$ (16)

$$\begin{array}{c} \dot{E}=\frac{k_{4}\cdot M}{k_{5}+M}-(\frac{k_{6}\cdot E}{k_{7}+E})A-E(\frac{\dot{V}}{V}) \end{array}$$ (17)

$$\begin{array}{c} \dot{M}=-\frac{k_{4}\cdot M}{k_{5}+M}+(\frac{k_{6}\cdot E}{k_{7}+E})A-M(\frac{\dot{V}}{V}) \end{array}$$ (18)

For simplicity, *T* and $A_{ext}/\left(K_{M}^{T}+A_{ext}\right)$ are set to 1 leading to an inflow rate in *A* of *k*_2_*E*/*V*. When $\dot{k_{3}}=\dot{V}=0$, the set-point of the controller is (Ref. [7], S3 Text)

$$\begin{array}{c} A_{set}^{theor}=\frac{k_{4}}{k_{6}} \end{array}$$ (19)

independent of the inflow rate constant *k*_2_ and the time-dependent outflow perturbation parameter *k*_3_.

When $\dot{V}$ = constant the zero-order controller maintains a steady state below $A_{set}^{theor}$ (S3 Text):

$$\begin{array}{c} A_{ss}=\frac{k_{4}}{k_{6}+\frac{2\dot{V}k_{3}}{k_{2}}} \end{array}$$ (20)

which is dependent of $\dot{V}$, and the rate constants *k*_2_ and *k*_3_.

**Fig 7:**
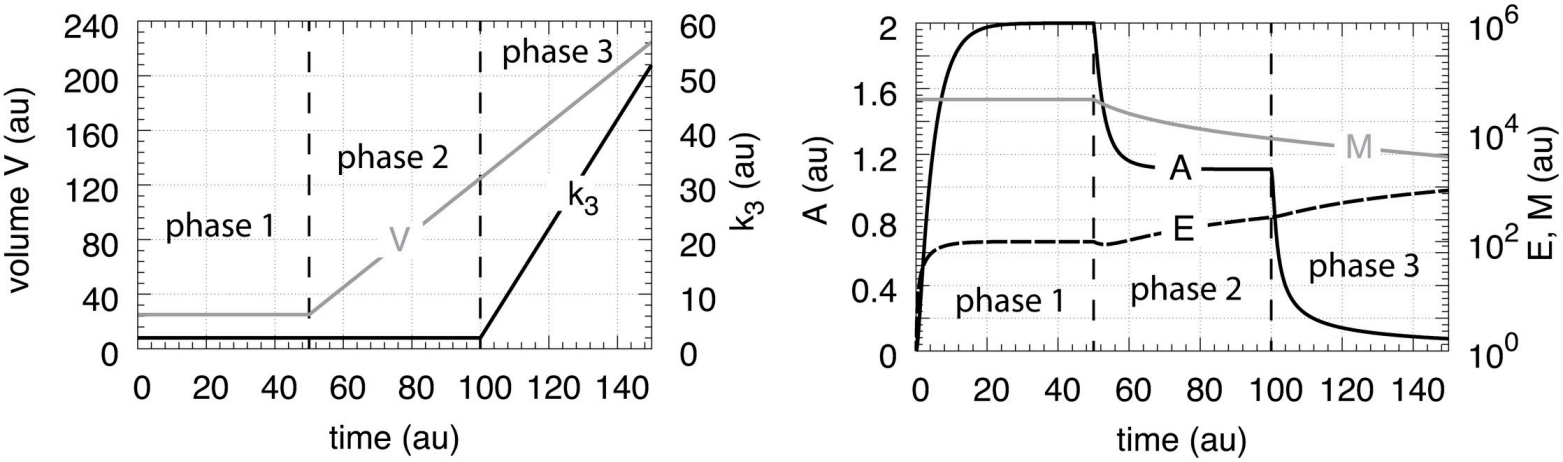
Performance of the motif 1 zero-order controller with transporter mediated compensatory flux (Eqs 16–18). Phase 1: constant volume *V* and constant *k*_3_. Initial concentrations and rate constant values: *V*_0_ = 25.0, $\dot{V}=0.0$, *A*_0_ = 0.0, *E*_0_ = 0.0, *M*_0_ = 4 × 10^4^, *k*_2_ = 1.0, *k*_3_ = 2.0, $\dot{k_{3}}=0.0$, *k*_4_ = 20.0, *k*_5_ = 1 × 10^−6^, *k*_6_ = 10.0, *k*_7_ = 1 × 10^−6^. The controller keeps *A* at its theoretical set-point, $A_{set}^{theor}=k_{4}/k_{6}=2.0$ (Eq 19). Phase 2: rate constants remain the same as in phase 1, but *V* increases linearly with $\dot{V}=2.0$, while *k*_3_ remains constant at *k*_3_ = 2.0. In agreement with Eq 20, the controller shows an offset below $A_{set}^{theor}$ with *A*_*ss*_ = 1.11. Phase 3: *V* continues to increase with the same speed while *k*_3_ starts to increase linearly with $\dot{k_{3}}=1.0$. As indicated by Eq 20 the controller now breaks down and *A* goes to zero as *V* and *k*_3_ increase.

In testing the performance of this controller we consider three phases (see Fig 7). During the first phase the volume and the perturbation *k*_3_ are kept constant. The controller is able to compensate for the perturbation rate *k*_3_⋅*A* and keeps *A* at its theoretical set-point $A_{set}^{theor}$. In the second phase the volume increases linearly with time, while *k*_3_ remains constant. The zero-order controller is now no longer able to maintain homeostasis at $A_{set}^{theor}=k_{4}/k_{6}$, but shows a $\dot{V}$-dependent offset below $A_{set}^{theor}$ as described by Eq 20. When *k*_3_ increases linearly during phase 3 along the increase in *V* the controller breaks down and *A* goes to zero.

### Motif 1 antithetic controller

**Fig 8:**
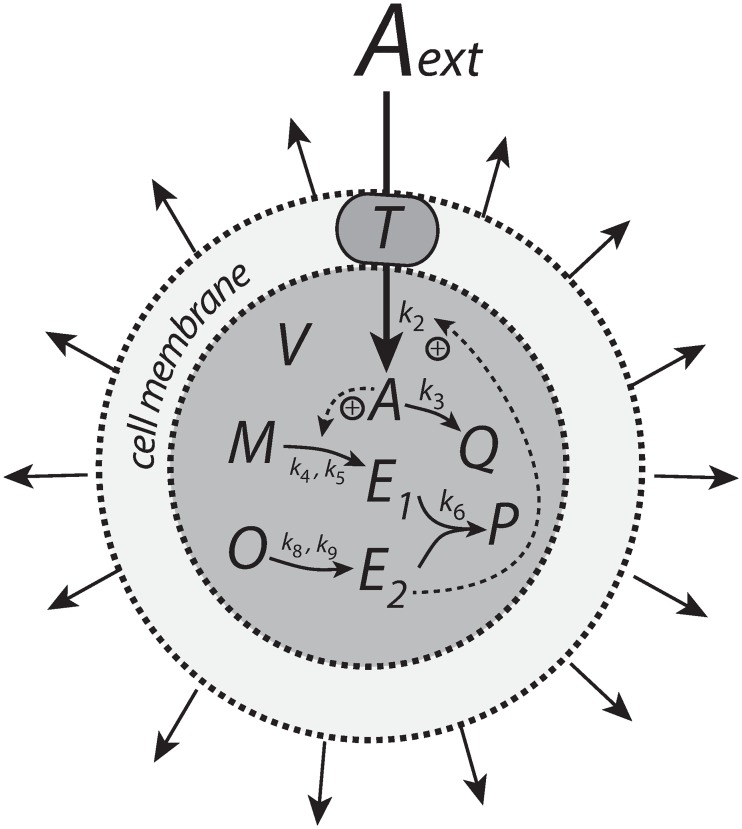
Motif 1 based controller with second-order (antithetic) integral control. The controller species *E*_2_ is produced by an enzymatic zero-order process from compound *O*. *E*_2_ activates the transporter-based compensatory flux of *A* and is removed by *E*_1_ using second-order kinetics forming *P*.

The antithetic controller [22] uses two controller molecules, *E*_1_ and *E*_2_ (Fig 8). Compound *E*_1_ is activated by *A* but is removed by compound *E*_2_ by a second-order process. *E*_2_ is formed by a zero-order process which acts as a constant reference rate. In addition, *E*_2_ also acts as a signaling molecule, which closes the negative feedback loop by activating the transporter-based compensatory inflow of *A*.

Assuming, as in the previous two examples that *T* and $A_{ext}/\left(K_{M}^{T}+A_{ext}\right)$ are both 1, the rate equations are

$$\begin{array}{c} \dot{A}=\frac{\dot{n_{A}}}{V}-k_{3}\cdot A-A\cdot \frac{\dot{V}}{V}=\frac{k_{2}\cdot E_{2}}{V}-k_{3}\cdot A-A(\frac{\dot{V}}{V}) \end{array}$$ (21)

$$\begin{array}{c} \dot{E_{1}}=A(\frac{k_{4}\cdot M}{k_{5}+M})-k_{6}\cdot E_{1}\cdot E_{2}-E_{1}(\frac{\dot{V}}{V}) \end{array}$$ (22)

$$\begin{array}{c} \dot{E_{2}}=\frac{k_{8}\cdot O}{k_{9}+O}-k_{6}\cdot E_{1}\cdot E_{2}-E_{2}(\frac{\dot{V}}{V}) \end{array}$$ (23)

$$\begin{array}{c} \dot{M}=-A(\frac{k_{4}\cdot M}{k_{5}+M})-M(\frac{\dot{V}}{V}) \end{array}$$ (24)

$$\begin{array}{c} \dot{O}=-\frac{k_{8}\cdot O}{k_{9}+O}-O(\frac{\dot{V}}{V}) \end{array}$$ (25)

$$\begin{array}{c} \dot{Q}=k_{3}\cdot A-Q(\frac{\dot{V}}{V}) \end{array}$$ (26)

$$\begin{array}{c} \dot{P}=k_{6}\cdot E_{1}\cdot E_{2}-P(\frac{\dot{V}}{V}) \end{array}$$ (27)

where *k*_5_ ≪ *M* and *k*_9_ ≪ *O* such that the generation of *E*_1_ and *E*_2_ are zero-order processes with respect to *M* and *O*.

**Fig 9:**
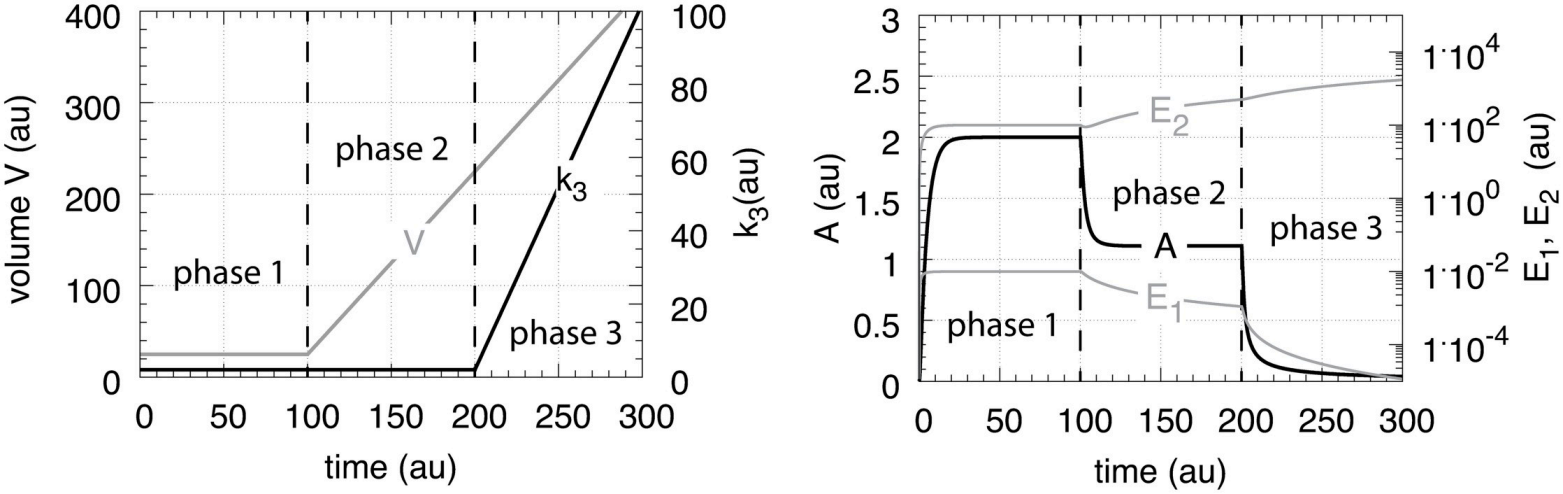
Performance of the antithetic controller with transporter mediated compensatory flux (Eqs 21–27). Phase 1: constant volume *V* and constant *k*_3_. Initial concentrations and rate constant values: *V*_0_ = 25.0, $\dot{V}=0.0$, *A*_0_ = 0.0, *E*_1,0_ = 0.0, *E*_2,0_ = 0.0, *M*_0_ = 1 × 10^5^, *O*_0_ = 1 × 10^5^, *k*_2_ = 1.0, *k*_3_ = 2.0, $\dot{k_{3}}=0.0$, *k*_4_ = 10.0, *k*_5_ = 1 × 10^−6^, *k*_6_ = 20.0, *k*_7_ not used, *k*_8_ = 20.0, *k*_9_ = 1 × 10^−6^. The controller keeps *A* at its theoretical set-point at $A_{set}^{theor}=k_{8}/k_{4}=2.0$ (Eq 28). Phase 2: rate constants remain the same as in phase 1, but *V* increases linearly with $\dot{V}=2.0$, while *k*_3_ remains constant at *k*_3_ = 2.0. The controller shows an offset below $A_{set}^{theor}$ with *A*_*ss*_ = 1.11 in agreement with Eq 29. Phase 3: *V* continues to increase while *k*_3_ increases linearly with $\dot{k_{3}}=1.0$. As indicated by Eq 29 the controller breaks down and *A* goes to zero.

In case $\dot{V}=0$ and $\dot{k_{3}}=0$ the set-point of the controller is given by setting Eqs 22 and 23 to zero. Eliminating the second-order term *k*_6_⋅*E*_1_⋅*E*_2_ leads to

$$\begin{array}{c} A_{set}^{theor}=\frac{k_{8}}{k_{4}}=2.0 \end{array}$$ (28)

which is shown in phase 1 of Fig 9. In phase 2 the volume increases linearly with $\dot{V}=2.0$ (Fig 9, left panel) while *k*_3_ remains to be constant at *k*_3_ = 2.0. The controller is no longer able to keep *A* at its theoretical set-point (Eq 28). When $\dot{V}$ and *k*_3_ are constant an analytical expression of *A*_*ss*_ can be derived in good agreement with the numerical calculations (S4 Text):

$$\begin{array}{c} A_{ss}=\frac{k_{2}k_{8}}{k_{2}k_{4}+2k_{3}\dot{V}} \end{array}$$ (29)

which is analogous to the *A*_*ss*_ expression of the motif 1 zero-order controller (Eq 20). Finally, in phase 3 *k*_3_ increases linearly with $\dot{k_{3}}=1$ together with $\dot{V}=2.0$. As indicated by Eq 29 and shown by the numerical calculations (Fig 9) the antithetic controller, like the zero-order controller, breaks down and *A* goes to zero (S4 Text).

Although not shown explicitly here, the following mass balances are obeyed:

$$\begin{array}{c} n_{M,0}=n_{M}\left(t\right)+n_{E_{1}}\left(t\right)+n_{P}\left(t\right) \end{array}$$ (30)

$$\begin{array}{c} n_{O,0}=n_{O}\left(t\right)+n_{E_{2}}\left(t\right)+n_{P}\left(t\right) \end{array}$$ (31)

where *n*_*i*,0_ and *n*_*i*_ are respectively the initial number of moles and the number of moles at time *t* of compound *i*.

As described above, when using a transporter mediated compensation in *A* the antithetic and the motif 1 zero-order controllers have to increase their controller variables *E*_2_ or *E* in order to keep *A*_*ss*_ constant, as indicated by the equation

$$\begin{array}{c} \dot{A}=0\;\;\Rightarrow \;\;\frac{k_{2}\cdot E_{\left(2\right)}\left(t\right)}{V(t)}=k_{3}\cdot A_{ss} \end{array}$$ (32)

where *E*_(2)_ represents *E*_2_ or *E* and $\left(\dot{V}/V\right)A_{ss}$ becomes negligible.

### Motif 1 autocatalytic controller

Similar to controllers based on double integral action [24] an autocatalytic design [19] is able to keep the controlled species at its set-point even when perturbations become linearly time dependent and rapid [18]. However, in contrast to double integral action the autocatalytic controller is able to compensate for time-dependent perturbations of the form *a*⋅*t*^*n*^ where *n* is larger than 1.

**Fig 10:**
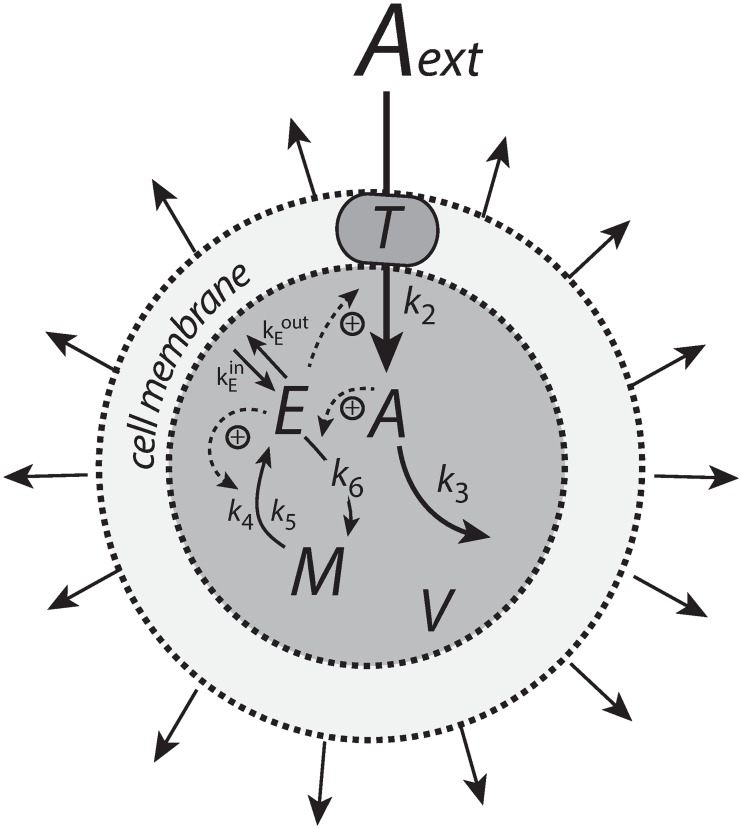
Motif 1 autocatalytic integral controller. The controller species *E* is produced by an enzymatic zero-order process from compound *M*, but *E* activates its own production and the transporter-based compensatory flux. The negative feedback is due to the inflow activation of *A* by *E* through transporter *T*, while *A* activates the (first-order) recycling of *E* to *M*. Outflow perturbation in *A* is described by the rate *k*_3_⋅*A*, where *k*_3_ is either a constant or increases linearly with time. $k_{E}^{in}$ and $k_{E}^{out}$ represent background reactions creating and removing *E*.

Fig 10 shows the reaction scheme. The controller compound *E* is produced autocatalytically, i.e., its rate is proportionally to the concentration of *E*, while *M*, present in relative large amounts, produces *E* by an enzyme-catalyzed reaction which is zero-order with respect to *M*. *E* increases the activity of transporter *T* and leads to an increased import of external *A* into the cell. The negative feedback is closed by an *A*-induced recycling of *E* to *M*. Rate constant *k*_3_ represents a perturbation which removes *A* by a first-order process with respect to *A*. The rate equations are:

$$\begin{array}{c} \dot{A}=\frac{\dot{n_{A}}}{V}-k_{3}\cdot A-A(\frac{\dot{V}}{V})=\frac{k_{2}\cdot E\cdot T}{V}(\frac{A_{ext}}{K_{M}^{T}+A_{ext}})-k_{3}\cdot A-A(\frac{\dot{V}}{V}) \end{array}$$ (33)

$$\begin{array}{c} \dot{E}=E(\frac{k_{4}\cdot M}{k_{5}+M})-k_{6}\cdot E\cdot A-E(\frac{\dot{V}}{V})+k_{E}^{in}-k_{E}^{out}\cdot E \end{array}$$ (34)

$$\begin{array}{c} \dot{M}=-E(\frac{k_{4}\cdot M}{k_{5}+M})+k_{6}\cdot E\cdot A-M(\frac{\dot{V}}{V}) \end{array}$$ (35)

**Fig 11:**
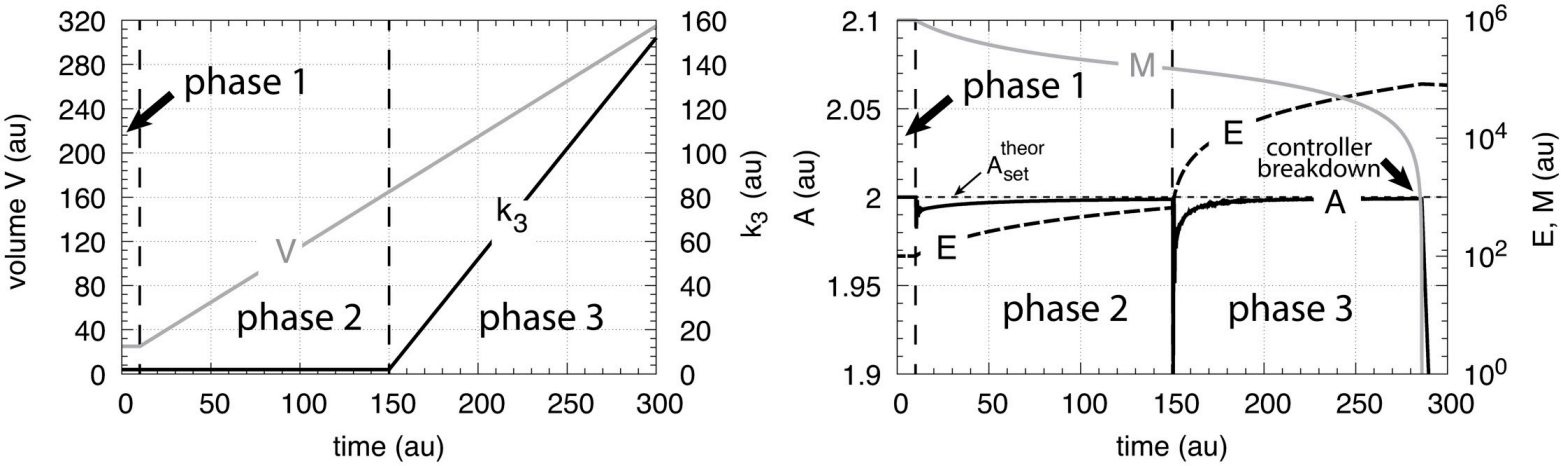
Performance of the motif 1 autocatalytic controller (Eqs 33–35). Phase 1: constant volume *V* and constant *k*_3_. Initial concentrations and rate constant values (at the controller’s steady state): *V*_0_ = 25.0, $\dot{V}=0.0$, *A*_0_ = 2.0, *E*_0_ = 100.0, *M*_0_ = 1 × 10^6^, *k*_2_ = 1.0, *k*_3_ = 2.0, $\dot{k_{3}}=0.0$, *k*_4_ = 20.0, *k*_5_ = 1 × 10^−6^, *k*_6_ = 10.0, $k_{E}^{in}=k_{E}^{out}=1\times 10^{-5}$. The controller keeps *A* at its set-point at $A_{set}^{theor}=k_{4}/k_{6}=2.0$. Phase 2: rate constants remain the same as in phase 1, but *V* increases linearly with $\dot{V}=1.0$. Phase 3: *V* continues to increase with the same rate and *k*_3_ increases with rate $\dot{k_{3}}=1.0$. The controller moves *A* towards $A_{set}^{theor}$ in both phase 2 and phase 3, but breaks down when no additional *E* becomes available through *M* (indicated by the arrow in the right panel).

As in the previous cases, in Eq 33, the term $T\cdot A_{ext}/\left(K_{M}^{T}+A_{ext}\right)$ is set to 1. The last two terms in Eq 34, $k_{E}^{in}-k_{E}^{out}\cdot E$, represent required background reactions to keep *E* at a sufficiently high level such that the autocatalysis in *E* can start at low/zero initial *E* concentrations (see also Ref. [18] and Discussion there). In the calculations presented here, $k_{E}^{in}$ and $k_{E}^{out}$ are set to 1×10^−5^. To show that in this case the controller can start from initial concentration *E*_0_ = 0, see the corresponding calculation later in the paper when using a cell-internal compensatory flux, or test it using S1 Matlab for Fig 11. When *E*_0_ is larger than 10^−5^ the $k_{E}^{in}-k_{E}^{out}\cdot E$ term is not needed, but its presence will not affect controller dynamics or set-point as long as $k_{E}^{in}$ and $k_{E}^{out}$ are kept low. In case the $k_{E}^{in}$ and $k_{E}^{out}$ values are higher, a change/reduction in the set-point is observed, which the controller still defends (see later in this chapter).

To determine the controller’s set-point at constant *V* and *k*_3_ we set Eq 34 to zero. Neglecting the $k_{E}^{in}-k_{E}^{out}\cdot E$ term and setting $\dot{V}=0$, we can solve for the steady state value of *A*, which defines the controller’s theoretical set-point $A_{set}^{theor}$:

$$\begin{array}{c} \dot{E}=E_{ss}(\frac{k_{4}\cdot M}{k_{5}+M})-k_{6}\cdot E_{ss}\cdot A_{ss}=E_{ss}[(\frac{k_{4}\cdot M}{k_{5}+M})-k_{6}\cdot A_{ss}]=0 \end{array}$$ (36)

Since *M*/(*k*_5_ + *M*) = 1 (ideal zero-order conditions), we get from Eq 36

$$\begin{array}{c} k_{4}-k_{6}\cdot A_{ss}=0\;\;\Rightarrow \;\;A_{ss}=A_{set}^{theor}=\frac{k_{4}}{k_{6}} \end{array}$$ (37)

For constant $\dot{V}$ and $\dot{k_{3}}$ values the set-point is calculated to be (S5 Text)

$$\begin{array}{c} A_{ss}=\frac{k_{4}}{k_{6}}-\frac{\dot{k_{3}}}{k_{6}\cdot k_{3}}\to \frac{k_{4}}{k_{6}}=A_{set}^{theor}\;\;\text{as}\;\;t\to \infty \end{array}$$ (38)

According to previous findings on the autocatalytic controller [18], any time-dependent function *k*_3_(*t*) = *k*_3,0_ + *a*⋅*t*^*n*^ where *a*, *n* > 0 will lead to the set-point conditions described by Eq 38 (S5 Text).

The recycling scheme between *E* and *M* implies that *E* and *M* obey a mass balance of the form

$$\begin{array}{c} n_{E}\left(t\right)+n_{M}\left(t\right)=n_{E,0}+n_{M,0} \end{array}$$ (39)

with *n*_*E*_(*t*) = *E*(*t*)⋅*V*(*t*), *n*_*M*_(*t*) = *M*(*t*)⋅*V*(*t*), and where *n*_*E*,0_ and *n*_*M*,0_ are the initial number of moles of respectively *E* and *M*. The rates how *n*_*E*_ and *n*_*M*_ change at a given time *t* are given as (S5 Text)

$$\begin{array}{c} \dot{n_{E}}=[\dot{E}+E(\frac{\dot{V}}{V})]\cdot V=-\dot{n_{M}}=-[\dot{M}+M(\frac{\dot{V}}{V})]\cdot V \end{array}$$ (40)

Fig 11 shows the results. During the first phase no volume change occurs and *k*_3_ is a constant. The controller keeps *A* at $A_{set}^{theor}=2.0$ as described by Eq 37. During the second phase both *V* and *k*_3_ increase linearly and the controller still keeps *A* at $A_{set}^{theor}=2.0$ according to Eq 38. To keep *A* at its set-point during increasing *V* and/or *k*_3_ the concentration of *E* has to increase in order to maintain the steady state condition given by Eq 33 when $\dot{A}=0$ and $\dot{V}/V\to 0$, i.e.,

$$\begin{array}{c} E\left(t\right)=\frac{k_{3}\left(t\right)\cdot V\left(t\right)\cdot A_{ss}}{k_{2}} \end{array}$$ (41)

From the initial conditions (see legend of Fig 11) we have that *n*_*E*_(*t*) + *n*_*M*_(*t*) = *V*_0_ ⋅ *M*_0_ = 2.5 × 10^7^.

When $k_{E}^{in}$ and $k_{E}^{out}$ are significantly higher than 10^−5^, then the set-point of the controller changes to the following steady state value in *A*:

$$\begin{array}{c} A_{ss}\approx \frac{k_{4}-k_{E}^{out}}{k_{6}} \end{array}$$ (42)

The new set-point is defended by the controller for step-wise changes and for linearly increasing values of *k*_3_ and *V* (for details, see S5 Text).

### Motif 2 zero-order controller

**Fig 12:**
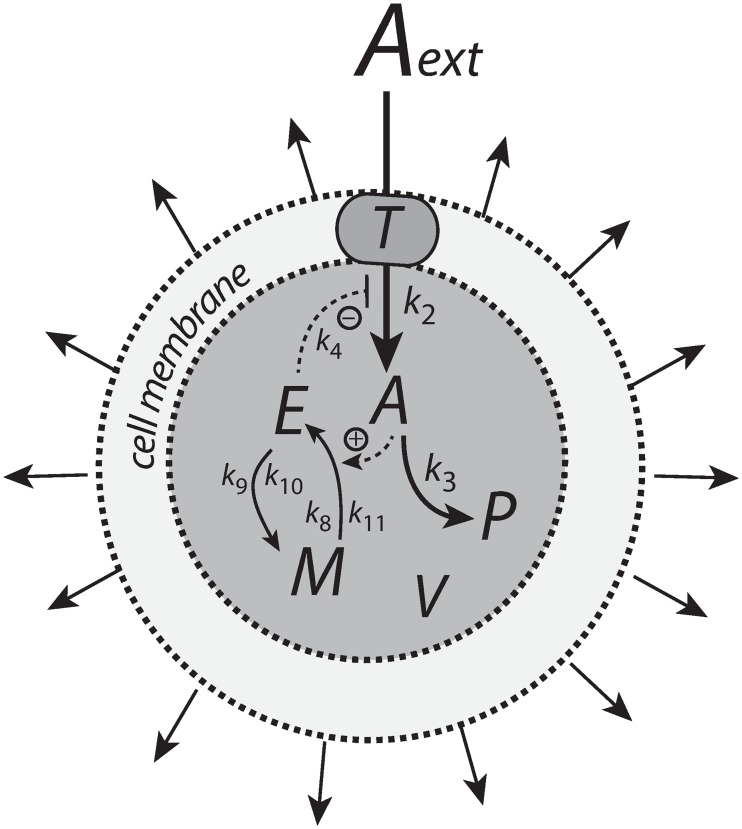
Motif 2 based controller with zero-order integral control. An increase of the compensatory flux occurs by a decrease of *E* (derepression of the compensatory flux).

The reaction scheme of this controller is shown in Fig 12. The transporter-based compensatory flux is regulated by *E* through repression or derepression by *E*. *E* is removed by a zero-order reaction creating M, which then is recycled in a *A*-dependent manner.

The rate equations are

$$\begin{array}{c} \dot{A}=\frac{\dot{n_{A}}}{V}-k_{3}\cdot A-A(\frac{\dot{V}}{V})=\frac{k_{2}k_{4}}{k_{4}+E}(\frac{T\cdot A_{ext}}{K_{M}^{T}+A_{ext}})\cdot \frac{1}{V}-k_{3}\cdot A-A(\frac{\dot{V}}{V}) \end{array}$$ (43)

$$\begin{array}{c} \dot{E}=(\frac{k_{8}\cdot M}{k_{11}+M})\cdot A-\frac{k_{9}\cdot E}{k_{10}+E}-E(\frac{\dot{V}}{V}) \end{array}$$ (44)

$$\begin{array}{c} \dot{M}=-(\frac{k_{8}\cdot M}{k_{11}+M})\cdot A+\frac{k_{9}\cdot E}{k_{10}+E}-M(\frac{\dot{V}}{V}) \end{array}$$ (45)

$$\begin{array}{c} \dot{P}=k_{3}\cdot A-P(\frac{\dot{V}}{V}) \end{array}$$ (46)

Also here, we keep for the sake of simplicity, $T\cdot A_{ext}/\left(K_{M}^{T}+A_{ext}\right)=1$. In presence of growing *V* and *k*_3_ the motif 2 zero-order controller successfully defends its theoretical set-point given by (S6 Text)

$$\begin{array}{c} A_{set}^{theor}=\frac{k_{9}}{k_{8}} \end{array}$$ (47)

However, since an increase of the compensatory flux is based on derepression by *E* (decreasing *E*), the controller will break down when *E* ≪ *k*_4_ or *k*_4_/(*k*_4_+*E*)≈1. Neglecting the $A\cdot \dot{V}/V$ term, the point when the breakdown occurs can be estimated by setting Eq 43 to zero

$$\begin{array}{c} \dot{A}=\frac{k_{2}}{V}-k_{3}\cdot A_{set}^{theor}=0\;\;\Rightarrow \;\;k_{3}\cdot V=\frac{k_{2}}{A_{set}^{theor}} \end{array}$$ (48)

**Fig 13:**
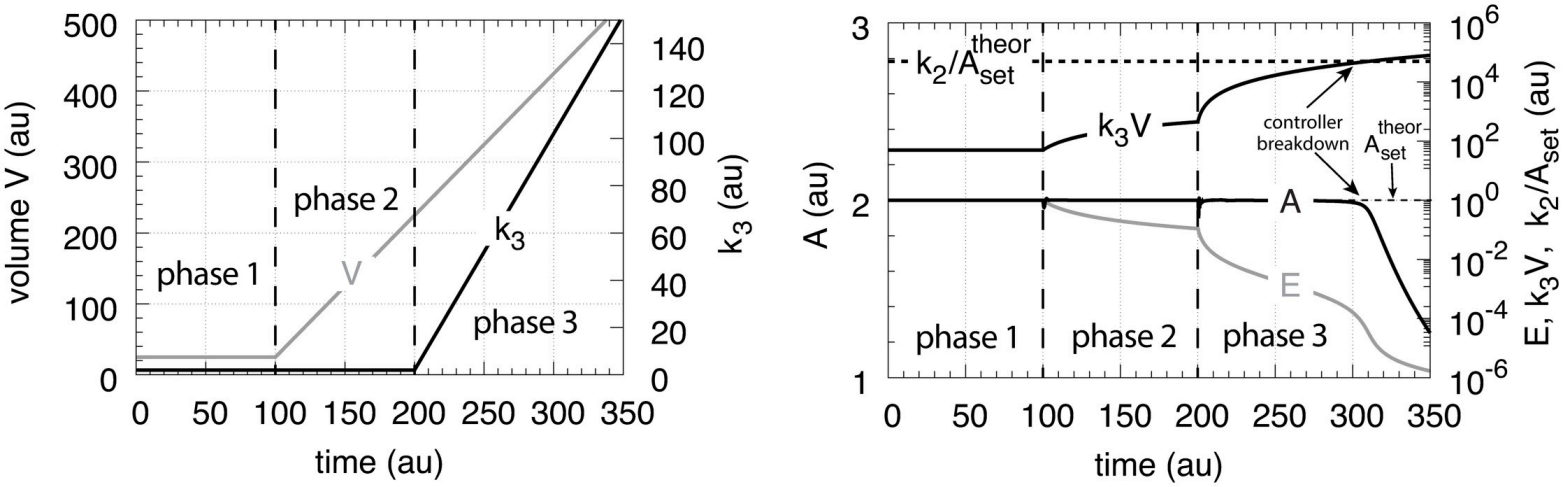
Performance of the motif 2 zero-order based controller with respect to linear increases in *V* and *k*_3_. The controller is able to defend $A_{set}^{theor}$ successfully, but breaks down when *k*_3_*V* reaches $k_{2}/A_{set}^{theor}$ (Eq 48). Rate parameters: *k*_2_ = 1 × 10^5^, *k*_4_ = 1 × 10^−3^, *k*_8_ = 1.0, *k*_9_ = 2.0, *k*_10_ = *k*_11_ = 1 × 10^−6^. Initial conditions: $A_{0}=A_{set}^{theor}=2.0$, *E*_0_ = 1.0, *M*_0_ = 1 × 10^6^, *P*_0_ = 0.0, *V*_0_ = 25.0, *k*_3,0_ = 2.0. $\dot{V}=2.0$ (phase 2 and phase 3), $\dot{k_{3}}=1.0$ (phase 3).

Fig 13 shows that the motif 2 based controller is able to defend successfully against linear growth in both *V* and *k*_3_ and keeping *A* at $A_{set}^{theor}$. Prolonged time intervals with increasing *V* and *k*_3_ will lead to controller breakdown when the condition of Eq 48 is met. The condition *k*_4_/(*k*_4_+*E*)≈1 also indicates that the capacity limit of the controller has been reached, because the compensatory flux *k*_2_ *k*_4_/(*k*_4_+*E*) (Eq 43) has reached its maximum value *k*_2_ and can no longer be increased.

## Case A.2: Controllers with transporter-based compensatory fluxes and exponential time-dependent perturbations

**Fig 14:**
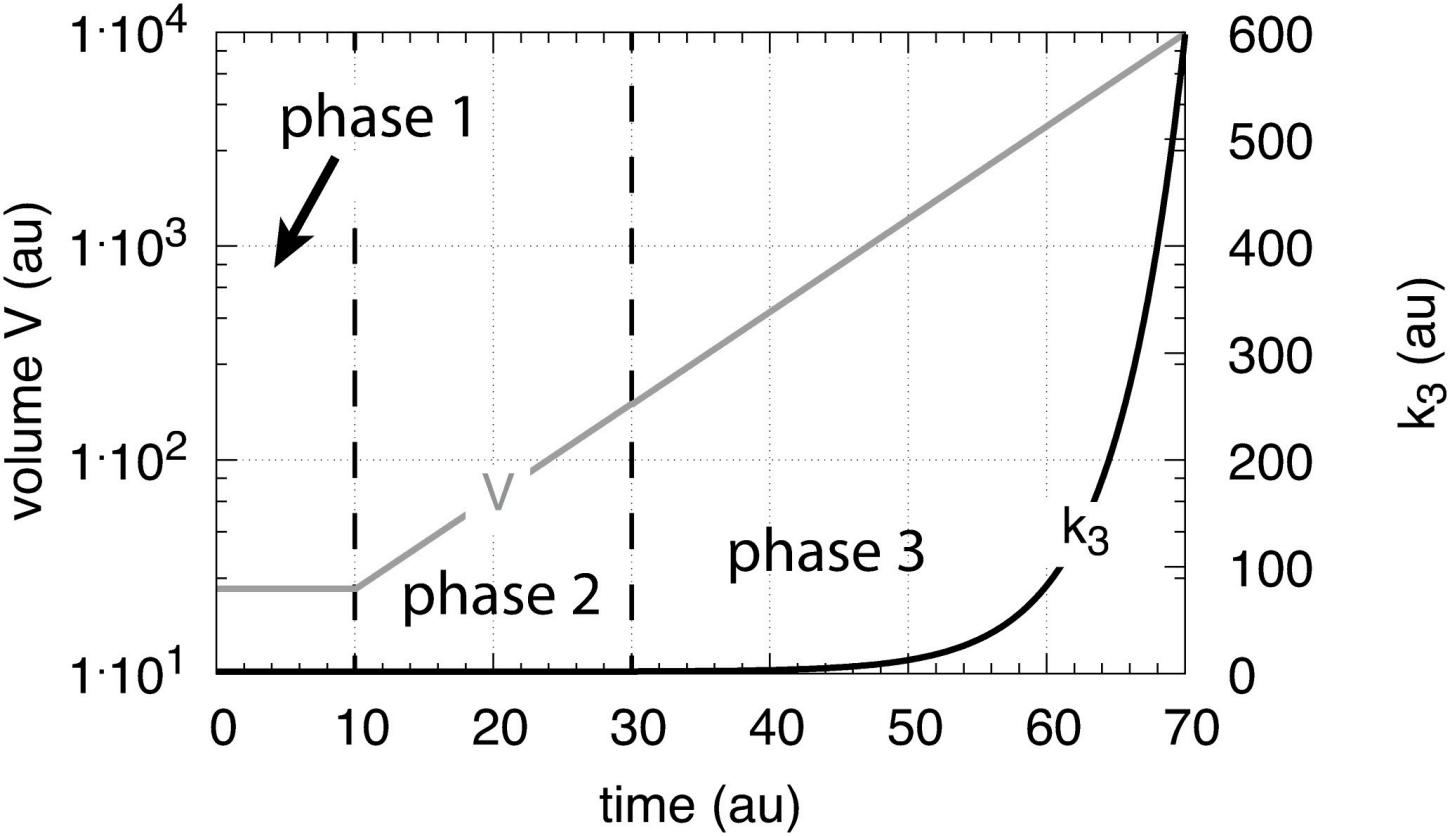
The perturbation profile with exponential growth of *V* and *k*_3_. Due to presentation reasons *V* is plotted semi-logarithmically while the *k*_3_ scale is linear.

Here we describe the performance of the four controller motifs (Fig 1) with transporter-based compensatory fluxes when exposed to exponential growth, $\dot{V}=\kappa \cdot V$, and an exponential increase in the outflow perturbation rate parameter *k*_3_ (Fig 14).

There are three phases the controllers are exposed to. During the first phase the controllers are at their steady states and *V* and *k*_3_ are kept constant at respectively 25.0 and 2.0. During the second phase *V* increases exponentially according to $\dot{V}=\kappa V$ (*κ* = 0.1), while *k*_3_ is kept constant at 2.0. During phase 3, *V* continues to grow exponentially and *k*_3_ starts to increase according to

$$\begin{array}{c} k_{3}\left(t\right)=k_{3,p3}+0.2({\text{e}}^{0.2(t-t_{p3})}-1) \end{array}$$ (49)

where *k*_3,*p*3_ and *t*_*p*3_ are the values of respectively *k*_3_ and time *t* at the beginning of phase 3.

**Fig 15:**
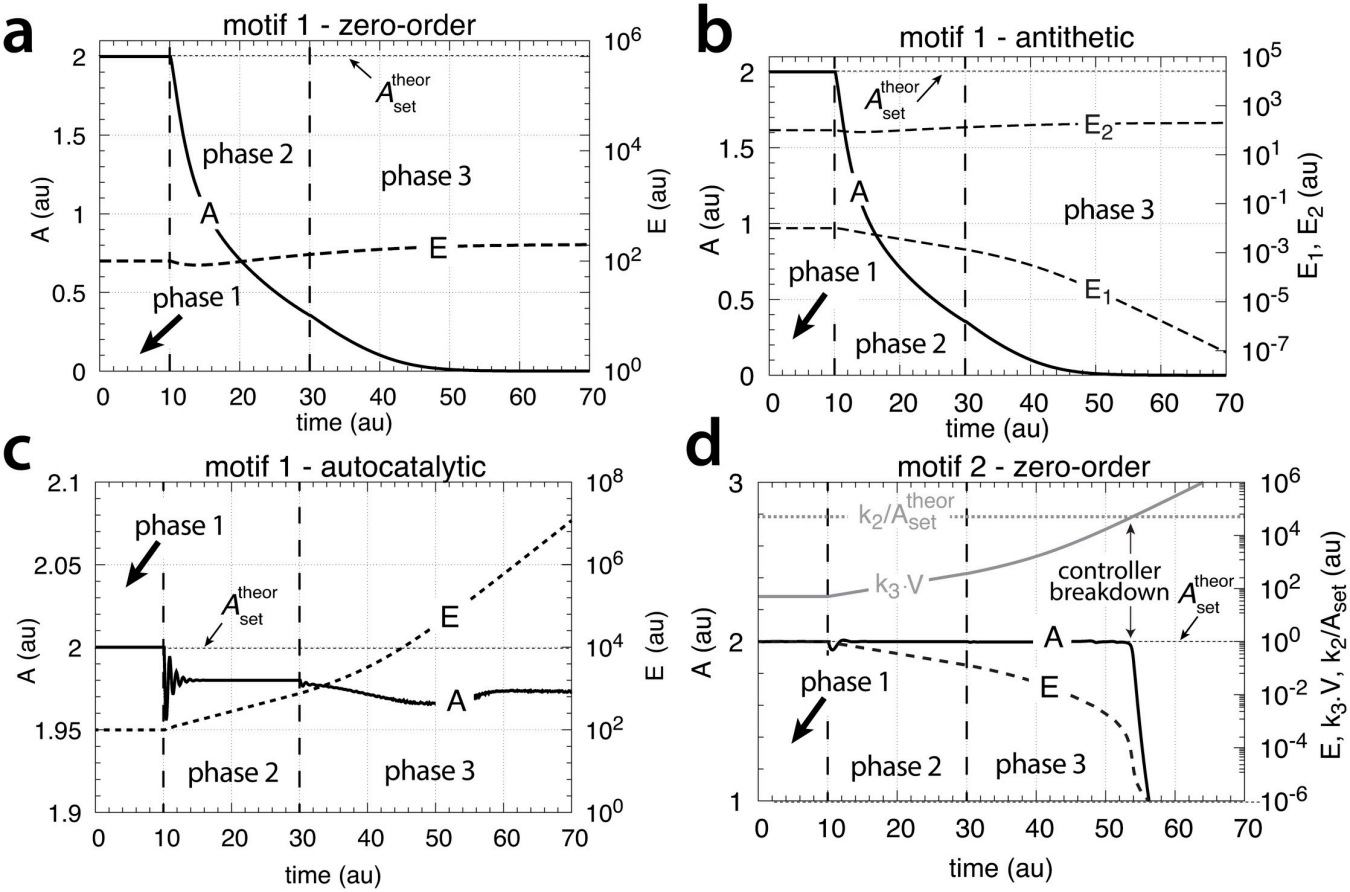
Performance of the (a) motif 1-zero-order, (b) -antithetic, (c) -autocatalytic, and (d) motif 2 zero-order controllers with transporter-based compensatory fluxes in relation to the perturbation profile of Fig 14. For rate equations of the individual controllers, see the descriptions in the previous sections dealing with linear time-dependent perturbations. Rate parameters and initial conditions: (a) see legend of Fig 7, (b) see Fig 9, (c) see Fig 11, but using *M*_0_ = 1×10^10^, and (d) see Fig 13.

Fig 15 shows that only the motif 2 based controller with derepression kinetics (panel d) is able to counteract both exponential increases in *V* and *k*_3_. However, due to the derepression kinetics and due to the transporter based kinetics (see Eq 48) the controller breaks down when the product of the perturbations, *k*_3_*V* reaches $k_{6}/A_{set}^{theor}$. The motif 1 autocatalytic controller (panel c) shows slight constant offsets below $A_{set}^{theor}$, as expected [18], both for the single exponential increase of *V* during phase 2 and when both *V* and *k*_3_ increase exponentially in phase 3. These offsets increase when the values of $k_{E}^{in}$ and $k_{E}^{out}$ are large and cannot be neglected (S5 Text). Since *E* increases with increasing perturbation strengths the controller is limited by the supply for *E* via *M* as indicated in Fig 11. Neither the motif 1 based zero-order controller (panel a) nor the antithetic controller based on motif 1 (panel b) are able to compensate for exponentially increasing perturbation strengths. They behave very similar, as already seen in Figs 7 and 9 for linear time-dependent perturbations.

### Growth related to surface to volume ratio and controllers with transporter-based compensatory fluxes

**Fig 16:**
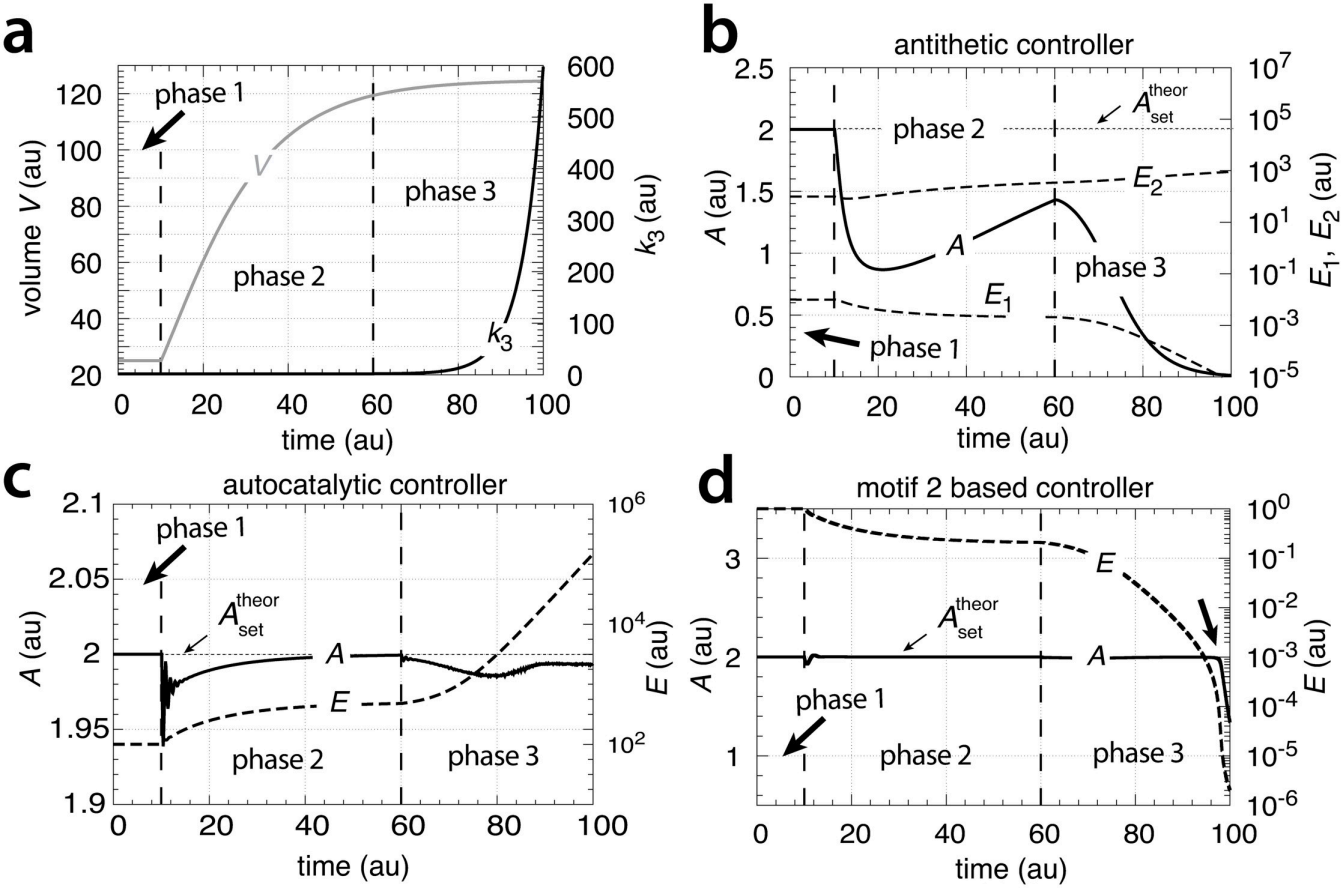
Performance of the motif 1 antithetic, motif 1 autocatalytic and motif 2 zero-order controllers with respect to surface to volume ratio related growth and an exponential increase of *k*_3_. (a) Perturbation profile. Phase 1: constant *V* (25.0) and *k*_3_ (2.0); phase 2: *V* increases according to Eq 2 (*η* = 1 and *ξ* = 0.2) and *k*_3_ remains constant; phase 3: *V* continues to increase and *k*_3_ starts to increase exponentially as described by Eq 49. (b) Behavior of the antithetic controller (Eqs 21–27). Rate constant values as in Fig 9. Initial concentrations: *A*_0_ = 2.0, *E*_1,0_ = 0.01, *E*_2,0_ = 100, *M*_0_ = *O*_0_ = 1 × 10^6^. (c) Behavior of the autocatalytic controller (Eqs 33–35). Rate constant values as in Fig 11. Initial concentrations: *A*_0_ = 2.0, *E*_0_ = 0.01, *M*_0_ = 1 × 10^6^. (d) Behavior of the motif 2 zero-order controller (Eqs 43–46). Initial concentrations: *A*_0_ = 2.0, *E*_0_ = 1.0, *M*_0_ = 1 × 10^3^. Note the breakdown of the controller at the very end of phase 3 due to low *E* (arrow).

We have investigated how the controllers with transporter-based compensatory fluxes behave with respect to the growth law described by Eq 2 (*η* = 1 and *ξ* = 0.2) when *k*_3_ increases exponentially in phase 3 according to Eq 49 (Fig 16a).

Fig 16b–16d show the results of the antithetic, motif 1 autocatalytic and motif 2 zero-order controllers. The motif 1 zero-order controller’s behavior of *A* is identical to that of the antithetic controller and only the result of the antithetic controller is shown. Typically for this type of growth law is that the motif 1 based controllers gain successively control during phase 2 when $\dot{V}$ decreases and approaches zero. During phase 3, when *k*_3_ increases exponentially, only the motif 2 based is able to defend its theoretical set-point, but breaks down when *E* become too low. The autocatalytic controller shows a constant offset below $A_{set}^{theor}$. Both the antithetic and the motif 1 zero-order controllers break down during phase 3 and *A* goes to zero.

## Case B.1: Controllers with cell-internal compensatory fluxes and linear time-dependent perturbations

We consider here the four controllers, but the compensatory fluxes are now generated from cell-internal and homogeneously distributed sources.

### Motif 1 zero-order controller

**Fig 17:**
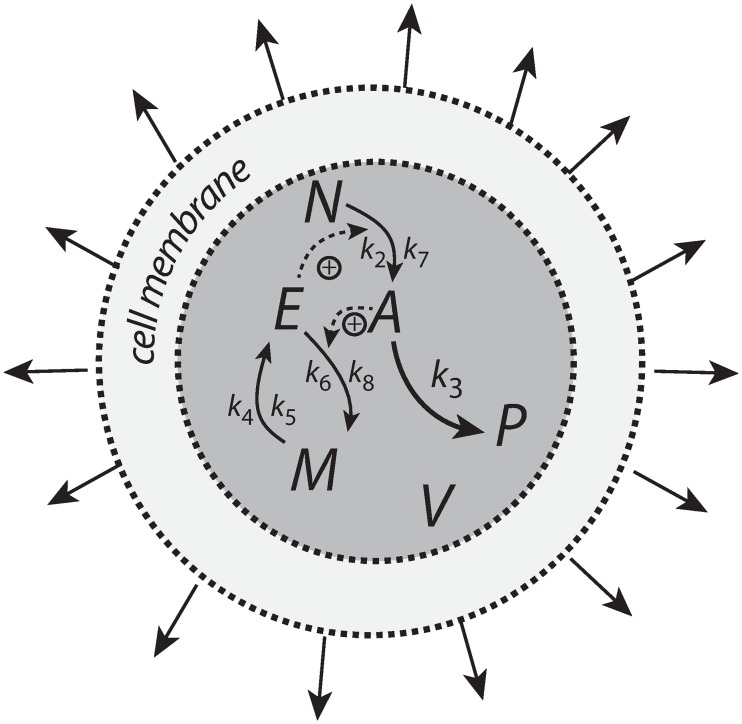
Motif 1 zero-order controller with a cell-internal compensatory flux.

Fig 17 shows the motif 1 zero-order controller using a cell-internal compensatory flux. The homogenously distributed compound *N* serves as a source for *A*, which is activated by *E*. Compound *M* serves as a source for *E*, while by the activation of *A*, *M* is recycled from *E*.

The rate equations are

$$\begin{array}{c} \dot{A}=k_{2}\cdot E(\frac{N}{k_{7}+N})-k_{3}\cdot A-A(\frac{\dot{V}}{V}) \end{array}$$ (50)

$$\begin{array}{c} \dot{E}=\frac{k_{4}\cdot M}{k_{5}+M}-(\frac{k_{6}\cdot E}{k_{8}+E})A-E(\frac{\dot{V}}{V}) \end{array}$$ (51)

$$\begin{array}{c} \dot{M}=-\frac{k_{4}\cdot M}{k_{5}+M}+(\frac{k_{6}\cdot E}{k_{8}+E})A-M(\frac{\dot{V}}{V}) \end{array}$$ (52)

$$\begin{array}{c} \dot{N}=-(\frac{k_{2}\cdot N}{k_{7}+N})E-N(\frac{\dot{V}}{V}) \end{array}$$ (53)

$$\begin{array}{c} \dot{P}=-k_{3}\cdot A-P(\frac{\dot{V}}{V}) \end{array}$$ (54)

The steady state of *A* when both $\dot{V}$ and $\dot{k_{3}}$ are constant is given by the following expression (S3 Text)

$$\begin{array}{c} A_{ss}=\frac{k_{2}k_{4}}{k_{2}k_{6}+\dot{k_{3}}} \end{array}$$ (55)

**Fig 18:**
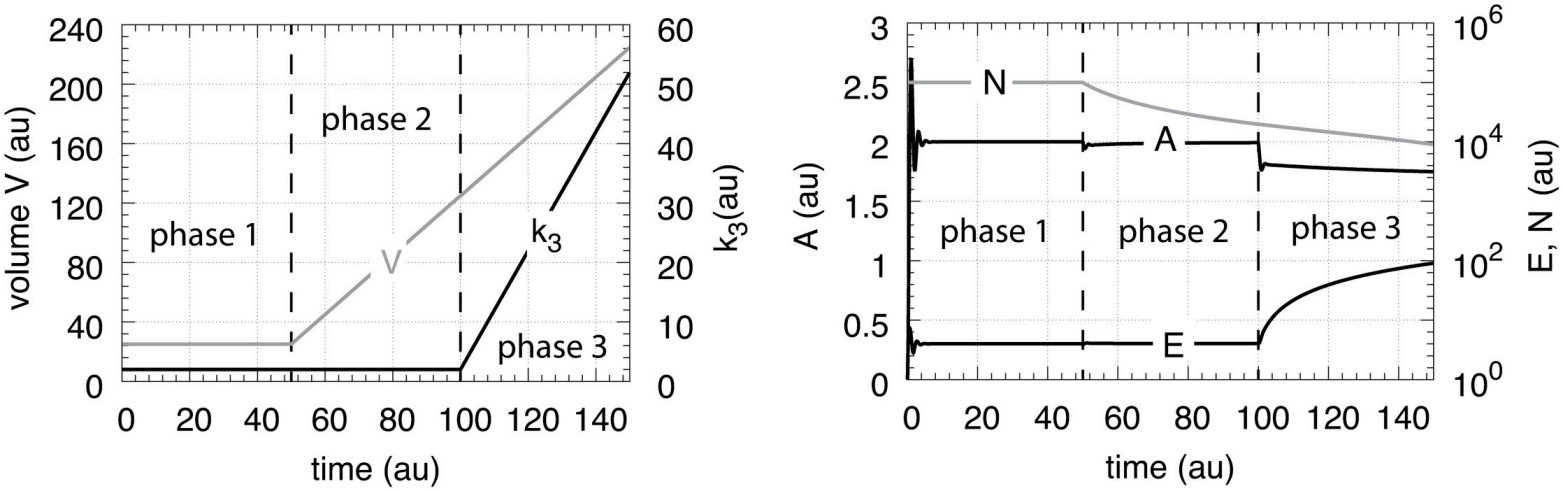
Performance of the motif 1 zero-order controller with internally generated compensatory flux (Fig 17; Eqs 50–54). Phase 1: constant volume *V* and constant *k*_3_. Initial volume, concentrations, and rate constants: *V*_0_ = 25.0, $\dot{V}=0.0$, *A*_0_ = 0.0, *E*_0_ = 0.0, *M*_0_ = 4 × 10^4^, *N*_0_ = 1 × 10^5^, *P*_0_ = 0.0, *k*_2_ = 1.0, *k*_3_ = 2.0, $\dot{k_{3}}=0.0$, *k*_4_ = 20.0, *k*_5_ = 1 × 10^−6^, *k*_6_ = 10.0, *k*_7_ = 1 × 10^−6^, *k*_8_ = 1 × 10^−6^. The controller moves *A* to its set-point at $A_{set}^{theor}=(k_{4}/k_{6})=2.0$ (Eq 55). Phase 2: rate constants remain the same as in phase 1, but *V* increases linearly with $\dot{V}=2.0$, while *k*_3_ remains constant at *k*_3_ = 2.0. The controller is able to keep *A* at $A_{set}^{theor}=(k_{4}/k_{6})=2.0$ in agreement with Eq 55. Phase 3: *V* continues to increase with the same speed while *k*_3_ now linearly increases with $\dot{k_{3}}=1.0$. As indicated by Eq 55 *A*_*ss*_ leads to a constant offset below $A_{set}^{theor}$.

When $\dot{k_{3}}=0$ and $\dot{V}$ = constant *A*_*ss*_ becomes $A_{set}^{theor}=k_{4}/k_{6}$ and the motif 1 zero-order controller is able to compensate for a constant growth rate (Fig 18, phases 1 and 2). However, when *k*_3_ increases linearly, *A*_*ss*_ is below $A_{set}^{theor}$ and remains constant as long as sufficient *M* and *N* are present (Fig 18, phase 3). Thus, in comparison with a transporter-mediated compensatory fluxes, the motif 1 zero-order controller with an internally generated compensatory flux shows an improved performance by being able to compensate for a constant growth rate in the absence of other outflow perturbations in *A*.

### Motif 1 antithetic controller

**Fig 19:**
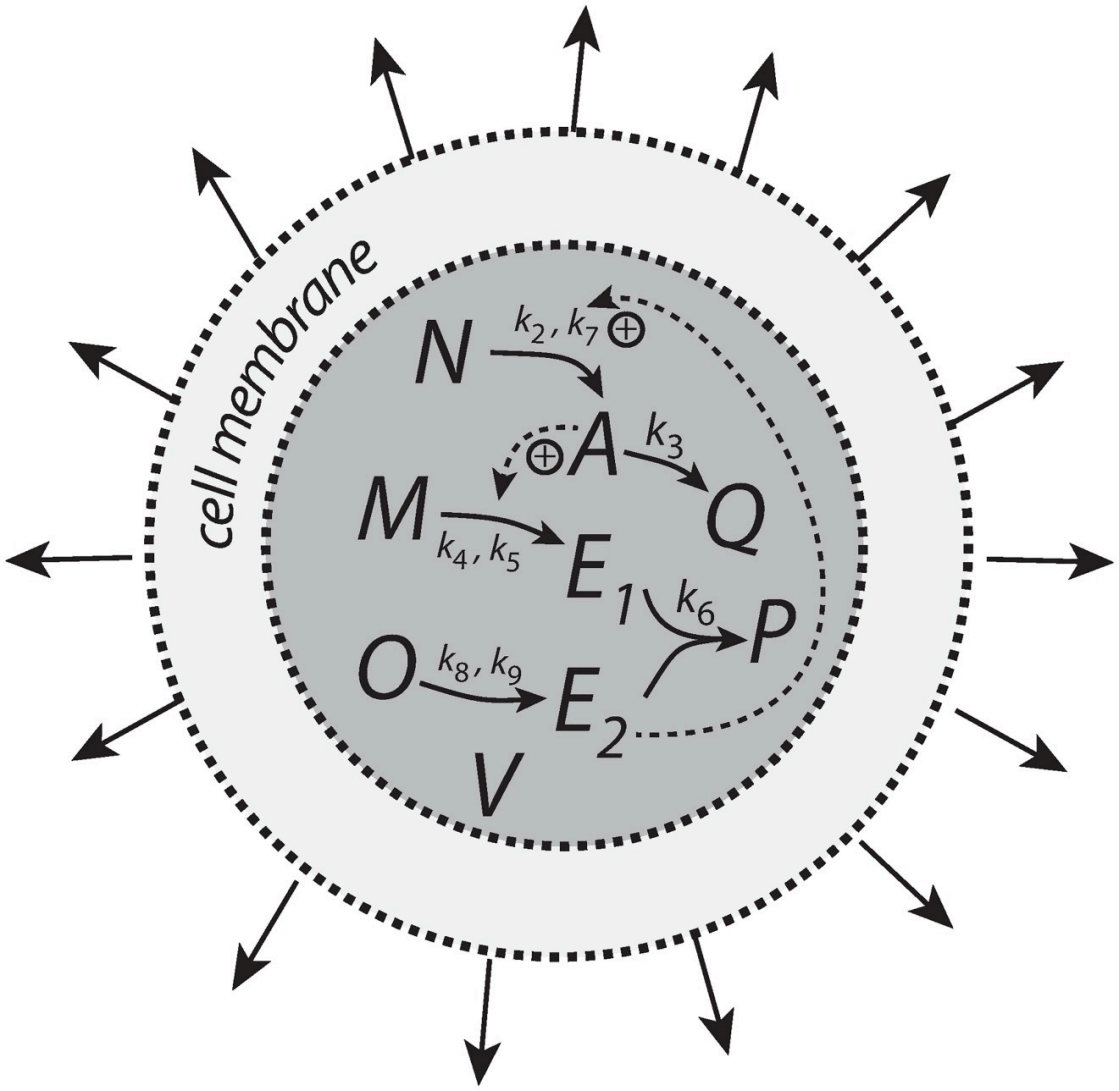
The antithetic controller with an internal generated compensatory flux.

When the antithetic integral controller is equipped with an internally generated compensatory flux (Fig 19) its performance towards constant growth and linearly increasing outflow perturbations *k*_3_ is significantly improved in comparison with a controller having a transporter generated compensatory flux (Fig 9). The rate equation for *A* is now changed to

$$\begin{array}{c} \dot{A}=(\frac{k_{2}\cdot N}{k_{7}+N})E_{2}-k_{3}\cdot A-A(\frac{\dot{V}}{V}) \end{array}$$ (56)

while the other rate equations (Eqs 22–27) remain the same.

When $\dot{V}$ is constant *A*_*ss*_ becomes (S4 Text)

$$\begin{array}{c} A_{ss}=\frac{k_{2}k_{8}}{k_{2}k_{4}+\dot{k_{3}}} \end{array}$$ (57)

**Fig 20:**
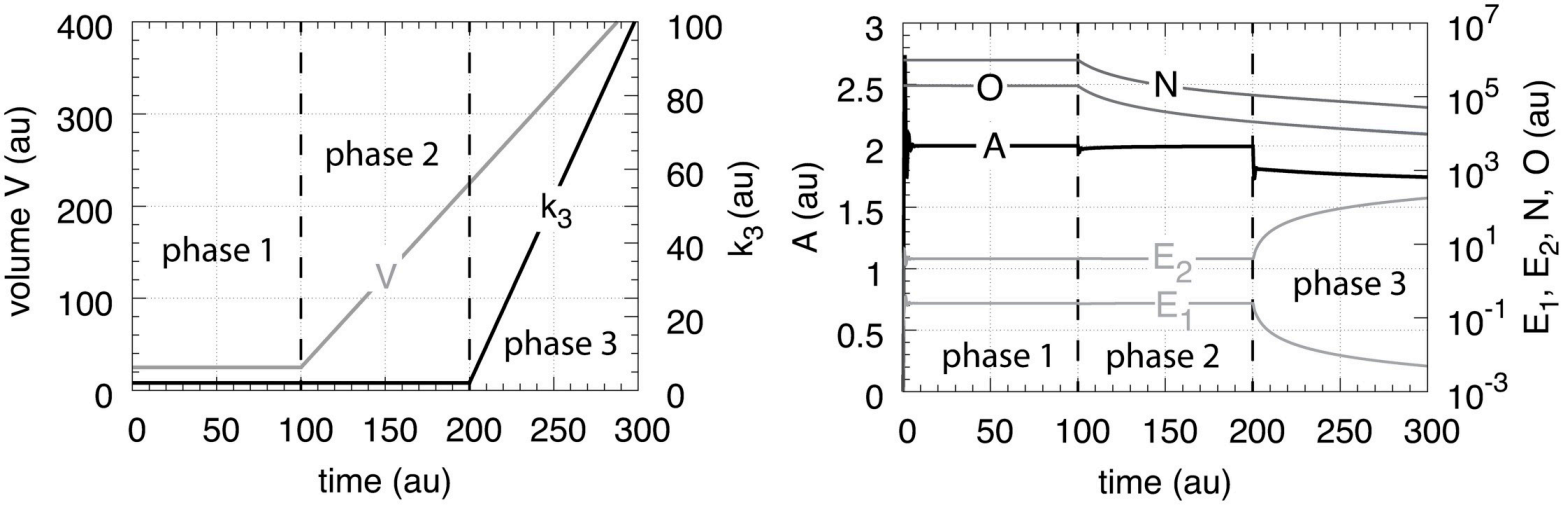
Performance of the antithetic controller when the compensatory flux is homogeneously generated within the cellular volume (Eqs 56 and 22–27). Phase 1: constant volume *V* and constant *k*_3_. Initial concentrations and rate constant values: *V*_0_ = 25.0, $\dot{V}=0.0$, *A*_0_ = 0.0, *E*_1,0_ = 0.0, *E*_2,0_ = 0.0, *M*_0_ = 2 × 10^5^, *N*_0_ = 1 × 10^6^, *O*_0_ = 2 × 10^5^, *k*_2_ = 1.0, *k*_3_ = 2.0, $\dot{k_{3}}=0.0$, *k*_4_ = 10.0, *k*_5_ = 1 × 10^−6^, *k*_6_ = 20.0, *k*_7_ = 1 × 10^−5^, *k*_8_ = 20.0, *k*_9_ = 1 × 10^−5^. The controller moves *A* to $A_{set}^{theor}=(k_{8}/k_{4})=2.0$ (Eq 57 when $\dot{k_{3}}=0$). Phase 2: rate constants remain the same as in phase 1, but *V* increases linearly with $\dot{V}=2.0$, while *k*_3_ remains constant at *k*_3_ = 2.0. The controller is able to maintain *A* at $A_{set}^{theor}=k_{4}/k_{6}=2.0$ in agreement with Eq 57. Phase 3: *V* continues to increase with the same speed while *k*_3_ now linearly increases with $\dot{k_{3}}=1.0$. As indicated by Eq 57 the controller is no longer able to keep *A* at $A_{set}^{theor}$ but shows a constant steady state value below its theoretical set-point.

As indicated by Eq 57 numerical results show (Fig 20, phase 2) that the antithetic controller is now able to compensate for linear volume increases by moving *A* to $A_{set}^{theor}=(k_{8}/k_{4})$. However, an offset in *A*_*ss*_ below $A_{set}^{theor}$ is observed when, in addition, *k*_3_ increases linearly with time, i.e., when $\dot{k_{3}}$ is constant.

Although not explicitly shown here, during the volume increase, the mass (mole) balances described by Eqs 30 and 31 are obeyed in addition to the mass balance connecting *N*, *A*, and *Q*

$$\begin{array}{c} n_{N,0}=n_{N}\left(t\right)+n_{A}\left(t\right)+n_{Q}\left(t\right) \end{array}$$ (58)

where *n*_*N*,0_ is the number of moles of initial *N* at *t* = 0 with *n*_*A*,0_ = *n*_*Q*,0_ = 0.

### Motif 1 autocatalytic controller

**Fig 21:**
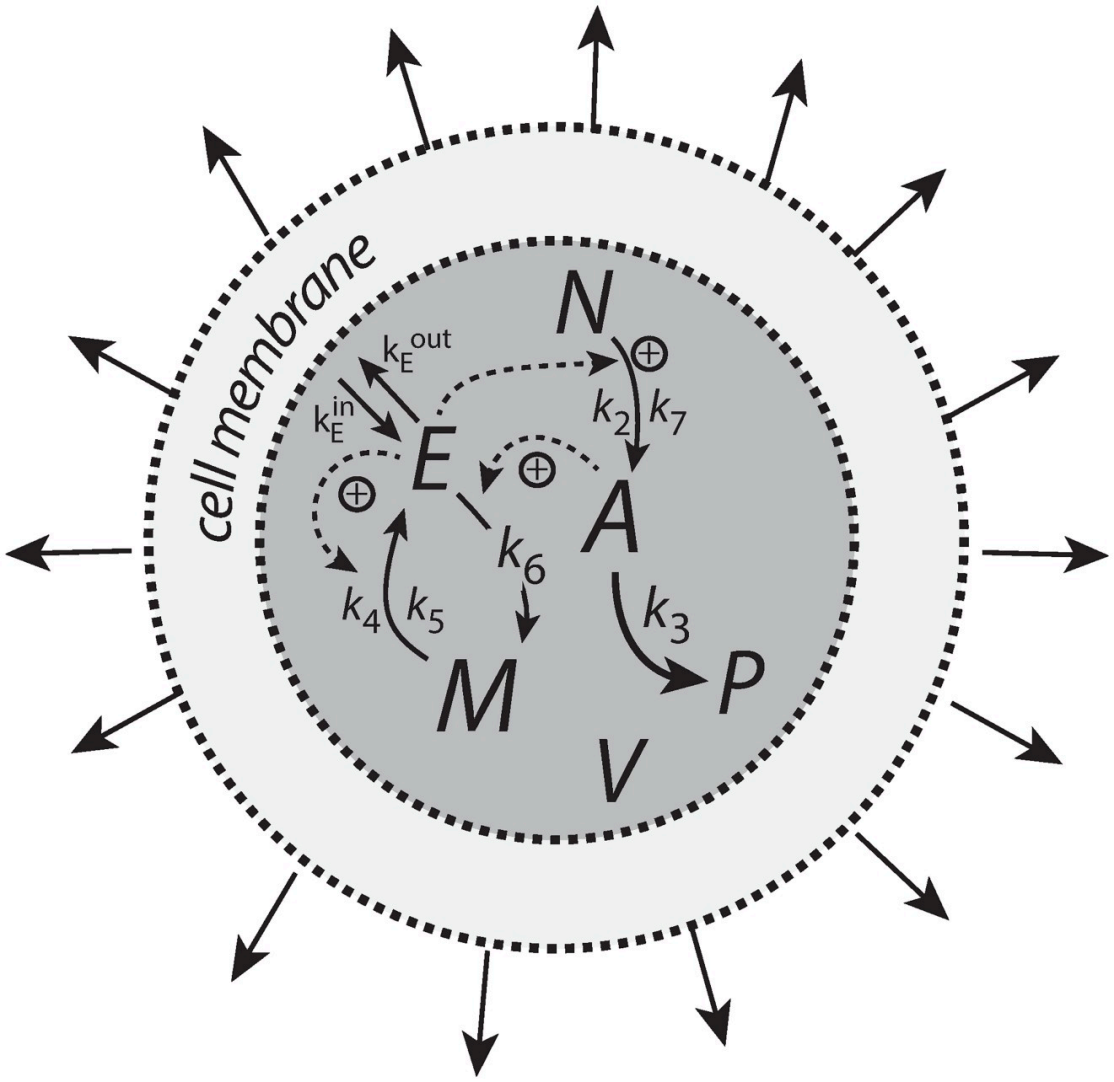
Scheme of autocatalytic controller with an internally generated compensatory flux from compound *N*. Otherwise the controller has the same structure as shown in Fig 10.

Fig 21 shows the autocatalytic controller but now with an internally generated compensatory flux. As for the motif 1 zero-order controller (Fig 17) the compensatory flux originates from compound *N* and is activated by *E*. *N* is present in high concentration and forms *A* by a zero-order process with respect to *N*.

The rate equation for the controlled variable *A* is

$$\begin{array}{c} \dot{A}=k_{2}\cdot E(\frac{N}{k_{7}+N})-k_{3}\cdot A-A(\frac{\dot{V}}{V}) \end{array}$$ (59)

while the rate equations for *E* and *M* remain the same as Eqs 34 and 35. Species *P* is included with the rate equation

$$\begin{array}{c} \dot{P}=k_{3}\cdot A-P(\frac{\dot{V}}{V}) \end{array}$$ (60)

to test that the mass (mole) balance between *N*, *A*, and *P* is preserved.

**Fig 22:**
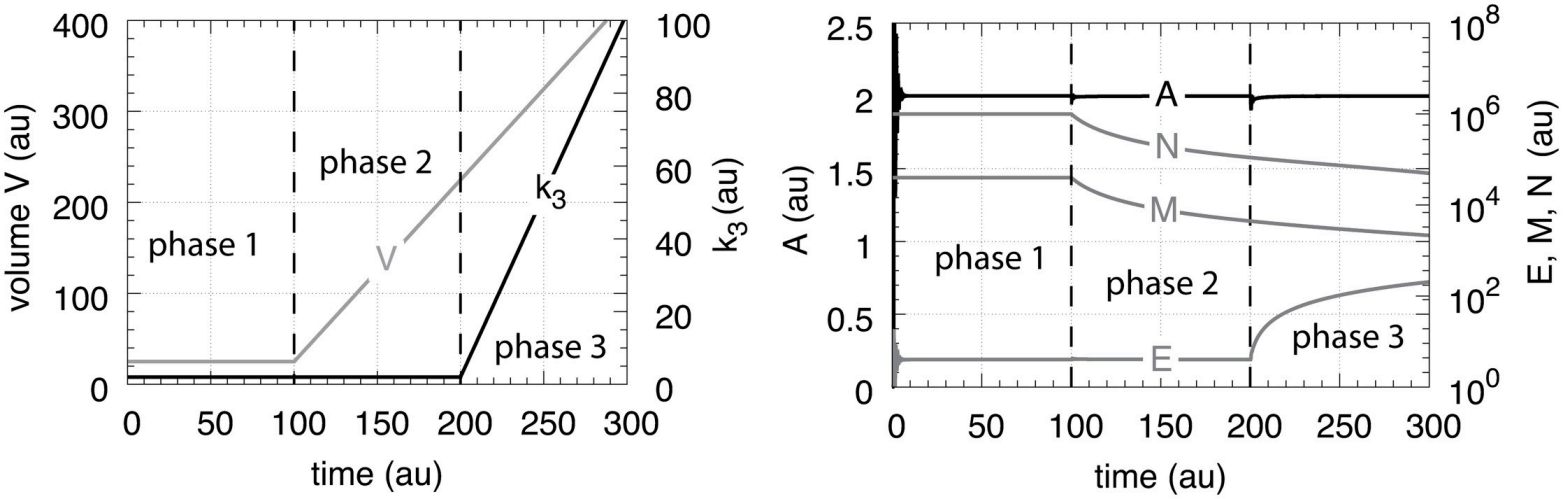
Performance of the autocatalytic controller when the compensatory flux is generated within the cellular volume (Eqs 34, 35, 59 and 60). Phase 1: constant volume *V* and constant *k*_3_. Initial concentrations and rate constant values: *V*_0_ = 25.0, $\dot{V}=0.0$, *A*_0_ = 0.0, *E*_0_ = 0.0, *M*_0_ = 4 × 10^4^, *N*_0_ = 1 × 10^6^, *k*_2_ = 1.0, *k*_3_ = 2.0, $\dot{k_{3}}=0.0$, *k*_4_ = 20.0, *k*_5_ = 1 × 10^−6^, *k*_6_ = 10.0, *k*_7_ = 1 × 10^−6^, $k_{E}^{in}=k_{E}^{out}=1\times 10^{-5}$. The controller moves *A* to its theoretical set-point at $A_{set}^{theor}=(k_{4}/k_{6})=2.0$ (Eq 37). Phase 2: rate constants remain the same as in phase 1, but *V* increases linearly with $\dot{V}=2.0$, while *k*_3_ remains constant at *k*_3_ = 2.0. The controller is able to maintain *A* at $A_{set}^{theor}$ in agreement with Eq 37. Phase 3: *V* continues to increase with the same speed while *k*_3_ now linearly increases with $\dot{k_{3}}=1.0$. As indicated by Eq 38 the controller keeps *A* at $A_{set}^{theor}$ as *k*_3_ increases.

The controller’s steady state in *A* is also in this case described by Eq 38 (S5 Text). In contrast to the other controllers, even when $\dot{V}$ and $\dot{k_{3}}$ are constant, the autocatalytic controller is able to move *A* to $A_{set}^{theor}=(k_{4}/k_{6})$ (Fig 22).

When $k_{E}^{in}$ and $k_{E}^{out}$ are large and cannot be neglected the steady state in *A* is described by the quadratic equation (S5 Text)

$$\begin{array}{c} A_{ss}^{2}-A_{ss}(\frac{k_{4}-k_{E}^{out}}{k_{6}}-\frac{\dot{k_{3}}}{k_{3}k_{6}})-\frac{k_{2}k_{E}^{in}}{k_{3}k_{6}}=0 \end{array}$$ (61)

In case only *V* increases linearly *A*_*ss*_ is given by the solution of Eq 61, independent of *V*’s growth rate. On the other hand, if *k*_3_ increases linearly, the terms $\dot{k_{3}}/k_{3}k_{6}$ and $k_{2}k_{E}^{in}/k_{3}k_{5}$ go to zero for large *k*_3_ and *A*_*ss*_ is given by $\left(k_{4}-k_{E}^{out}\right)/k_{6}$ as described by Eq 42 for the transporter-based compensatory flux.

### Motif 2 zero-order controller

**Fig 23:**
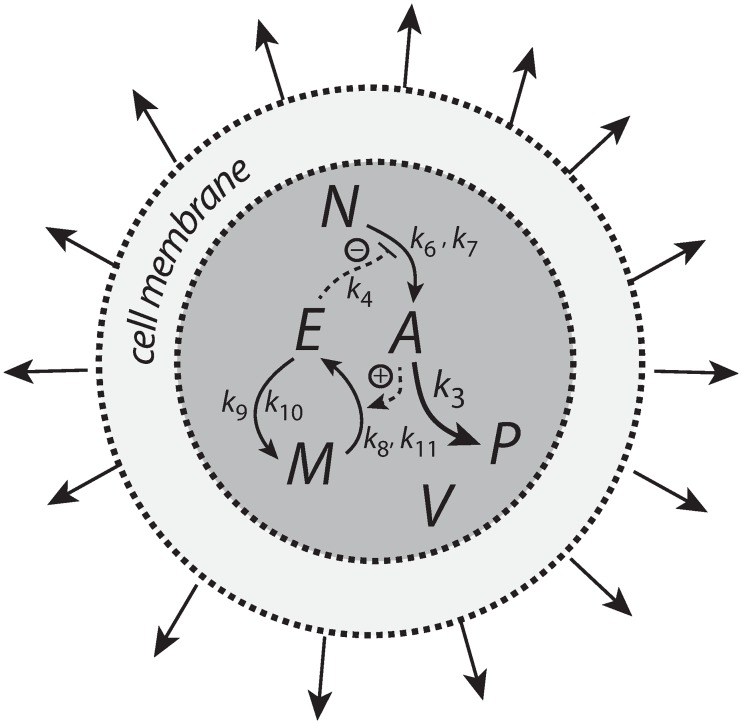
Motif 2 type controller with integral control based on zero-order kinetics and a cell-internally generated compensatory flux from compound *N*.

The rate equations for the motif 2 controller using a cell-internal compensatory flux are (Fig 23):

$$\begin{array}{c} \dot{A}=(\frac{k_{4}\cdot k_{6}}{k_{4}+E})\cdot (\frac{N}{k_{7}+N})-k_{3}\cdot A-A(\frac{\dot{V}}{V}) \end{array}$$ (62)

$$\begin{array}{c} \dot{E}=(\frac{k_{8}\cdot M}{k_{11}+M})\cdot A-\frac{k_{9}\cdot E}{k_{10}+E}-E(\frac{\dot{V}}{V}) \end{array}$$ (63)

$$\begin{array}{c} \dot{M}=-(\frac{k_{8}\cdot M}{k_{11}+M})\cdot A+\frac{k_{9}\cdot E}{k_{10}+E}-M(\frac{\dot{V}}{V}) \end{array}$$ (64)

$$\begin{array}{c} \dot{N}=-(\frac{k_{4}\cdot k_{6}}{k_{4}+E})\cdot (\frac{N}{k_{7}+N})-N(\frac{\dot{V}}{V}) \end{array}$$ (65)

$$\begin{array}{c} \dot{P}=k_{3}\cdot A-P(\frac{\dot{V}}{V}) \end{array}$$ (66)

**Fig 24:**
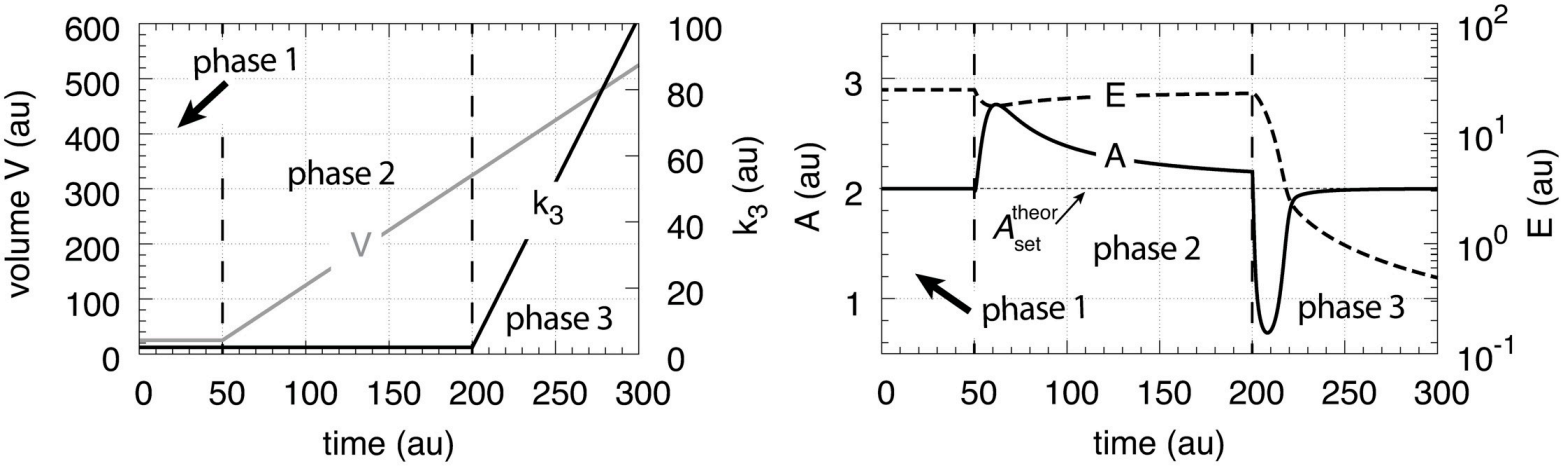
Performance of the motif 2 feedback scheme with zero-order based integral control and a cell-internal compensatory flux. Rate constants and initial conditions: *k*_3_ = 2.0, *k*_4_ = 1 × 10^−3^, *k*_6_ = 1 × 10^5^, *k*_7_ = 1 × 10^−6^, *k*_8_ = 1.0, *k*_9_ = 2.0, *k*_10_ = *k*_11_ = 1 × 10^−6^, *A*_0_ = 2.0, *E*_0_ = *V*_0_ = 25.0, *M*_0_ = 1 × 10^6^, *N*_0_ = 3 × 10^6^. Phase 1: *V* and *k*_3_ remain unchanged. Phase 2: *V* increases linearly with $\dot{V}=2.0$, while *k*_3_ remains constant. Phase 3: *V* continues to increase and *k*_3_ increases linearly with $\dot{k_{3}}=1.0$.

Fig 24 shows the performance of the motif 2 feedback structure with zero-order integral control. The controller is able to defend successfully $A_{set}^{theor}$ against a linear increase in *V* (phase 2) as well as against linear increase in *V* and a simultaneous linear increase in *k*_3_ (phase 3). For both cases the controller will move *A* precisely to $A_{set}^{theor}=k_{9}/k_{8}$ without any offset (see S6 Text for details).

## Case B.2: Controllers with cell-internal compensatory fluxes and exponential time-dependent perturbations

The controllers are exposed to the same exponential perturbation profiles as in Fig 14. The exponential growth of *V* is written as $\dot{V}=\kappa \cdot V$, where *κ* (>0) is a constant and related to the doubling time of *V* given by ln 2/*κ*.

**Fig 25:**
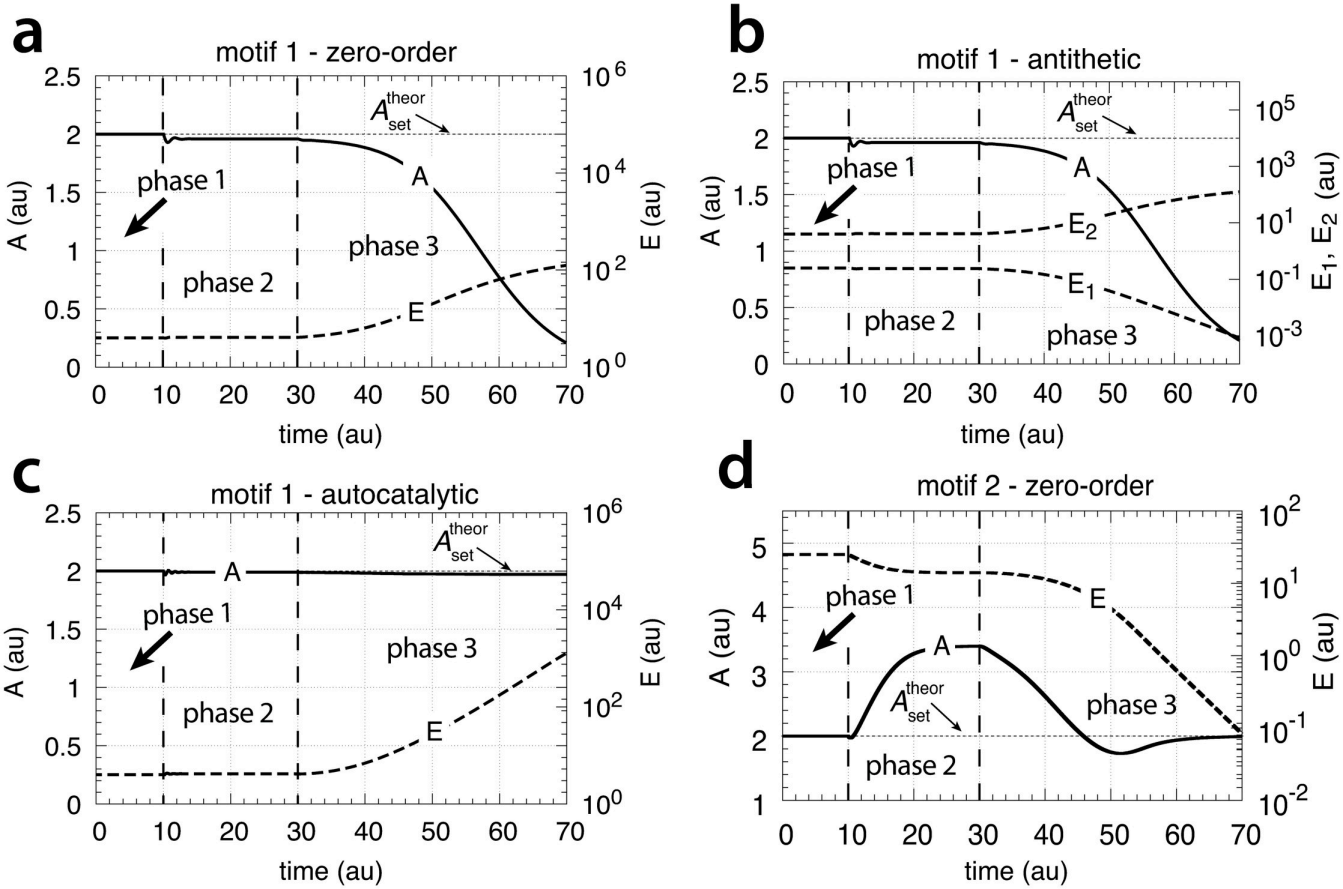
Behaviors of the motif 1 zero-order, antithetic, autocatalytic and motif 2 zero-order controllers with internal compensatory fluxes in response to an exponential increase in *V* and *k*_3_. Time/perturbation profiles of *V* and *k*_3_ are the same as in Fig 14. (a) Behavior of the motif 1 zero-order controller. Rate constant values as in Fig 18. Initial concentrations: *A*_0_ = 2.0, *E*_0_ = 4.0, *V*_0_ = 25.0, *M*_0_ = 4 × 10^9^, *N*_0_ = 1 × 10^6^. (b) Behavior of the antithetic controller. Rate constants as in Fig 20. Initial concentrations: *A*_0_ = 2.0, *E*_1,0_ = 0.25, *E*_2,0_ = 4.0, *V*_0_ = 25.0, *M*_0_ = *N*_0_ = *O*_0_ = 1 × 10^6^, *Q*_0_ = *P*_0_ = 0.0. During phase 2 the controller shows a slight but constant offset below $A_{set}^{theor}$. During phase 3 the controller breaks down when both *V* and *k*_3_ increase exponentially. (c) Behavior of the autocatalytic controller. Rate constants are as described in Fig 22. Initial concentrations: *A*_0_ = 2.0, *E*_0_ = 4.0, *V*_0_ = 25.0, *M*_0_ = 4 × 10^9^, *N*_0_ = 1 × 10^7^. During autocatalytic growth only (phase 2) the autocatalytic controller is able to move *A*_*ss*_ precisely to $A_{set}^{theor}$, but shows an offset from $A_{set}^{theor}$ when both *k*_3_ and *V* increase exponentially). (d) Behavior of the motif 2 based controller (Eqs 62–66). Rate constants and initial conditions as in Fig 24. Note the significant overcompensation (offset above $A_{set}^{theor}$) during phase 2, but the return to $A_{set}^{theor}$ (=*k*_9_/*k*_8_) when *k*_3_ starts to grow exponentially.

Fig 25a shows the performance of the motif 1 zero-order controller while Fig 25b shows the responses of the motif 1 antithetic controller. During exponential growth and constant *k*_3_ the motif 1 zero-order and the antithetic controller show slight offsets from the theoretical set-point $A_{set}^{theor}$, while during phase 3 when both *V* and *k*_3_ increase exponentially, both controllers break down. Besides their different kinetic implementation of integral control both the motif 1 zero-order and the motif 1 antithetic controller have analogous responses (for details, see S3 and S4 Texts).

Fig 25c shows the response of the autocatalytic controller when $k_{E}^{in}=k_{E}^{out}=1\times 10^{-5}$. The controller is able to keep *A* at $A_{set}^{theor}$ during exponential growth while *k*_3_ is kept constant. Only when *V* and *k*_3_ both increase exponentially then there is an offset from $A_{set}^{theor}$, which can be estimated as:

$$\begin{array}{c} A_{ss}=\frac{k_{4}}{k_{6}}-\frac{\kappa }{k_{6}}-\frac{\zeta }{k_{6}} \end{array}$$ (67)

where the theoretical set-point $A_{set}^{theor}=k_{4}/k_{6}$ and *κ* and *ζ* describe the doubling times ln 2/*κ* and ln 2/*ζ* of the exponential increases for *V* and *k*_3_, respectively (see S5 Text).

In case $k_{E}^{in}$ and $k_{E}^{out}$ are large Eq 67 changes to (S5 Text):

$$\begin{array}{c} A_{ss}=\frac{k_{4}}{k_{6}}-\frac{\kappa }{k_{6}}-\frac{\zeta }{k_{6}}-\frac{k_{E}^{out}}{k_{6}} \end{array}$$ (68)

The motif 2 based controller shows in phase 2 a significant overcompensation from $A_{set}^{theor}$ when exposed to exponential growth only. The overcompensated steady state in *A* at constant *k*_3_ and exponential growth can be expressed as

$$\begin{array}{c} A_{ss}=A_{set}^{theor}+\frac{\kappa }{k_{8}}E_{ss} \end{array}$$ (69)

where $A_{set}^{theor}=k_{9}/k_{8}$ and (*κ*/*k*_8_)*E*_*ss*_ is the overcompensated offset (S6 Text).

The response kinetics of the motif 2 based controller is mostly determined by *k*_4_, which reflects the derepression property by *E*. For large *k*_4_ the derepression by *E* is observed to be slow and less effective.

Remarkably, when both *k*_3_ and *V* increase exponentially in phase 3 the controller is able to move *A* close to $A_{set}^{theor}$. For this case *A*_*ss*_ can be written as (S6 Text)

$$\begin{array}{c} A_{ss}=(\frac{\gamma_{0}}{1+\gamma_{0}})A_{set}^{app} \end{array}$$ (70)

where

$$\begin{array}{c} \gamma_{0}=\frac{k_{4}k_{6}k_{8}}{\dot{k_{3}}{\left(k_{4}+E\left(t\right)\right)}^{2}} \end{array}$$ (71)

and

$$\begin{array}{c} A_{set}^{app}=A_{set}^{theor}+\frac{\kappa }{k_{8}}E\left(t\right) \end{array}$$ (72)

Note that during phase 3 *E* is not in a steady state, but decreases due to the controller’s derepression, while $\dot{k_{3}}$ increases exponentially. However, the derepression kinetics by *E* are faster than the exponential increase of $\dot{k_{3}}$ (Eq 71), such that *γ*_0_ increases and $A_{set}^{app}$ and *A*_*ss*_ approach $A_{set}^{theor}$ (S6 Text).

### Growth related to the surface to volume ratio and controllers with cell-internal compensatory fluxes

**Fig 26:**
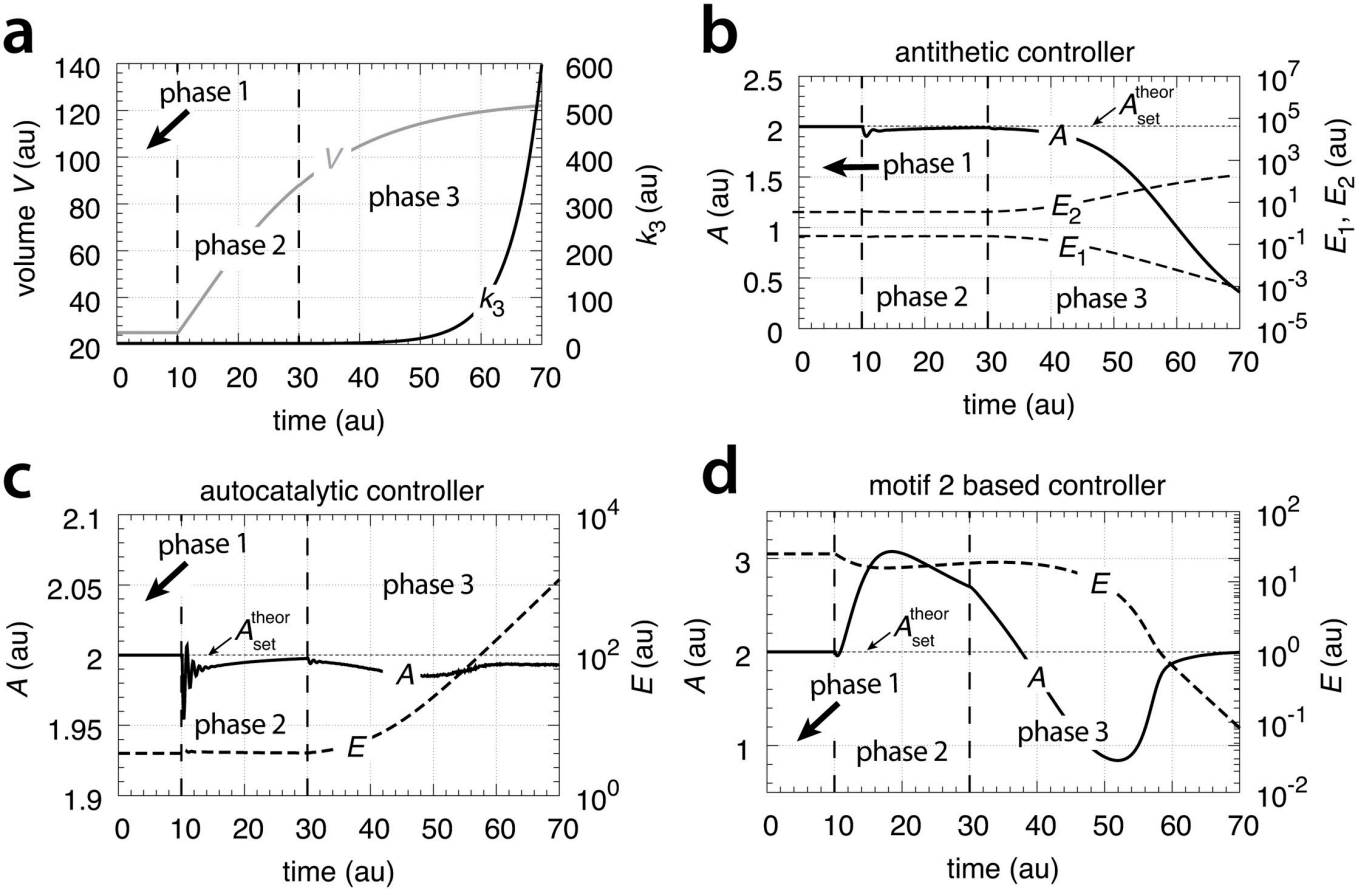
Performance of the antithetic, autocatalytic and motif 2 based controllers towards surface/volume related growth in *V* and exponentially increasing outflow perturbation *k*_3_ with cell-internal compensatory flux. Rate constant values and initial conditions as in Fig 25.

Here we show how the four controllers having cell internal compensatory fluxes perform with respect to a surface to volume ratio related growth law as found for spherical bacteria ([9, 10, 13], Eq 2). We consider again three phases as in the previous sections, but with the difference that *V* now grows according to Eq 2 with *η* = 1 and *ξ* = 0.2 (Fig 26a). The values of *η* and *ξ* are arbitrarily chosen. The outflow perturbation, described by *k*_3_, is kept constant during phases 1 and 2, but increases exponentially during phase 3 (Eq 49). The response behaviors of the controllers towards increasing volume (when *k*_3_ is kept constant) is initially very similar to that when *V* increases linearly. However, the motifs gain more and more control as $\dot{V}$ decreases, provided that there is sufficient material in the cell to generate enough *E*’s (for the motif 1 controllers) or that there is still sufficient *E* left (for the motif 2 controller) to keep the negative feedback loop operating.

As an example, Fig 26 shows the behavior of the motif 1 antithetic and autocatalytic controllers and the motif 2 zero-order controller when *k*_3_ in phase 3 increases exponentially as described by Fig 14 and compensatory fluxes are generated cell internally. The motif 1 zero-order controller’s behavior (not shown) is again very similar in comparison with the motif 1 antithetic controller.

## Overview of results

Tables 1 and 2 gives an overview of controller performances by dividing perturbations into (i) linear *V* only, (ii) linear *V* and *k*_3_, (iii) exponential *V* only, and (iv) exponential *V* and *k*_3_. Controller performances are described by the four categories *perfect adaptation*, *partial adaptation*, *over-adaptation*, and *breakdown*. Perfect adaptation means that the controller is able to keep *A* at $A_{set}^{theor}$. A controller with partial adaptation can maintain a constant *A* value during an applied outflow perturbation, but below $A_{set}^{theor}$. A controller showing over-adaptation keeps *A* above $A_{set}^{theor}$ even when the perturbation leads to a decrease in *A*. Controller breakdown means that the controller is unable to withstand the perturbation and *A* goes to zero.

**Table 1:** Performance of controllers based on internal generated compensatory fluxes.

controller type	linear *V* only	linear *V* and *k*_3_	exponential *V* only	exponential *V* and *k*_3_
**m1—zero-order**	perfect adaptation	partial adaptation	breakdown	breakdown
**m1—antithetic**	perfect adaptation	partial adaptation	breakdown	breakdown
**m1—autocatalytic**	perfect adaptation	perfect adaptation	perfect adaptation	partial adaptation
**m2—zero-order**	perfect adaptation	perfect adaptation	over-adaptation	perfect adaptation

**Table 2:** Performance of controllers based on transporter based compensatory fluxes.

controller type	linear *V* only	linear *V* and *k*_3_	exponential *V* only	exponential *V* and *k*_3_
**m1—zero-order**	partial adaptation	breakdown	breakdown	breakdown
**m1—antithetic**	partial adaptation	breakdown	breakdown	breakdown
**m1—autocatalytic**	perfect adaptation	perfect adaptation	partial adaptation	partial adaptation
**m2—zero-order**	perfect adaptation	perfect adaptation	perfect adaptation	perfect adaptation

Concerning the results with respect to surface/volume related growth we group this growth law together with the category of linear growth, because controllers behave initially quite similar towards these two growth laws (compare phases 2 in Fig 16b–16d with respective phase 2 behaviors of Figs 9, 11 and 13 and phases 2 in Fig 26b–26d with the phase 2 behaviors of Figs 20, 22 and 24, respectively).

Clearly, motif 1 controllers based on zero-order or on antithetic integral control, cannot oppose an exponential volume increase or when an additional exponential increase in *k*_3_ is applied. When exponentially increasing perturbations are applied the motif 1 autocatalytic controller shows good performances with a stable offset in *A* below $A_{set}^{theor}$, but requires the presence of sufficient controller species *E* to maintain autocatalysis. The motif 2 controller using zero-order based integral control shows best performance, and is able to keep *A* at $A_{set}^{theor}$, even when both *V* and *k*_3_ increase exponentially. However, the drawback of controllers based on derepression, like motif 2, is that controller breakdown occurs when concentrations of the derepressing control species is getting too low.

## Discussion

### Internal model principle and the kinetic limit of controllers

From Tables 1 and 2 it is seen that the motif 2 controller outperforms the other controllers. The derepression kinetics of the motif 2 controller is described by the term (Eqs 43 and 62):

$$\begin{array}{c} f_{inhib}\left(E\right)=\frac{k_{4}}{k_{4}+E} \end{array}$$ (73)

which is an essential part in generating the compensatory flux in *A*. For decreasing (derepressing) *E* *f*_*inhib*_(*E*) increases with hyperbolic response kinetics, i.e. having exponentially increasing doubling times. The motif 2 controller is therefore able, as observed [18], to counteract hyperbolically decreasing concentrations in *A*. The balancing between a perturbation and the by the perturbation induced compensatory flux reflects the *Internal Model Principle* [25–27], which states that if a controller is able to oppose a perturbation, then the controller has the capability to generate that kind of perturbation internally. For the motif 2 controller the hyperbolic response kinetics represents the upper kinetic limit which the controller can handle. In addition, the motif 2 controller will handle any perturbing rate laws with doubling times lower than an exponential (constant doubling times relate to an exponential rate law), although overcompensation may occur as seen in Fig 25d.

Thus, as indicated in Tables 1 and 2 controllers group according to their kinetic limits, where the motif 2 controller with hyperbolic response kinetics performs better than controllers based on exponential/autocatalytic or linear responses.

Repression/derepression kinetics are ubiquitously used in homeostatic mechanisms (see Supplementary Material in [7]), in gene on/off regulations [28–30] and as rhythm generators [31, 32]. The fast response of derepression is also used in signaling [33], but may be needed to be kept under additional control as indicated in a study of the nitrogenase switch [30] to avoid overenhanced/overcompensated responses.

Breakdown of the motif 2 controller occurs when the compensatory flux has reached its maximum value (described by rate constant *k*_2_ in Figs 12 and 23).

A somewhat surprising behavior of the motif 2 controller is its overcompensation when growth increases exponentially at constant *k*_3_ (see phase 2 in Fig 25d). The overcompensation can be described analytically (Eq 69). Its origin is due to the fact that with a cell-internal compensatory flux an exponetial increase in *V* at constant *k*_3_ allows for steady states in *A* and *E*, independent of *V*, where *A*_*ss*_ is *larger* than $A_{set}^{theor}$ (S6 Text).

### Performance improvement by increased controller aggressiveness

Although the motif 1 zero-order and the antithetic controllers break down when exposed to exponential growth and perturbations (Figs 15a and 15b and 25a and 25b), their performance can be significantly improved at constant $\dot{V}$ by increasing of what can be described as the controllers’ aggressiveness. By aggressiveness of a controller we mean loosely the controller’s response to a perturbation in terms of (mainly) quickness and precision. Increasing the aggressiveness of a controller will generally lead to a quicker controller response and an improved controller precision.

The aggressiveness of an integral controller can be varied by the controller’s gain. The gain is a factor in front of the error integral. For an ideal motif 1 zero-order integral controller (working at constant *V* and *k*_3_) $\dot{E}$ is proportional to the error $e=(A_{set}^{theor}-A)$ [7], i.e.,

$$\begin{array}{c} \dot{E}=k_{6}(\frac{k_{4}}{k_{6}}-A) \end{array}$$ (74)

where *k*_6_ is the controller gain and *k*_4_/*k*_6_ is the controller’s theoretical set-point, $A_{set}^{theor}$. As indicated by Eq 74 the concentration of *E* is proportional to the integrated error with respect to time. By increasing *k*_6_ and *k*_4_ such that $A_{set}^{theor}$ remains unchanged the gain of the controller is increased and the controller becomes more aggressive.

For constant $\dot{V}$ and *k*_3_ the steady state of *A* for the motif 1 zero-order controller is given by Eq 20

$$\begin{array}{c} A_{ss}=\frac{k_{4}}{k_{6}+\frac{2\dot{V}k_{3}}{k_{2}}} \end{array}$$ (20)

where the offset in *A*_*ss*_ below $A_{set}^{theor}$ is due to the term $2\dot{V}k_{3}/k_{2}$. This term indicates that for increasing $\dot{V}$ and/or increasing *k*_3_ values the controller will break down and *A* will go to zero as observed in Fig 15a. There are two ways the controller’s aggressiveness can be increased. One way, as indicated above, is by increasing *k*_4_ and *k*_6_ such that $A_{set}^{theor}=k_{4}/k_{6}$ is preserved with *k*_6_ becoming much larger than $2\dot{V}k_{3}/k_{2}$. As a result the controller’s response kinetics become quicker and *A*_*ss*_ moves closer to $A_{set}^{theor}=k_{4}/k_{6}$. The other way is to increase *k*_2_, which means to increase the activity of the transporter. In a synthetic biology context this could be done by over-expressing the genes which code for the transporter. On the other hand, “normal” cells may already have optimized controller aggressiveness or may change it in response to environmental conditions.

Similar arguments apply also for the antithetic controller. Qian et al. [34] have shown that when the controller dynamics become faster than growth this leads to an improved controller performance.

**Fig 27:**
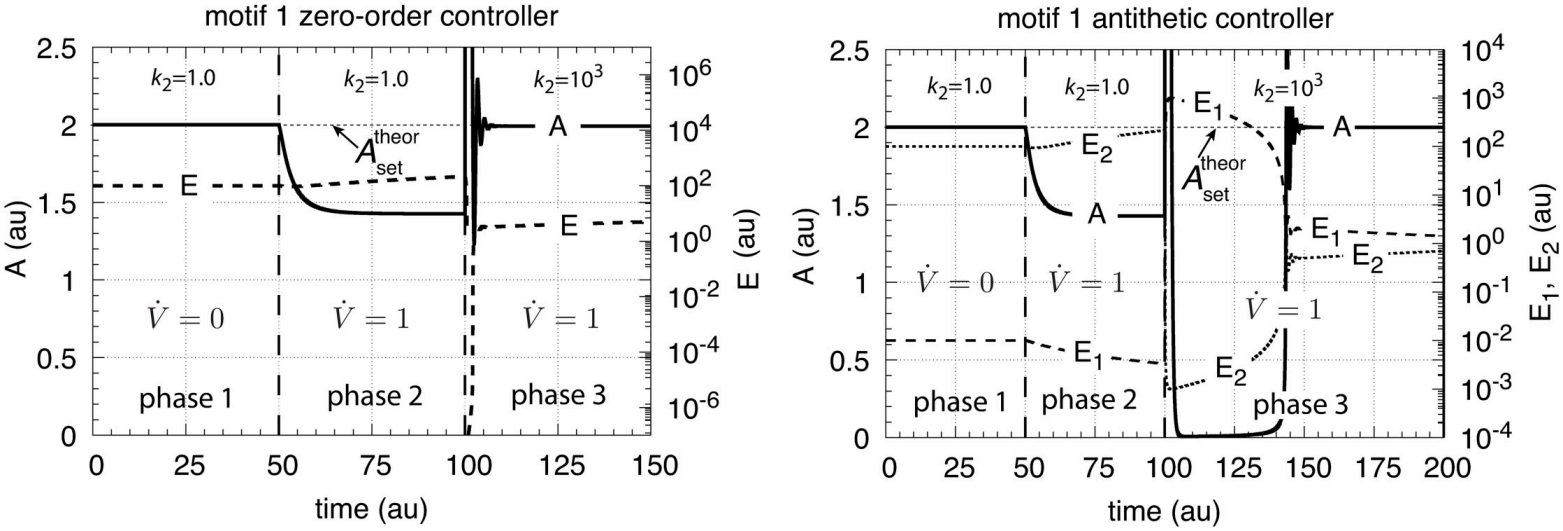
Increased transporter activity (*k*_2_ values) lead to increased aggressiveness and improved controller precision for transporter-based motif 1 zero-order controller (left panel) and motif 1 antithetic controller (right panel) during constant growth (see Figs 6 and 8). Phase 1: controllers are at their steady state, no growth, *k*_2_ = 1.0. Phase 2: constant growth ($\dot{V}=1.0$) and *k*_2_ = 1.0. Both controllers show an offset in *A*_*ss*_ below $A_{set}^{theor}$. Phase 3: constant growth continues but *k*_2_ is increased to 1 × 10^3^. Both controllers show improved precision and have their *A*_*ss*_ close to $A_{set}^{theor}$, but show different adaptation kinetics during the transition from phase 2 to phase 3. Rate parameters and initial concentrations, zero-order controller: *k*_3_ = 2.0, *k*_4_ = 20.0, *k*_5_ = 1 × 10^−6^, *k*_6_ = 10.0, *k*_7_ = 1 × 10^−6^, *A*_0_ = 2.0, *E*_0_ = 100.0, *V*_0_ = 25.0, *M*_0_ = 1 × 10^7^. Rate parameters and initial concentrations, antithetic controller: *k*_3_ = 2.0, *k*_4_ = 10.0, *k*_5_ = 1 × 10^−6^, *k*_6_ = 10.0, *k*_8_ = 20.0, *k*_9_ = 1 × 10^−6^, *A*_0_ = 2.0, *E*_1,0_ = 1 × 10^−2^, *E*_2,0_ = 1 × 10^2^, *V*_0_ = 25.0, *M*_0_ = *O*_0_ = 1 × 10^8^.

Fig 27 shows the results of increasing the aggressiveness of the motif 1 zero-order and antithetic controllers by increasing *k*_2_ from 1.0 to 1×10^3^. The perturbation is divided into three phases. During the first phase the volume *V* is kept constant at 25.0 and the controllers are at their set-points. In phase 2 the volume increases with a constant rate ($\dot{V}=1.0$). Finally, in phase 3 *V* continues to grow with $\dot{V}=1.0$ but *k*_2_ is increased to 1×10^3^. Both controllers show improved precisions, but show different kinetics in their way to reach $A_{set}^{theor}$.

Similar is the situation when the compensatory flux is internally generated. Eq 55 shows the steady state in *A* for the motif 1 zero-order controller. Also here increasing *k*_2_ values will move *A*_*ss*_ towards the theoretical set-point $A_{set}^{theor}=k_{4}/k_{6}$.

Aggressiveness can also be increased for the autocatalytic controller by increasing *k*_4_ and *k*_6_ such that the *k*_4_/*k*_6_ ratio is maintained. This will move the steady state in *A* closer to its theoretical set-point as offsets become smaller (Eqs S14 and S20 in S5 Text).

### Roles of kinetic implementations of integral control and negative feedback structures

For the transporter-based cases the increased aggressiveness of the motif 1 zero-order and antithetic controllers allows them to defend their theoretical set-points as long as (see Eqs 20 and 29)

$$\begin{array}{c} k_{6}\;\left(\text{or}\;{\text{k}}_{4}\right)\gg 2\dot{V}(\frac{k_{3}}{k_{2}}) \end{array}$$ (75)

However, for exponentially increasing *V* and $\dot{V}$ this can be achieved only for a certain (often short) time period. The motif 1 zero-order controller will break down when Eq 75 is no longer fulfilled. On the other hand, as shown above, the autocatalytic motif 1 controller is able to maintain a stable steady state in *A*, although with an offset from $A_{set}^{theor}$, when *V* and *k*_3_ increase exponentially (but also here dependent on the controller’s aggressiveness). As Eq 38 (for the transporter-based compensatory flux) indicates, any time-dependent perturbation of the type *k*_3_(*t*) = *k*_3,0_ + *a*⋅*t*^*n*^ (*a*, *n* > 0) will be successfully defended by the autocatalytic controller, because $\dot{k_{3}}/k_{3}\to 0$ (S5 Text) and thereby restoring the controller’s theoretical set-point. However, breakdown may occur if no sufficient supply of *E* (for example via *M*, Fig 11) can be maintained.

The sudden breakdown of a fully adapted controller due to capacity limits or exhaustion of the controller variables *E* or *E*_1_/*E*_2_ has an interesting analogy in physiology described by Selye’s *General Adaptation Syndrome* (GAS) [35]. When an animal is exposed to constant but severe stress (for example cold temperatures) the animal can stay, after an alarm reaction, in a stage of resistance, where the animal appears perfectly adapted to its environment. However, after a certain time there appears the stage of exhaustion and the animal dies, despite of sufficient food supplies. To understand the sudden and unexpected breakdown of adaptation, Selye introduced the concept of *adaptation energy* [36]. In our analogous examples here, the adaptation energy can be associated with the amounts of precursors *M*, *N*, and *O*, and with the maximum controller capacity, described by the maximum rate a compensatory flux can deliver. Although our single loop controllers are a far cry from a physiologically regulated system, the analogy to Selye’s GAS is thought-provoking.

**Fig 28:**
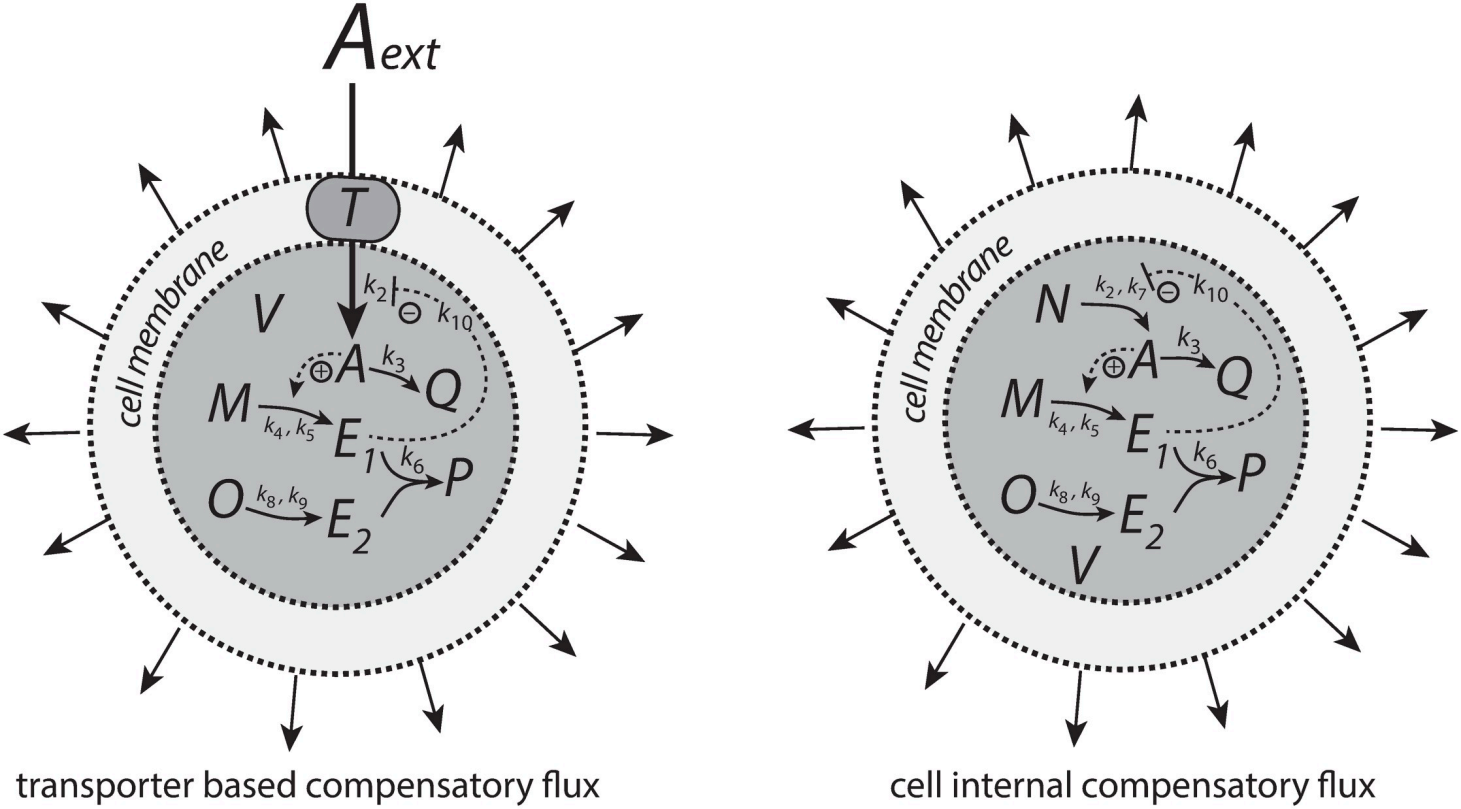
Antithetic integral controllers with motif 2 feedback structure and transporter based and cell internal compensatory fluxes.

Our results indicate that the type of kinetics realizing integral control and the structure of the negative feedback loop play essential roles in how a controller will perform. The antithetic integral controller has in the literature [22, 37, 38] so far only been considered in a motif 1 setting based on activation (Fig 1). However, its second-order integral controller part can be embedded into other feedback structures (S7 Text). Although the intension of this work was not to consider novel implementations of the antithetic integral controller, it is illustrative to see the controller’s improvement and limitations when considering the antithetic controller in a motif 2 background. Fig 28 shows two such implementations, one with a transporter based compensatory flux and the other with a cell internal one.

When merging the motif 2 structure with the antithetic integral controller, we keep the antithetic controller’s rate constant values, but change the *k*_2_ and the inhibition constant (*k*_10_) values to those used in the motif 2 controller calculations. For the transporter based motif 2 antithetic controller Eq 21 is now replaced by

$$\begin{array}{c} \dot{A}=\frac{k_{2}\cdot k_{10}}{k_{10}+E_{1}}\cdot \frac{1}{V}-k_{3}\cdot A-A(\frac{\dot{V}}{V}) \end{array}$$ (76)

while for the controller with a cell internal compensatory flux, Eq 56 is replaced by

$$\begin{array}{c} \dot{A}=(\frac{N}{k_{7}+N})\cdot (\frac{k_{2}k_{10}}{k_{10}+E_{1}})-k_{3}\cdot A-A(\frac{\dot{V}}{V}) \end{array}$$ (77)

**Fig 29:**
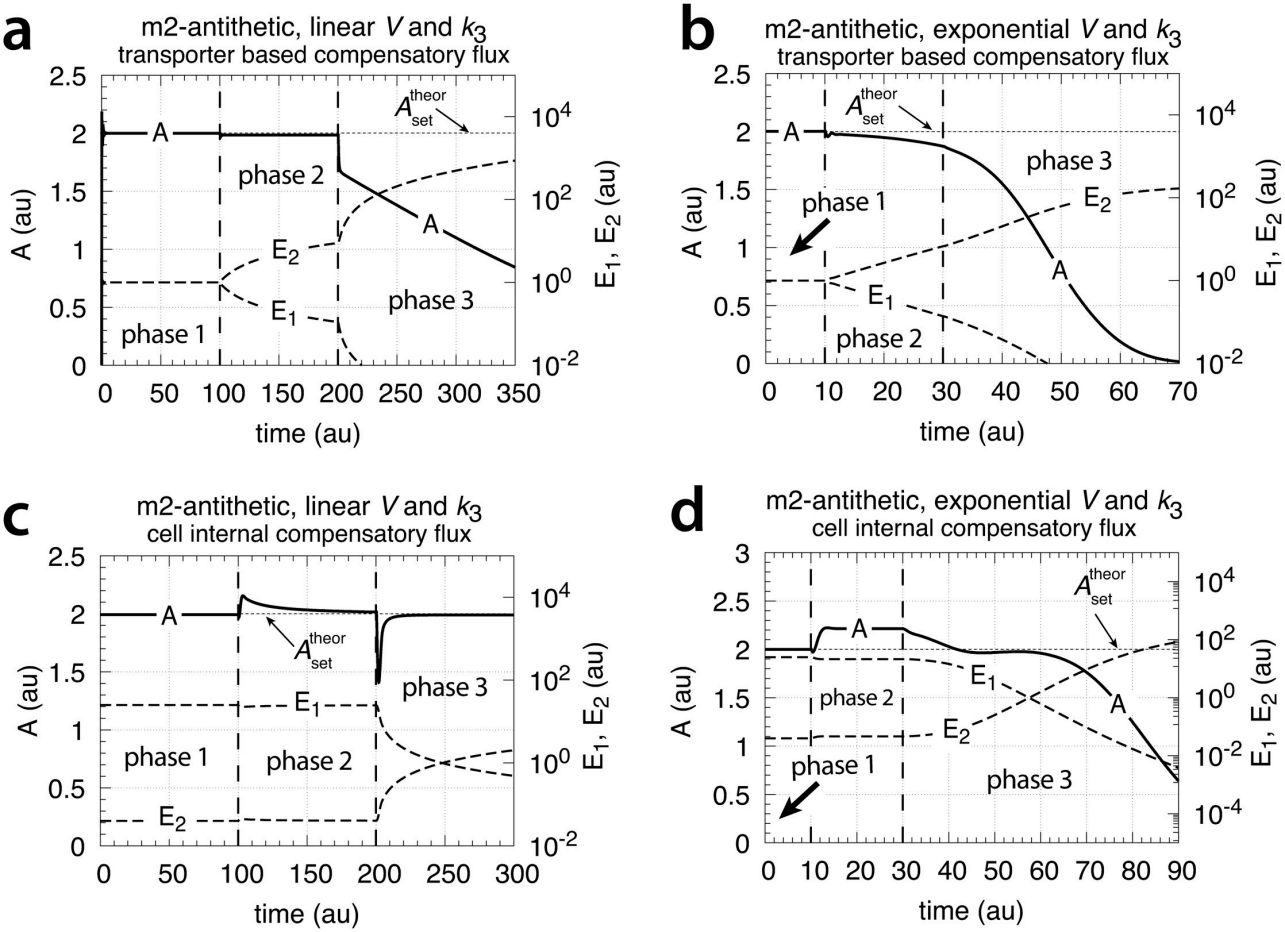
Performance of the antithetic controller in a motif 2 (m2) structural background (Fig 28). (a) The controller has a transporter mediated compensatory flux and is exposed to linear growth and increase in *k*_3_ as in Fig 9a. Same rate constants and initial concentrations as in Fig 9 with the exception that *k*_2_ = 1 × 10^5^, and *k*_10_ = 1 × 10^−3^. Note the minor offset in *A* during phase 2. (b) Same controller with rate constants as in (a), but exposed to exponential volume and *k*_3_ increases as in Fig 14. Initial concentrations: *A*_0_ = 2.0, *E*_1,0_ = *E*_2,0_ = 1.0, *M*_0_ = *O*_0_ = 1 × 10^5^. The controller is not able to oppose exponential growth. (c) Controller with a cell internal compensatory flux (Fig 28) and exposed to the conditions as in Fig 20. Same rate constants as in (a). Initial concentrations: *A*_0_ = 2.0, *E*_1,0_ = *E*_2,0_ = 25.0, *M*_0_ = *O*_0_ = 2 × 10^5^, *N*_0_ = *O*_0_ = 1 × 10^6^. The controller is fully capable to oppose linear growth together with a linear increase in *k*_3_. (d) Same controller with rate constants as in (c), but exposed to exponential volume and *k*_3_ increases as in Fig 14. Initial concentrations as in (c), but to avoid depletion of *M* and *O* initial concentrations of these compounds were raised to 1 × 10^6^. Note also here the overcompensation in the case growth occurs exponentially in phase 2. Despite the larger consumption rates of *M* and *O* in comparison with (c) the controller is not able to counteract both exponential increases in *V* and *k*_3_. See S7 Text for more details.

The other rate equations (Eqs 22–27) remain unchanged. Fig 29 shows the results when linear and exponential growth and *k*_3_ changes (see Figs 9a and 14) are applied to the controllers in Fig 28.

In comparison with the motif 1 antithetic structure, the usage of the motif 2 negative feedback shows a clear improvement in the antithetic controllers’ performance (compare Fig 29a, 29b, 29c and 29d with Figs 9, 15, 20 and 25, respectively). The feedback design based on a cell internal compensatory flux shows again a better performance in comparison when the compensatory flux is transporter based. On the other hand, when both *V* and *k*_3_ increase exponentially both motif 2 antithetic controllers are not able to keep *A* at a constant steady state. The reason for this is that the rate of derepression/removal in *E*_1_ is limited by its reaction with *E*_2_ and thereby is slower than the *E* removal in the motif 2 zero-order case.

From a biochemical perspective one may question how realistic a second-order removal of *E*_1_ and *E*_2_ is. Since practically all physiological reactions within a cell are catalyzed by enzymes, it appears to be an interesting alternative to study the performance of controllers when an enzyme uses *E*_1_ and *E*_2_ as substrates.

With respect to the autocatalytic implementation to achieve integral control [19–21], the occurrence of autocatalysis and positive feedback loops are becoming more and more recognized in signaling and homeostatic regulation [39–41]. As an illustration, in cortisol homeostasis ACTH signaling from the brain-pituitary system to the cortisol producing adrenals occurs by autocatalysis/positive feedback [42]. In blood sugar homeostasis insulin secretion is activated by several positive feedback/autocatalytic signaling pathways [43–45] to ensure an effective regulation in glucose concentration. These examples indicate the importance of additional “helper kinetics” (such as autocatalysis/positive feedback) to obtain a homeostatic regulation with optimum response and precision properties. For synthetic biology this means that knowledge about controller structure and their inherent kinetics are important aspects in the design and implementation of artificial regulatory units when trying to oppose the dilution effects of growth or other time-dependent perturbations.

### Outlook

The approach taken here is based on ordinary mass action kinetics and thereby is purely deterministic. In addition, we made the assumption that the cellular volume is well-mixed and homogenous. Both assumptions are subject to certain criticism, when applied to biochemical reactions within a cell. While in many cases a chemically reacting system can be treated as a continuous deterministic process, in other cases, in particular when the number of reacting molecules becomes low, reactions may better be described as discrete stochastic processes [46–48]. However, many, if not most of the stochastic approaches to describe cellular processes still treat (and require) that systems are treated as homogenous, thereby neglecting “recruiting” or “funneling” mechanisms which occur, for example, on the surface of cellular membranes, involve multiprotein complexes (“antenna”) in photon harvesting [49], or use substrate channeling (“tunnels”) in enzyme-catalyzed reactions [50].

Ignoring these spatial aspects, it may be mentioned, that the controller motifs 1 and 2 (Fig 1) based on zero-order and first-order autocatalysis are well-described by the Gillespie algorithm [46] and show an excellent correspondence between the stochastic and deterministic approach (P. Ruoff, unpublished results). The motif 1 antithetic controller has been shown to work well under stochastic conditions by exploiting noise, and achieves regulation where a similar deterministic approach apparently fails [21]. How these controllers behave under time-dependent perturbations and stochastic conditions is an interesting aspect which we would like to investigate in a later work.

Nevertheless, we feel that the deterministic calculations presented here give a first ranking between the various integral controllers when exposed to different growth laws and dilution kinetics.

## Supporting information

S1 Matlab A set of Matlab files showing the results of Figs 4, 7, 9, 11, 13, 15, 18, 20, 22, 24, 25, 26 and 27. (ZIP)

S1 Text Steady state of cell-internal-generated compound *A* without negative feedback. (PDF)

S2 Text Steady state of transporter-generated compound *A* without negative feedback. (PDF)

S3 Text Steady states and theoretical set-point for motif 1 zero-order controller. (PDF)

S4 Text Steady states and theoretical set-point for motif 1 second-order (antithetic) controller. (PDF)

S5 Text Steady states and theoretical set-point for motif 1 autocatalytic controller. (PDF)

S6 Text Steady states and theoretical set-point for motif 2 zero-order controller. (PDF)

S7 Text Novel antithetic integral controller arrangements and steady states in a motif 2 background. (PDF)
