# Supplementary figures and images for: Homeostatic controllers compensating for growth and perturbations
Source: PLoS One. 2019 Aug 12;14(8):e0207831. doi: 10.1371/journal.pone.0207831 (PMC6690524; doi:10.1371/journal.pone.0207831)

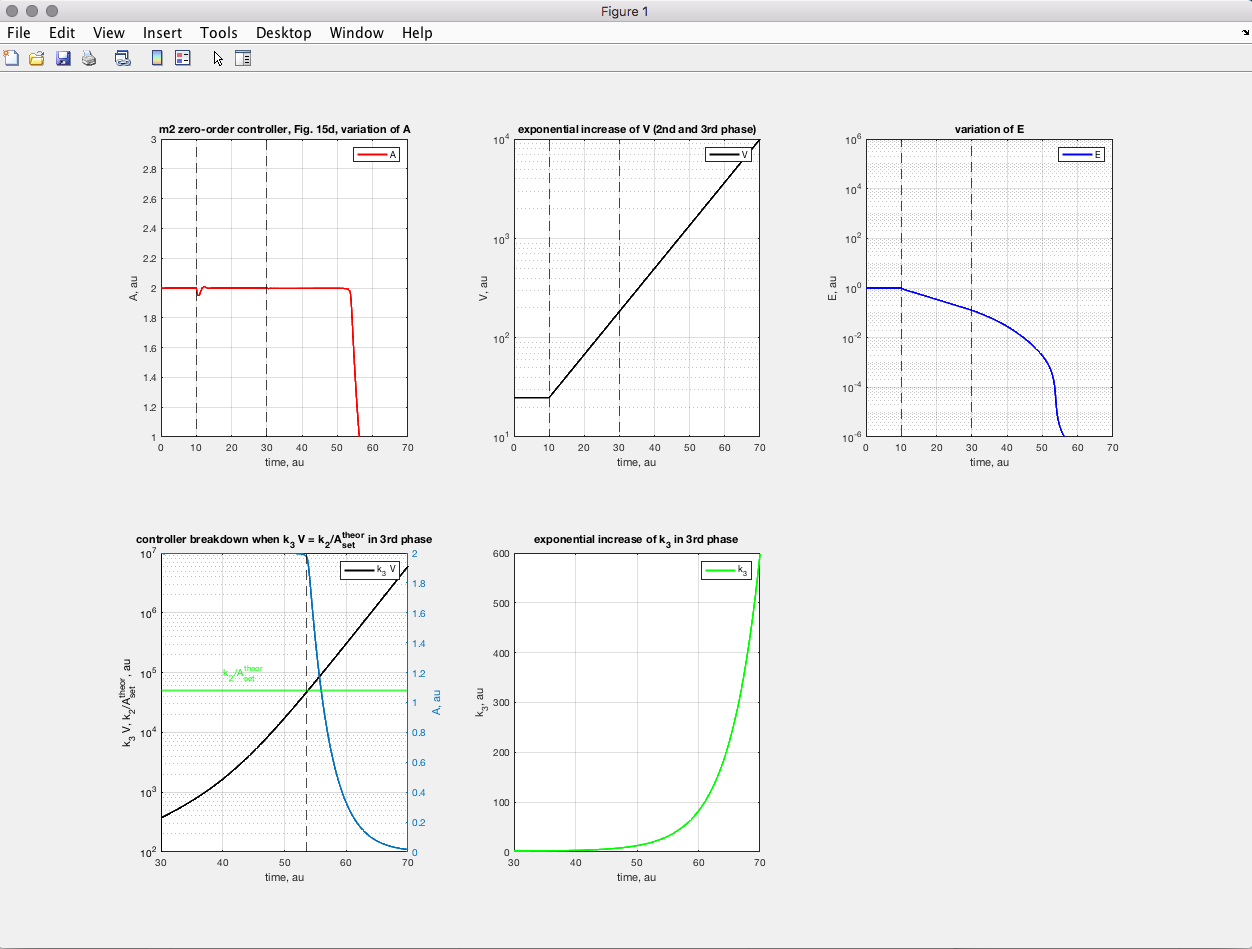

Supplement: S1 Matlab — (ZIP) [file pone.0207831.s001.zip › S1 Matlab/fig15d_matlab/Screen Shot.png]

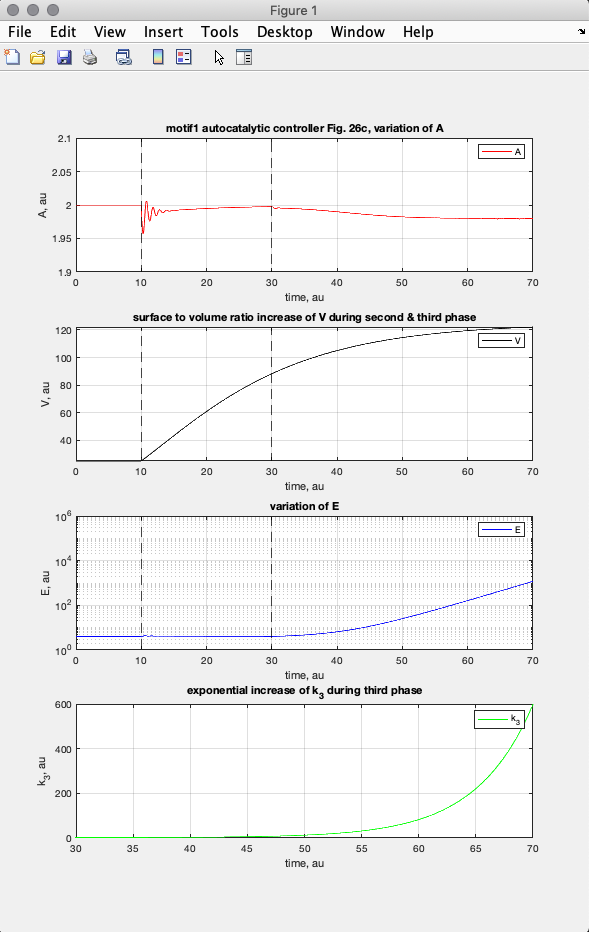

Supplement: S1 Matlab — (ZIP) [file pone.0207831.s001.zip › S1 Matlab/fig26c_matlab/Screenshot.png]

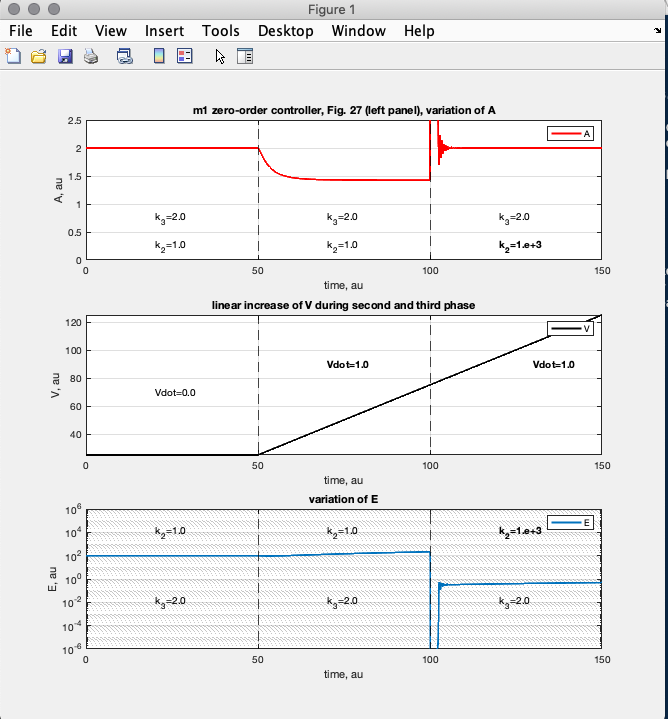

Supplement: S1 Matlab — (ZIP) [file pone.0207831.s001.zip › S1 Matlab/fig27_m1zo_matlab/Screenshot.png]

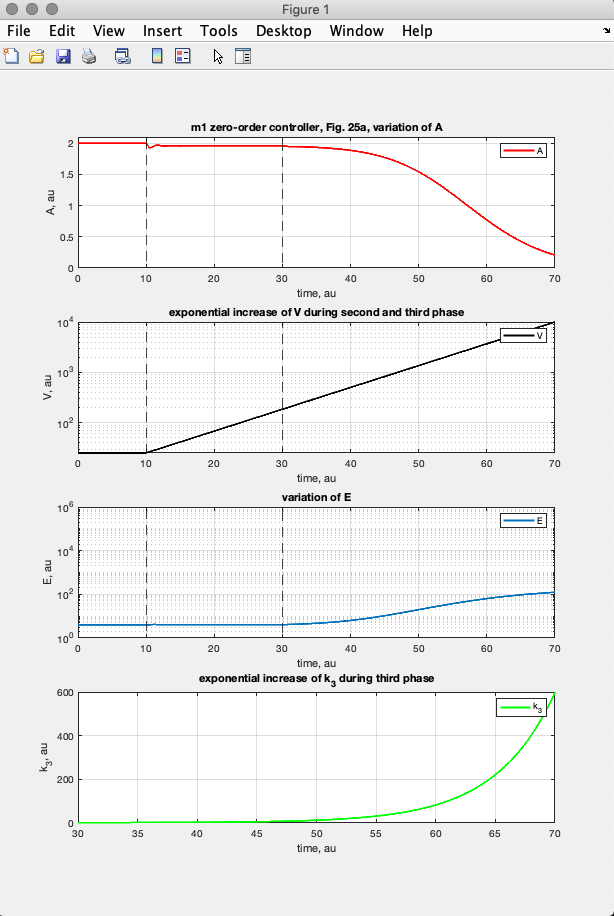

Supplement: S1 Matlab — (ZIP) [file pone.0207831.s001.zip › S1 Matlab/fig25a_matlab/Screenshot.png]

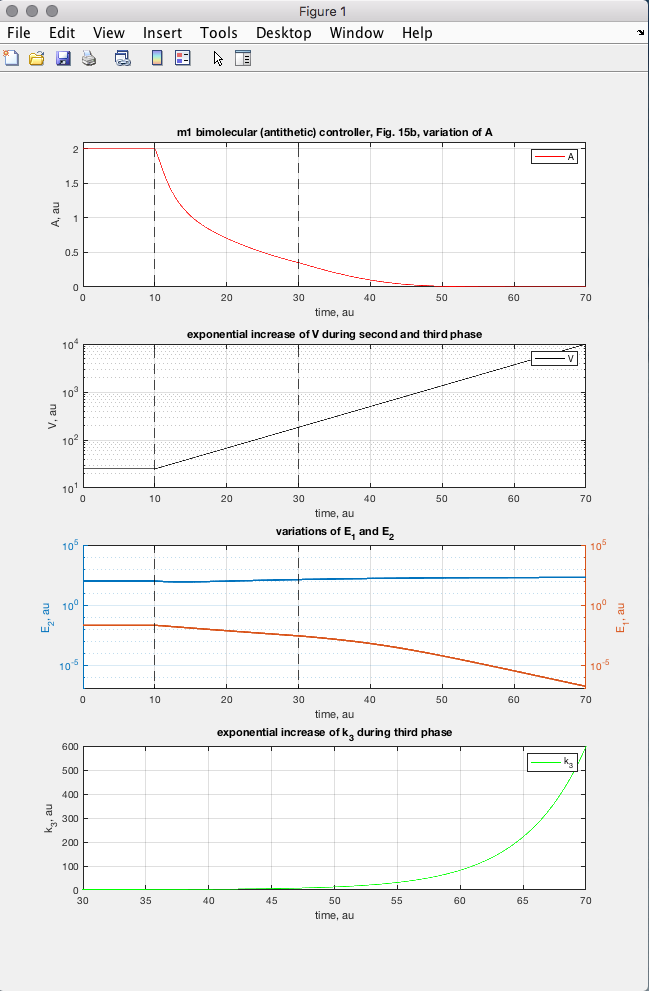

Supplement: S1 Matlab — (ZIP) [file pone.0207831.s001.zip › S1 Matlab/fig15b_matlab/Screen Shot.png]

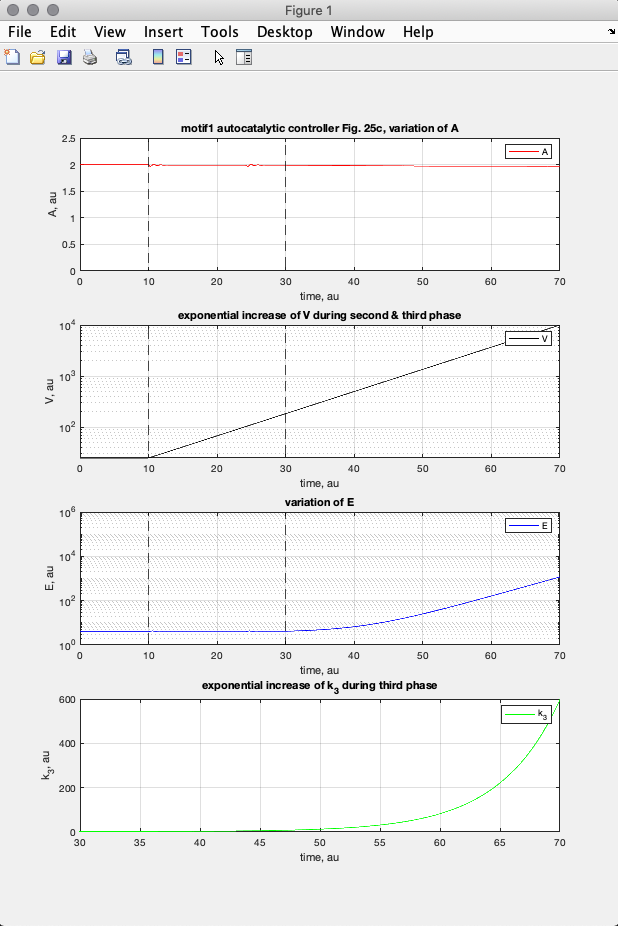

Supplement: S1 Matlab — (ZIP) [file pone.0207831.s001.zip › S1 Matlab/fig25c_matlab/Screenshot.png]

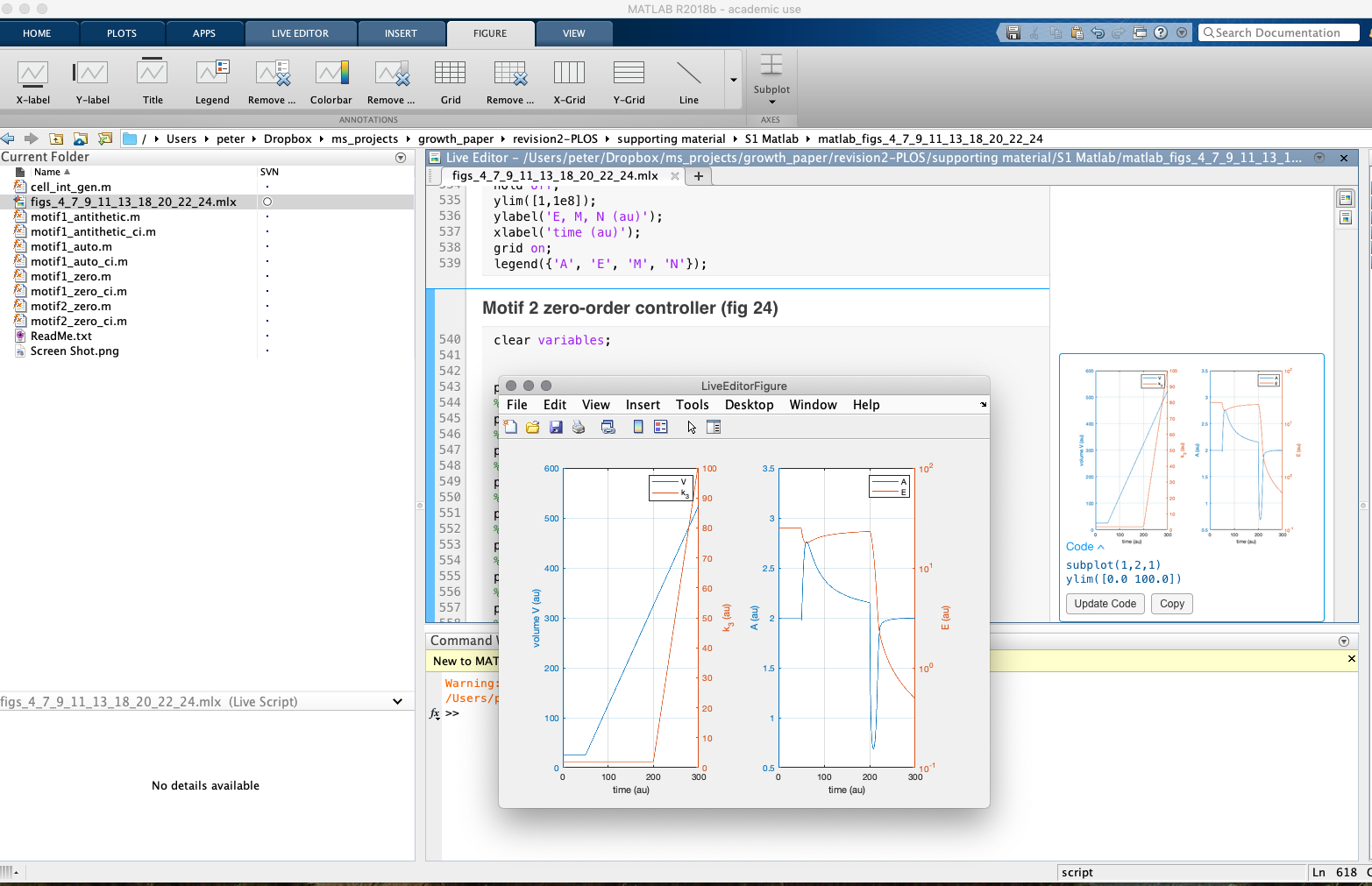

Supplement: S1 Matlab — (ZIP) [file pone.0207831.s001.zip › S1 Matlab/matlab_figs_4_7_9_11_13_18_20_22_24/Screenshot.png]

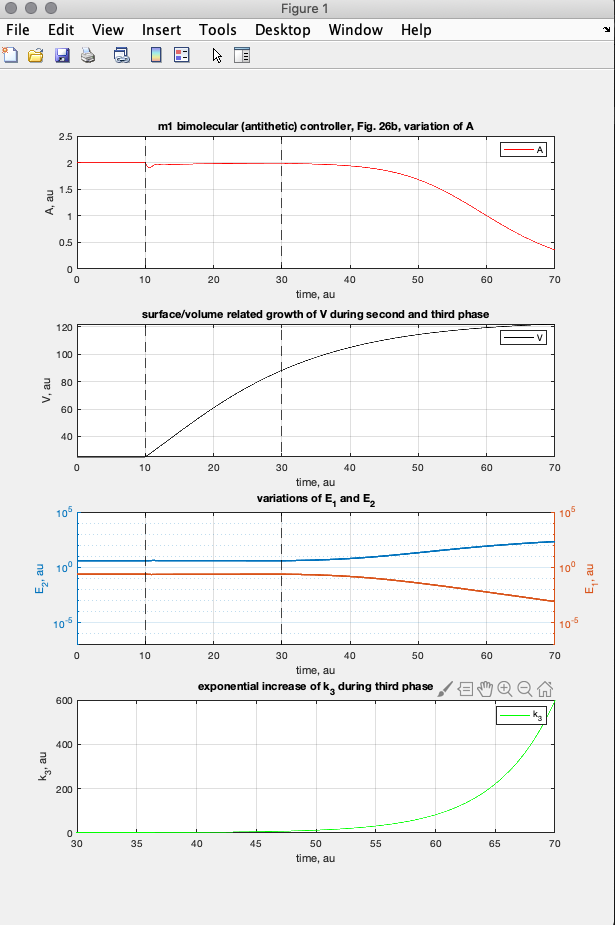

Supplement: S1 Matlab — (ZIP) [file pone.0207831.s001.zip › S1 Matlab/fig26b_matlab/Screenshot.png]

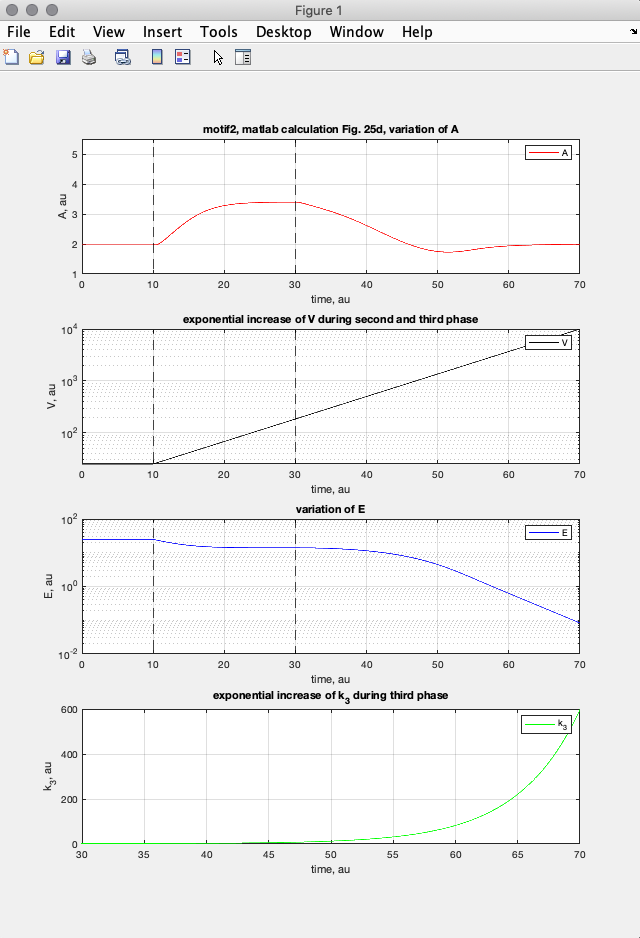

Supplement: S1 Matlab — (ZIP) [file pone.0207831.s001.zip › S1 Matlab/fig25d_matlab/Screenshot.png]

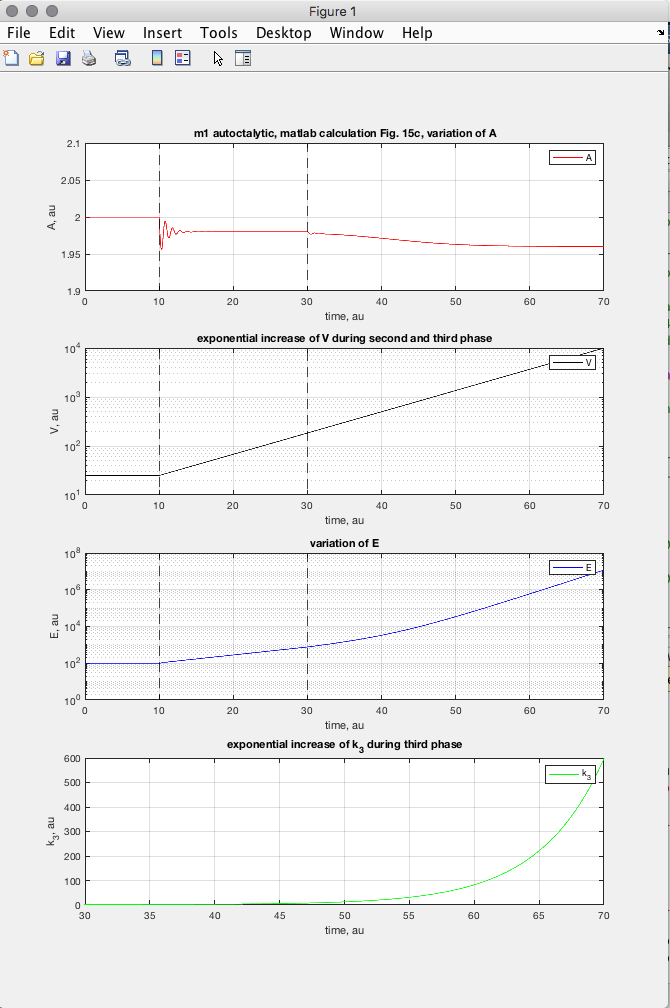

Supplement: S1 Matlab — (ZIP) [file pone.0207831.s001.zip › S1 Matlab/fig15c_matlab/Screen Shot.png]

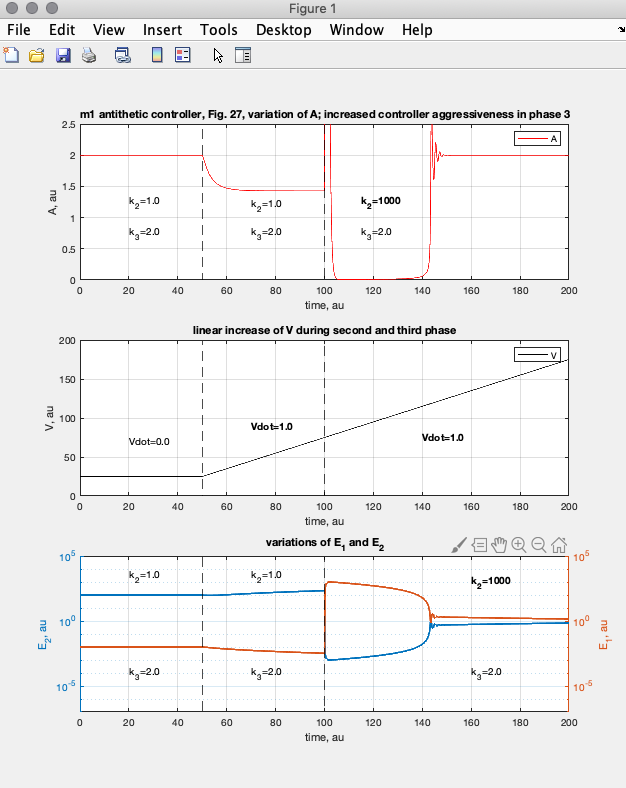

Supplement: S1 Matlab — (ZIP) [file pone.0207831.s001.zip › S1 Matlab/fig27_antithetic_matlab/Screenshot.png]

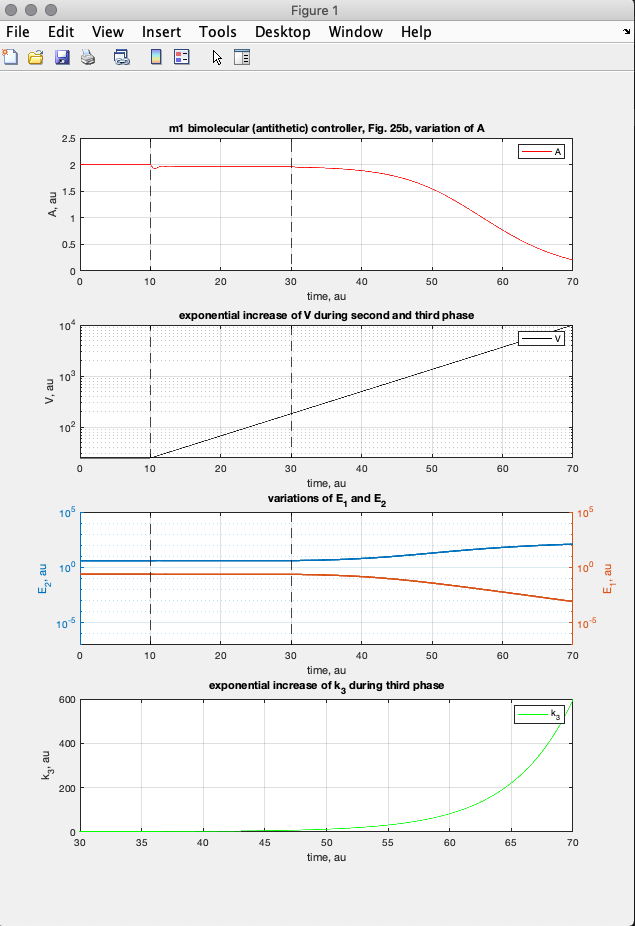

Supplement: S1 Matlab — (ZIP) [file pone.0207831.s001.zip › S1 Matlab/fig25b_matlab/Screenshot.png]

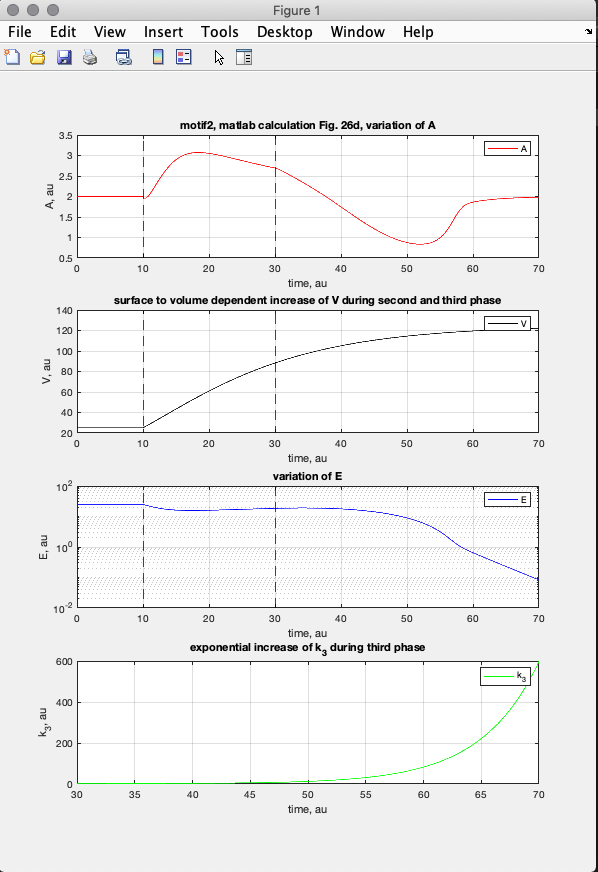

Supplement: S1 Matlab — (ZIP) [file pone.0207831.s001.zip › S1 Matlab/fig26d_matlab/Screenshot.png]

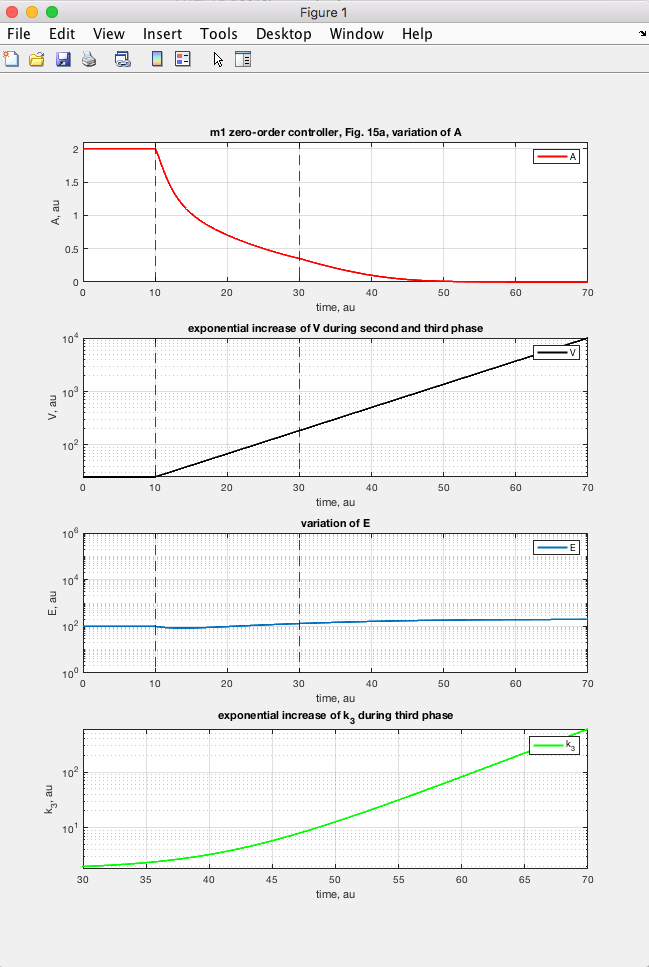

Supplement: S1 Matlab — (ZIP) [file pone.0207831.s001.zip › S1 Matlab/fig15a_matlab/Screen Shot.png]
