# Supplementary material for: Homeostatic controllers compensating for growth and perturbations
Source: PLoS One. 2019 Aug 12;14(8):e0207831. doi: 10.1371/journal.pone.0207831 (PMC6690524; doi:10.1371/journal.pone.0207831)
Supplement: S1 Text — (PDF) [file pone.0207831.s002.pdf]

Supporting Material, File S1 Text

Homeostatic Controllers Compensating for Growth and Perturbations

P. Ruoff[1*], O. Agafonov[1], D. M. Tveit[2], K. Thorsen[2], T. Drengstig[2]
[1]Centre for Organelle Research
[2]Department of Electrical Engineering and Computer Science,
University of Stavanger, Stavanger, Norway

*Corresponding author. Address: Centre for Organelle Research, University of Stavanger, N-4036 Stavanger, Norway, Tel.: (47) 5183-1887, Fax: (47) 5183-1750, E-mail: peter.ruoff@uis.no

Steady state of cell-internal-generated compound A without negative feedback

Integrating $\dot{V}{=}k_1$ from V_0 to $V(t)$ and from time zero to time t gives

$$V(t) = k_1 t + V_0 \tag{S1}$$

Inserting Eq. S1 into the equation $\dot{E} = -E\dot{V}/V$ (Eq. 10), we get

$$\dot{E} = -E\left(\frac{k_1}{k_1 t{+}V_0}\right) = -E\left(\frac{1}{t{+}\alpha}\right) \tag{S2}$$

where $\alpha = V_0/k_1$. Separation of variables gives

$$\frac{\mathrm{d}E}{E} = \frac{\mathrm{d}t}{t{+}\alpha} \tag{S3}$$

Integrating from time zero to t and from E_0 to $E(t)$ we get

$$\ln\left(\frac{E}{E_0}\right) = \ln\left(\frac{\alpha}{\alpha{+}t}\right) \quad \Rightarrow \quad E = E_0\left(\frac{\alpha}{\alpha{+}t}\right) \tag{S4}$$

Inserting E from Eq. S4 into the rate equation for A (Eq. 11)

$$\dot{A} = k_2{\cdot}E - A{\cdot}\frac{\dot{V}}{V} = k_2{\cdot}E - A{\cdot}\frac{k_1}{V_0{+}k_1{\cdot}t} = k_2{\cdot}E - A{\cdot}\frac{1}{\alpha{+}t} \tag{S5}$$

we get the following rate equation for A

$$\dot{A} + \frac{A}{\alpha{+}t} = k_2 E_0\left(\frac{\alpha}{\alpha{+}t}\right) \tag{S6}$$

The solution of Eq. S6 is given by Eq. 11, i.e.,

$$A(t) = k_2 E_0 \alpha + (A_0 - k_2 E_0 \alpha)\left(\frac{\alpha}{\alpha{+}t}\right) \tag{S7}$$
