# Supplementary material for: Homeostatic controllers compensating for growth and perturbations
Source: PLoS One. 2019 Aug 12;14(8):e0207831. doi: 10.1371/journal.pone.0207831 (PMC6690524; doi:10.1371/journal.pone.0207831)
Supplement: S2 Text — (PDF) [file pone.0207831.s003.pdf]

# Supporting Material, File S2 Text

# Homeostatic Controllers Compensating for Growth and Perturbations

P. Ruoff[1*], O. Agafonov[1], D. M. Tveit[2], K. Thorsen[2], T. Drengstig[2]
[1]Centre for Organelle Research
[2]Department of Electrical Engineering and Computer Science,
University of Stavanger, Stavanger, Norway

*Corresponding author. Address: Centre for Organelle Research, University of Stavanger, N-4036 Stavanger, Norway, Tel.: (47) 5183-1887, Fax: (47) 5183-1750, E-mail: peter.ruoff@uis.no

**Steady state of transporter-generated compound $A$ without negative feedback**

We start with Eq. 15 where transporter $T$ pumps external $A$ ($A_{ext}$) into a constantly growing cell ($\dot{V}$=constant)

$$\dot{A} = \frac{k_2{\cdot}T}{V} - A\left(\frac{\dot{V}}{V}\right) \tag{S1}$$

We assume that the surface concentration of $T$ is constant and that the pump rate is zero-order with respect to the external $A$ concentration.

The steady state of $A$ is given by setting Eq. S1 to zero, which gives

$$\dot{A} = \frac{k_2{\cdot}T}{V} - A\left(\frac{\dot{V}}{V}\right) = 0 \quad \Rightarrow \quad A_{ss} = \frac{k_2{\cdot}T}{\dot{V}} \tag{S2}$$

independent of the initial concentration of $A$.

In case there is a first-order removal of cellular $A$ with respect to $A$ the rate equation becomes

$$\dot{A} = \frac{k_2{\cdot}T}{V} - k_3{\cdot}A - A\left(\frac{\dot{V}}{V}\right) = 0 \tag{S3}$$

Setting Eq. S3 to zero leads to

$$A_{ss} = \frac{k_2{\cdot}T}{k_3V + \dot{V}} \;\rightarrow\; 0 \quad \text{as} \quad \text{V} \rightarrow \infty \tag{S4}$$

In case the removal of cellular $A$ is zero-order with respect to $A$ (for example by an enzyme removing A at maximum velocity $V_{max}$), then in this case the steady state condition

$$\dot{A} = \frac{k_2{\cdot}T}{V} - V_{max} - A\left(\frac{\dot{V}}{V}\right) = 0 \tag{S5}$$

gives

$$A_{ss} = \frac{1}{\dot{V}} \left( k_2 \cdot T - V_{max} \cdot V \right) \tag{S6}$$

As the volume $V$ grows there will be a critical volume $V_{crit}$=$k_2T/V_{max}$ at which $A_{ss}$ becomes zero.
