# Supplementary material for: Homeostatic controllers compensating for growth and perturbations
Source: PLoS One. 2019 Aug 12;14(8):e0207831. doi: 10.1371/journal.pone.0207831 (PMC6690524; doi:10.1371/journal.pone.0207831)
Supplement: S3 Text — (PDF) [file pone.0207831.s004.pdf]

Supporting Material, File S3 Text

Homeostatic Controllers Compensating for Growth and Perturbations

P. Ruoff[1*], O. Agafonov[1], D. M. Tveit[2], K. Thorsen[2], T. Drengstig[2]
[1]Centre for Organelle Research
[2]Department of Electrical Engineering and Computer Science,
University of Stavanger, Stavanger, Norway

*Corresponding author. Address: Centre for Organelle Research, University of Stavanger, N-4036 Stavanger, Norway, Tel.: (47) 5183-1887, Fax: (47) 5183-1750, E-mail: peter.ruoff@uis.no

Steady states and theoretical set-point for motif 1 zero-order controller

Transporter-based compensatory flux with constant values of $\dot{V}$ and $\dot{k}_3$

The rate equations for A and E when the compensatory flux is transporter based, are

$$\dot{A} = \frac{k_2 E}{V} - k_3 \cdot A - A\left(\frac{\dot{V}}{V}\right) \tag{S1}$$

$$\dot{E} = k_4 - k_6 \cdot A - E\left(\frac{\dot{V}}{V}\right) \tag{S2}$$

with $\frac{M}{k_5+M}$=$\frac{E}{k_7+E}$=1 (see Eq. 16). For constant V ($\dot{V}$=0) and k_3 ($\dot{k}_3$=0), Eq. S2 defines the theoretical set-point, i.e.

$$\dot{E} = k_4 - k_6 \cdot A - A\left(\frac{\dot{V}}{V}\right) = 0 \quad \Rightarrow \quad A_{ss} = A_{set}^{theor} = \frac{k_4}{k_6} \tag{S3}$$

As long as $\frac{E}{k_7+E}$=1 the controller will for any step-wise perturbation in k_3 or V move A_{ss} to A_{set}^{theor}.
However, for constant $\dot{V}$ and $\dot{k}_3$, Eq. S3 is no longer valid. In case the volume V increases linearly, E needs to increase in order to oppose the dilution of A. To get an estimate of A_{ss} for constant $\dot{V}$ and $\dot{k}_3$, we take the double time derivative of A, and set $\ddot{A}$ and $\dot{A}$ to zero

$$\ddot{A} = \frac{k_2 \dot{E}}{V} - \frac{k_2 E \dot{V}}{V^2} - \dot{k}_3 A + A\left(\frac{\dot{V}}{V}\right)^2 = 0 \tag{S4}$$

Inserting Eq. S2 into Eq. S4 gives

$$\frac{k_2}{V}\left[k_4 - k_6 \cdot A - E\left(\frac{\dot{V}}{V}\right)\right] - k_2 \cdot E\left(\frac{\dot{V}}{V^2}\right) - \dot{k}_3 A + A\left(\frac{\dot{V}}{V}\right)^2 = 0 \tag{S5}$$

Multiplying Eq. S5 with V leads to

$$k_2k_4 - k_2k_6{\cdot}A - 2k_2E\left(\frac{\dot{V}}{V}\right) - \dot{k}_3A{\cdot}V + \underbrace{A\left(\frac{\dot{V}^2}{V}\right)}_{\to 0} = 0 \tag{S6}$$

Assuming steady state conditions in Eq. S1 and neglecting there the $A\dot{V}/V$ term we can approximately write

$$\dot{A} = \frac{k_2E}{V} - k_3{\cdot}A - A\left(\frac{\dot{V}}{V}\right) = 0 \quad \Rightarrow \quad \frac{k_2E}{V} = k_3{\cdot}A_{ss} \tag{S7}$$

with E and V increasing. Inserting the right-hand side of Eq. S7 into Eq. S6 gives

$$k_2k_4 - k_2k_6{\cdot}A_{ss} - 2k_3A_{ss}\dot{V} - \dot{k}_3A_{ss}{\cdot}V = 0 \tag{S8}$$

Solving for A_{ss}, we get

$$A_{ss} = \frac{k_2k_4}{k_2k_6{+}2k_3\dot{V}{+}\dot{k}_3V} \tag{S9}$$

In phase 2 of Fig. 7 we have $\dot{k}_3$=0. For k_2=2.0, k_3=2.0, k_4=20.0, k_4=10.0, and $\dot{V}$=2.0, A_{ss} is calculated after Eq. S9 to be 1.25, while the numerical value of A_{ss} is 1.11. When in phase 3 (Fig. 7) $\dot{k}_3$=1.0, Eq. S9 indicates, as observed, that A_{ss} will go to zero as V increases.

Cell-internal compensatory flux with constant values of $\dot{V}$ and $\dot{k}_3$

Rate equations for A and E (Eqs. 50 and 51) are written as

$$\dot{A} = k_3{\cdot}E - k_3{\cdot}A - A\left(\frac{\dot{V}}{V}\right) \tag{S10}$$

$$\dot{E} = k_4 - k_6{\cdot}A - E\left(\frac{\dot{V}}{V}\right) \tag{S11}$$

by using $N/(k_7{+}N){=}M/(k_5{+}M){=}1$. In addition, $E/(k_8{+}E){=}1$ giving the controller ideal behavior/precision for step-wise perturbations in k_3 and V. Calculating $\ddot{A}$ and setting $\ddot{A}$ and $\dot{A}$ to zero, gives

$$\ddot{A}{=}k_2{\cdot}\dot{E}{-}\dot{k_3}A{+}A\left(\frac{\dot{V}}{V}\right)^2{=}k_2\left[k_4{-}k_6{\cdot}A{-}E\left(\frac{\dot{V}}{V}\right)\right]{-}\dot{k_3}A{+}A\left(\frac{\dot{V}}{V}\right)^2=0 \tag{S12}$$

Neglecting the $\dot{V}/V$ terms (steady state when time t and volume V become large) leads to Eq. 53

$$k_2k_4 = k_2k_6A_{ss} + \dot{k_3}A_{ss} \quad \Rightarrow \quad A_{ss} = \frac{k_2k_4}{k_2k_6{+}\dot{k_3}} \tag{S13}$$

Model calculations at the end of phase 3 in Fig. 18 (k_2=1.0, k_4=20.0, k_6=10.0, and $\dot{k_3}$=1.0) show an A_{ss}=1.75, while the estimated value from Eq. S13 gives a value of 1.82.
