# Supplementary material for: Homeostatic controllers compensating for growth and perturbations
Source: PLoS One. 2019 Aug 12;14(8):e0207831. doi: 10.1371/journal.pone.0207831 (PMC6690524; doi:10.1371/journal.pone.0207831)
Supplement: S4 Text — (PDF) [file pone.0207831.s005.pdf]

Supporting Material, File S4 Text

Homeostatic Controllers Compensating for Growth and Perturbations

P. Ruoff[1*], O. Agafonov[1], D. M. Tveit[2], K. Thorsen[2], T. Drengstig[2]
[1]Centre for Organelle Research
[2]Department of Electrical Engineering and Computer Science,
University of Stavanger, Stavanger, Norway

*Corresponding author. Address: Centre for Organelle Research, University of Stavanger, N-4036 Stavanger, Norway, Tel.: (47) 5183-1887, Fax: (47) 5183-1750, E-mail: peter.ruoff@uis.no

Steady states and theoretical set-point for motif 1 second-order (antithetic) controller

Transporter-based compensatory flux with constant values of $\dot{V}$ and $\dot{k}_3$

The rate equations (Eqs. 21-23) are written as

$$\dot{A} = \frac{k_2 E_2}{V} - k_3 \cdot A - A\left(\frac{\dot{V}}{V}\right) \tag{S1}$$

$$\dot{E}_1 = k_4 \cdot A - k_6 \cdot E_1 \cdot E_2 - E_1\left(\frac{\dot{V}}{V}\right) \quad \text{with} \quad \frac{M}{k_5 + M} = 1 \tag{S2}$$

$$\dot{E}_2 = k_8 - k_6 \cdot E_1 \cdot E_2 - E_2\left(\frac{\dot{V}}{V}\right) \quad \text{with} \quad \frac{O}{k_9 + O} = 1 \tag{S3}$$

By setting $\dot{E}_1$=0 and neglecting the dilution term (considering $V \gg \dot{V}$) steady state conditions require

$$k_4 \cdot A = k_6 \cdot E_1 \cdot E_2 \tag{S4}$$

Calculating the double time derivative of A and setting it and $\dot{A}$ to zero gives

$$\ddot{A} = \frac{k_2 \dot{E}_2}{V} - \frac{k_2 E_2 \dot{V}}{V^2} - \dot{k}_3 \cdot A + A\left(\frac{\dot{V}}{V}\right)^2 = 0 \tag{S5}$$

Inserting $\dot{E}_2$ from Eq. S3 into Eq. S5

$$\ddot{A} = \frac{k_2}{V}\left[k_8 - \underbrace{k_6 \cdot E_1 \cdot E_2}_{k_4 \cdot A} - E_2\left(\frac{\dot{V}}{V}\right)\right] - \frac{k_2 E_2 \dot{V}}{V^2} - \dot{k}_3 \cdot A + A\left(\frac{\dot{V}}{V}\right)^2 = 0 \tag{S6}$$

Multiplying Eq. S6 by V yields

$$\ddot{A} = k_2 \left[k_8 - k_4{\cdot}A - E_2 \left(\frac{\dot{V}}{V} \right) \right] - \frac{k_2 E_2}{V}{\cdot}\dot{V} - \dot{k_3}{\cdot}A{\cdot}V + \underbrace{A \left(\frac{\dot{V}^2}{V} \right)}_{\approx 0} = 0 \quad \text{(S7)}$$

giving

$$k_2 k_8 - k_2 k_4{\cdot}A_{ss} - 2{\cdot}\underbrace{\frac{k_2 E_2}{V}}_{k_3 \cdot A_{ss}}{\cdot}\dot{V} - \dot{k_3}{\cdot}A_{ss}{\cdot}V = 0 \quad \text{(S8)}$$

using the steady state condition from Eq. S1 that $k_2 E_2/V{=}k_3 A_{ss}$. Solving for A_{ss} gives

$$A_{ss} = \frac{k_2 k_8}{k_2 k_4 {+} 2 k_3 \dot{V} {+} \dot{k_3} V} \quad \text{(S9)}$$

which is analogous to the A_{ss} expression for the motif 1 zero-order controller (see S3 Text, Eq. S9). As for the motif 1 zero-order controller A_{ss} will go to zero for the antithetic controller when $\dot{k_3} \neq 0$ (Fig. 9, phase 3).

Cell-internal-based compensatory flux with constant values of $\dot{V}$ and $\dot{k_3}$

The rate equation for A with an cell internal compensatory flux is given by

$$\dot{A} = k_2 E_2 - k_3{\cdot}A - A \left(\frac{\dot{V}}{V} \right) \quad \text{(S10)}$$

where in Eq. 56 $N/(k_7{+}N)$ is set to 1.0. The rate equations for E_1 and E_2 are as given by Eqs. S2 and S3, respectively.

Calculating $\ddot{A}$ from Eq. S10 gives

$$\ddot{A} = k_2 \dot{E}_2 - \dot{k_3}{\cdot}A + A \left(\frac{\dot{V}}{V} \right)^2 = 0 \quad \text{(S11)}$$

Inserting $\dot{E}_2$ from Eq. S3 into Eq. S11 gives

$$k_2\left[k_8 - \underbrace{k_6{\cdot}E_1{\cdot}E_2}_{k_4\cdot A_{ss}} - E_2\left(\underbrace{\frac{\dot{V}}{V}}_{\to 0}\right)\right] - \dot{k_3}{\cdot}A_{ss} + A_{ss}\left(\underbrace{\frac{\dot{V}}{V}}_{\to 0}\right)^2 = 0 \tag{S12}$$

Collecting the A_{ss} terms and observing that $\dot{V}/V \to 0$, we get

$$k_2k_8 = k_2k_4{\cdot}A_{ss} + \dot{k_3}A_{ss} \quad \Rightarrow \quad A_{ss} = \frac{k_2k_8}{k_2k_4 + \dot{k_3}} \tag{S13}$$

Note, that also in case of a cell-internal generated compensatory flux the steady state expressions for A for the motif 1 zero-order and the antithetic controllers show the same behaviors (compare S3 Text Eq. S13) with Eq. S13 above.
