# Supplementary material for: Homeostatic controllers compensating for growth and perturbations
Source: PLoS One. 2019 Aug 12;14(8):e0207831. doi: 10.1371/journal.pone.0207831 (PMC6690524; doi:10.1371/journal.pone.0207831)
Supplement: S5 Text — (PDF) [file pone.0207831.s006.pdf]

Supporting Material, File S5 Text

Homeostatic Controllers Compensating for Growth and Perturbations

P. Ruoff[1*], O. Agafonov[1], D. M. Tveit[2], K. Thorsen[2], T. Drengstig[2]
[1]Centre for Organelle Research
[2]Department of Electrical Engineering and Computer Science,
University of Stavanger, Stavanger, Norway

*Corresponding author. Address: Centre for Organelle Research, University of Stavanger, N-4036 Stavanger, Norway, Tel.: (47) 5183-1887, Fax: (47) 5183-1750, E-mail: peter.ruoff@uis.no

Steady states and theoretical set-point for motif 1 autocatalytic controller

Transporter-based compensatory flux with constant values of $\dot{V}$ and $\dot{k}_3$ (k_E^{in}=k_E^{out}=1×10^{-5})

The rate equations Eqs. 33-34 are rewritten in the following form

$$\dot{A} = \frac{k_2 E}{V} - k_3 \cdot A - A\left(\frac{\dot{V}}{V}\right) \tag{S1}$$

$$\dot{E} = k_4 \cdot E - k_6 \cdot A \cdot E - E\left(\frac{\dot{V}}{V}\right) \tag{S2}$$

where in Eq. 34 $M/(k_5{+}M)$=1.0 and the term $k_E^{in}{-}k_E^{out}{\cdot}E$ is neglected. In addition, sufficent M is present to avoid a controller breakdown as shown in phase 3 of Fig. 11.

Calculating $\ddot{A}$ from Eq. S1 gives

$$\ddot{A} = \frac{k_2 \dot{E}}{V} - \frac{k_2 E \dot{V}}{V^2} - \dot{k}_3 \cdot A + A\left(\frac{\dot{V}}{V}\right)^2 = 0 \tag{S3}$$

Note that $\ddot{V}$=0 since $\dot{V}$=constant. In addition we assume steady state in A such that $\dot{A}$=0.

Inserting Eq. S2 into Eq. S3 gives

$$\frac{k_2}{V}\left[k_4 E - k_6 \cdot E \cdot A - E\left(\frac{\dot{V}}{V}\right)\right] - \frac{k_2 E \dot{V}}{V^2} - \dot{k}_3 A + A\left(\frac{\dot{V}}{V}\right)^2 = 0 \tag{S4}$$

Collecting terms gives

$$\underbrace{\frac{k_2E}{V}}_{k_3A_{ss}}\cdot k_4 - \underbrace{\frac{k_2E}{V}}_{k_3A_{ss}}\cdot k_6\cdot A_{ss} - \frac{2E}{V}\left(\underbrace{\frac{\dot{V}}{V}}_{\to 0}\right) - \dot{k}_3A_{ss} + A_{ss}\left(\underbrace{\frac{\dot{V}}{V}}_{\to 0}\right)^2 = 0 \tag{S5}$$

Rearranged we get

$$k_3k_4A_{ss} - k_3k_6A_{ss}^2 - \dot{k}_3A_{ss} = 0 \quad \Rightarrow \quad A_{ss} = \frac{k_3k_4 - \dot{k}_3}{k_3k_6} = A_{set}^{theor} - \frac{\dot{k}_3}{k_3k_6} \tag{S6}$$

where A_{set}^{theor}=k_4/k_6 for step-wise perturbations.
To show that the autocatalytic controller can manage a k_3 perturbation of the form

$$k_3(t) = k_{3,0} + a\cdot t^n \tag{S7}$$

we note that the ratio $\dot{k}_3/k_3 \to 0$ as $t \to \infty$, i.e.

$$\lim_{t\to\infty}\left\{\frac{\dot{k}_3}{k_3}\right\} = \lim_{t\to\infty}\left\{\frac{n\cdot at^{n-1}}{k_{3,0}+at^n}\right\} \overset{\text{L'Hôpital}}{=} n(n-1)\lim_{t\to\infty}\left\{\frac{1}{t}\right\} = 0 \tag{S8}$$

such that the term $\dot{k}_3/(k_3k_6)$ in Eq. S6 goes to zero.

Transporter-based compensatory flux with constant values of $\dot{V}$ and $\dot{k}_3$ and non-negligible k_E^{in} and k_E^{out} terms (k_E^{in}=k_E^{out}=10.0)

Starting with rate equation 34 (now S9):

$$\dot{E} = k_4\cdot E - k_6\cdot A\cdot E + k_E^{in} - k_E^{out}\cdot E - E\left(\frac{\dot{V}}{V}\right) \tag{S9}$$

Inserting Eq. S9 into Eq. S3 gives:

$$\ddot{A}=\frac{k_2}{V}\left[k_4E-k_6\cdot E\cdot A + k_E^{in}-k_E^{out}\cdot E-E\left(\frac{\dot{V}}{V}\right)\right]-\frac{k_2E\dot{V}}{V^2}-\dot{k}_3A+A\left(\frac{\dot{V}}{V}\right)^2 \tag{S10}$$

Looking for the steady state when V and k_3 increase linearly ($\dot{V}$ and $\dot{k}_3$ are constant), the $\dot{V}/V$ and $\dot{V}/V^2$ terms are neglected by assuming that $\dot{V} \ll V$. For $\dot{A}$ this leads to

$$\dot{A} = \frac{k_2 E}{V} - k_3 \cdot A - A\left(\frac{\dot{V}}{V}\right) \approx \frac{k_2 E}{V} - k_3 \cdot A \tag{S11}$$

which, when setting Eq. S11 to zero, gives the relationship between the steady state in A, A_{ss}, and the changing E, V, and k_3 values, i.e.,

$$E = \left(\frac{k_3}{k_2}\right) \cdot V \cdot A_{ss} \tag{S12}$$

Setting Eq. S10 to zero, neglecting the $\dot{V}/V$ and $\dot{V}/V^2$ terms and inserting E from Eq. S12 into Eq. S10, results in the expression for A_{ss}

$$A_{ss}^2 - A_{ss}\left(\frac{k_4}{k_6} - \frac{k_E^{out}}{k_6} - \frac{\dot{k}_3}{k_3 k_6} + \frac{k_2 k_E^{in}}{V}\right) = 0 \tag{S13}$$

For increasing k_3 and V the terms $\dot{k}_3/k_3 k_6$ and $k_2 k_E^{in}/V$ become small in comparison with $(k_4 - k_E^{out})/k_6$ such that the new set-point is approximately

$$A_{ss} \approx \frac{k_4 - k_E^{out}}{k_6} \tag{S14}$$

Fig. S1 illustrates the change in set-point for $k_E^{in} = k_E^{out} = 10.0$, using the rate constant values from Fig. 11. During the first phase the system is as in Fig. 11, with $k_E^{in} = k_E^{out} = 1 \times 10^{-5}$. In phases 2 and 3 the values of k_E^{in} and k_E^{out} are changed 10.0. Eq. S14 predicts a set-point of 1.0. In phase 2 of Fig. S1 the numerically calculated A_{ss} is 1.001, while in phase 3 this value is 0.9996, indicating that Eq. S14 describes the new set-point quite well.

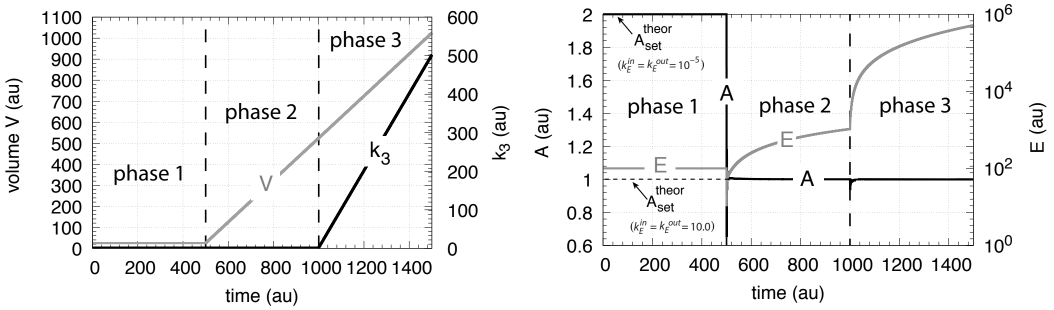

Figure S1. Performance of the motif 1 autocatalytic controller (Eqs. 33-35). Phase 1: constant volume V and constant k_3. Initial concentrations and rate constant values (at steady state) as in Fig. 11: V_0=25.0, $\dot{V}$=0.0, A_0=2.0, E_0=100.0, M_0=1 × 10^{12}, k_2=1.0, k_3=2.0, $\dot{k}_3$=0.0, k_4=20.0, k_5=1 × 10^{-6}, k_6=10.0, and k_E^{in}=k_E^{out}=1×10^{-5}. The controller keeps A at its set-point at A_{set}^{theor}=k_4/k_6=2.0. Phase 2: rate constants remain the same as in phase 1, but k_E^{in}=k_E^{out}=10.0 and V increases linearly with $\dot{V}$=1.0. Phase 3: V continues to increase with the same rate and k_3 increases with rate $\dot{k}_3$=1.0. The controller moves A towards the new set-point A_{set}^{theor}=$(k_4-k_E^{out})/k_6$=1.0 in both phase 2 and phase 3. In comparison to Fig. 11 no breakdown in phase 3 occurs due to a high initial M concentration.

The set-point (Eq. S14) is also defended for step-wise perturbations in V or k_3. For k_E^{in}=k_E^{out}=10.0 a change of k_3 from 2.0 to 8.0 (V kept constant at 25.0 and other rate constants remain as in Fig. S1) shows a numerical A_{ss} value of 1.005, while the same A_{ss} value is observed for a step-wise change of V from 25.0 to 100.0, while k_3 is kept constant at 2.0.

Transporter-based compensatory flux with exponentially increasing values of $\dot{V}$ and $\dot{k}_3$ and non-negligible k_E^{in} and k_E^{out} terms (k_E^{in}=k_E^{out}=10.0)

The autocatalytic rate of V is described by the equation

$$\dot{V} = \kappa V \tag{S15}$$

where κ is a constant (=0.1) and related to the doubling time of V by $\ln 2/\kappa$. Similarly, the autocatalytic increase of k_3 is described by

$$\dot{k}_3 = \zeta k_3 \tag{S16}$$

where $\ln 2/\zeta$ is the doubling time of k_3. In accordance with Eq. 48, ζ=0.2.

The rate equations for A and E take the form (assuming again that sufficient M is always present and $M/(k_5+M)$=1.0):

$$\dot{A} = \frac{k_2 E}{V} - k_3 \cdot A - \kappa \cdot A \tag{S17}$$

$$\dot{E} = k_4 \cdot E - k_6 \cdot A \cdot E + k_E^{in} - k_E^{out} \cdot E - \kappa \cdot E \tag{S18}$$

Setting $\dot{A}$=0 and calculating $\ddot{A}$=0 gives

$$\ddot{A} = \frac{k_2 \dot{E}}{V} - \frac{k_2 \dot{V}}{V^2} - \dot{k}_3 A_{ss} - \underbrace{\kappa \dot{A}_{ss}}_{=0} = 0 \tag{S19}$$

Inserting Eq. S18 into Eq. S19, and substituting the relationship between growing E, V, k_3 and the steady state in A (A_{ss} from Eq. S17),

$$E = \frac{V}{k_2}(k_3 + \kappa) A_{ss} \ , \tag{S20}$$

into the equation of $\ddot{A}$, we get:

$$\ddot{A} = k_4(k_3+\kappa)A_{ss} - k_6(k_3+\kappa)A_{ss}^2 + \frac{k_2 k_9}{V} - k_E^{out}(k_3+\kappa)A_{ss} - \kappa(k_3+\kappa)A_{ss} - \zeta k_3 A_{ss} - \frac{k_2 \kappa}{V} = 0 \tag{S21}$$

Rearranging leads to a quadratic equation in A_{ss}

$$A_{ss}^2 - \left(\frac{k_4}{k_6} - \frac{k_E^{out}}{k_6} - \frac{\kappa}{k_6} - \underbrace{\frac{\zeta k_3}{k_6(k_3+\kappa)}}_{\to \zeta/k_6} \right) A_{ss} - \underbrace{\frac{k_2}{V}(k_9 - \kappa)}_{\to 0} = 0 \tag{S22}$$

For increasing k_3 the term $\zeta k_3/(k_6(k_3+\kappa))$ approaches ζ/k_6, while for increasing large V the last term in Eq. S22 vanishes.

When both V and k_3 increase exponentially the set-point becomes

$$A_{ss} \approx \frac{k_4 - k_E^{out} - \kappa - \zeta}{k_6} \qquad \text{(S23)}$$

In comparison with linear growth (Eq. S14) exponential growth gives additional offsets in the set-point, which depend on κ and ζ, i.e., on how fast the exponential growth in V or k_3 occurs. This is shown in Fig. S2 where the perturbation profile of Fig. 14 and the rate constant values from Fig. 11 (Fig. 15c) are applied, except that k_E^{in} and k_E^{out} are changed in phases 2 and 3 from 1×10^{-5} to 10.0.

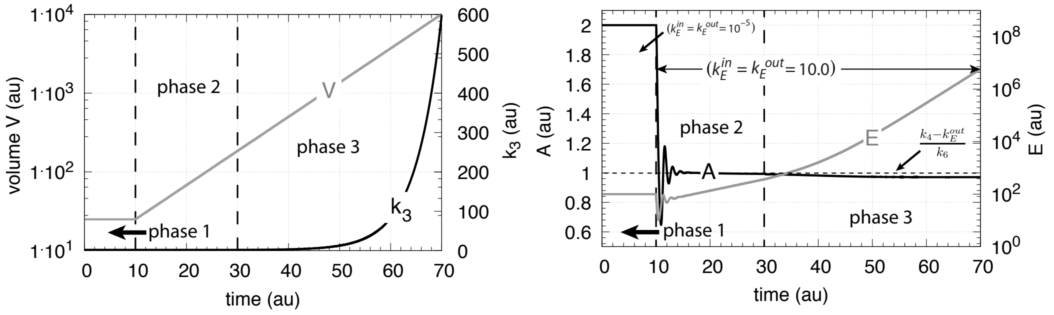

Figure S2. Performance of the motif 1 autocatalytic controller (Eqs. 33-35) with transporter based compensatory flux applying the perturbation profile from Fig. 14. Phase 1: constant volume V and constant k_3. Initial concentrations and rate constant values (at steady state) as in Fig. 11: V_0=25.0, $\dot{V}$=0.0, A_0=2.0, E_0=100.0, $M_0=1\times10^{12}$, k_2=1.0, k_3=2.0, $\dot{k}_3$=0.0, k_4=20.0, $k_5=1\times10^{-6}$, k_6=10.0, and $k_E^{in}=k_E^{out}=1\times10^{-5}$. The controller keeps A at its set-point at $A_{set}^{theor}=k_4/k_6$=2.0. Phase 2: rate constants remain the same as in phase 1, but $k_E^{in}=k_E^{out}$ are both changed to 10.0 and V increases exponentially with $\dot{V}=\kappa V$ ($\kappa=0.1$). Phase 3: V continues to increase exponentially and k_3 starts to increase exponentially with the rate law $\dot{k}_3=\zeta k_3$ ($\zeta=0.2$). The controller moves A towards a new steady state dependent on κ and/or ζ as outlined by Eq. S23. For comparison the set-point $k_4 - k_E^{out}/k_6$ (=1.0) for linear growth is indicated as a dashed line showing the offset from $k_4 - k_E^{out}/k_6$ due to exponential growth.

Cell-internal compensatory flux with constant values of $\dot{V}$ and $\dot{k}_3$ (k_E^{in}=k_E^{out}=1×10^{-5})

Assuming that N and M are sufficiently high to avoid controller breakdown by low N and M values, the rate equations for A and E are in this case (neglecting the $k_E^{in}-k_E^{out}\cdot E$ term with $k_E^{in} = k_E^{out} = 1\times10^{-5}$):

$$\dot{A} = k_2\cdot E - k_3\cdot A - A\left(\frac{\dot{V}}{V}\right) \tag{S24}$$

$$\dot{E} = k_4\cdot E - k_6\cdot A\cdot E - E\left(\frac{\dot{V}}{V}\right) \tag{S25}$$

Calculating $\ddot{A}$ and setting it to zero gives

$$\ddot{A} = k_2\dot{E} - \dot{k}_3 A + A\left(\frac{\dot{V}}{V}\right)^2 = 0 \tag{S26}$$

Inserting Eq. S25 into Eq S26

$$\ddot{A} = k_2\left[k_4 \underbrace{E}_{k_3A_{ss}/k_2} - k_6\cdot A\cdot \underbrace{E}_{k_3A_{ss}/k_2} - \underbrace{E}_{k_3A_{ss}/k_2}\left(\frac{\dot{V}}{V}\right)\right] - \dot{k}_3A_{ss} + A_{ss}\left(\frac{\dot{V}}{V}\right)^2 = 0 \tag{S27}$$

From the steady state condition of Eq. S24, we use approximately (for large V) $E = k_3A_{ss}/k_2$. Collecting terms in Eq. S27

$$k_3k_4A_{ss} - k_3k_6A_{ss}^2 - \dot{k}_3A_{ss} = 0 \tag{S28}$$

Dividing by A_{ss} gives

$$A_{ss} = \frac{k_4}{k_6} - \frac{\dot{k}_3}{k_3k_6} \tag{S29}$$

where k_4/k_6=A_{set}^{theor}.

Cell-internal compensatory flux with linearly increasing values of $\dot{V}$ and $\dot{k}_3$ and non-negligible k_E^{in} and k_E^{out} terms (k_E^{in}=k_E^{out}=10.0)

The rate equation for A is described by Eq. S24, while the rate equation for E now includes the $k_E^{in}-k_E^{out}\cdot E$ term:

$$\dot{E} = k_4\cdot E - k_6\cdot A\cdot E + k_E^{in} - k_E^{out}\cdot E - E\left(\frac{\dot{V}}{V}\right) \tag{S30}$$

Inserting Eq. S30 into the expression for $\ddot{A}$ (Eq. S26) gives:

$$\ddot{A} = k_2\left[k_4 E - k_6\cdot A\cdot E + k_E^{in} - k_E^{out}\cdot E - E\left(\frac{\dot{V}}{V}\right)\right] - \dot{k}_3 A_{ss} + A_{ss}\left(\frac{\dot{V}}{V}\right)^2 = 0 \tag{S31}$$

Setting $\dot{A}$=0 (Eq. S24), neglecting the $A\dot{V}/V$ term, we get the (approximate, for large V) relationship between increasing E and k_3 while A is kept at its steady state, i.e.,

$$E = \left(\frac{k_3}{k_2}\right) A_{ss} \tag{S32}$$

Inserting E from Eq. S32 into Eq. S31, neglecting the terms containing $\dot{V}/V$ gives

$$k_3 k_4 A_{ss} - k_3 k_6 A_{ss}^2 + k_2 k_E^{in} - k_3 k_E^{out} A_{ss} - \dot{k}_3 A_{ss} = 0 \tag{S33}$$

Rearranging Eq. S33 gives Eq. 61:

$$A_{ss}^2 - A_{ss}\left(\frac{k_4 - k_E^{out}}{k_6} - \frac{\dot{k}_3}{k_3 k_6}\right) - \frac{k_2 k_E^{in}}{k_3 k_6} = 0 \tag{S34}$$

Fig. S3 shows the behavior for linearly increasing V and k_3. When only V increases in phase 2 the controller moves A to the A_{ss} value described by the solution of Eq. S34. Although this steady state in A is dependent on the value of k_3, it is independent of how fast V grows. Finally, in phase 3 k_3 starts to grow and the controller moves A_{ss} to the set-point $(k_4 - k_E^{out})/k_6$ independent of the (linear) growth rate of k_3 (see, however, the chapter below when V and k_3 grow exponentially and k_E^{in}=k_E^{out}=10.0).

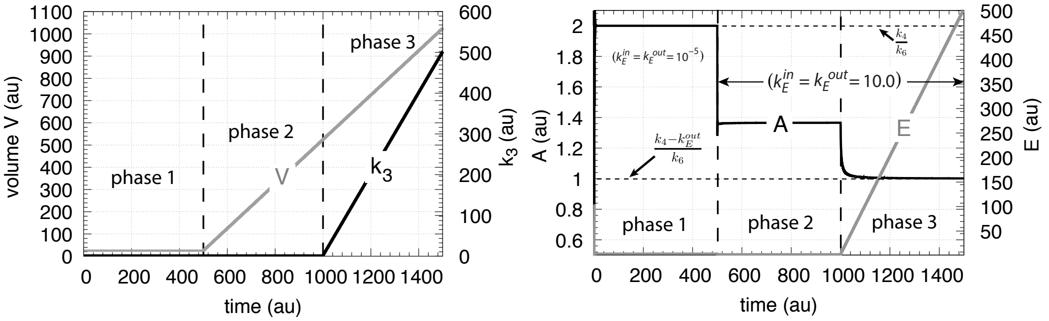

Figure S3. Performance of the motif 1 autocatalytic controller (Eq. 59 and Eqs. 34-35). Phase 1: constant volume V and constant k_3. Initial concentrations and rate constant values: V_0=25.0, $\dot{V}$=0.0, A_0=2.0, E_0=100.0, N_0=M_0=1×10^{12}, k_2=1.0, k_3=2.0, $\dot{k}_3$=0.0, k_4=20.0, $k_5=1 \times 10^{-6}$, k_6=10.0, and k_E^{in}=k_E^{out}=$1{\times}10^{-5}$. The controller keeps A at its set-point at A_{set}^{theor}=k_4/k_6=2.0. Phase 2: rate constants remain the same as in phase 1, but k_E^{in}=k_E^{out}=10.0 and V increases linearly with $\dot{V}$=1.0. The numerical value of A_{ss} is 1.3656 and independent of how fast V grows. This value is in excellent agreement with the solution of the quadratic equation (1.3660). Phase 3: V continues to increase with the same rate and k_3 starts to grow with rate $\dot{k}_3$=1.0. The controller moves A now towards the new set-point $(k_4{-}k_E^{out})/k_6$=1.0.

Cell-internal compensatory flux with exponential increase of $\dot{V}$ and $\dot{k}_3$ (k_E^{in}=k_E^{out}=$1{\times}10^{-5}$)

We have the same rate equations as above (Eqs. S24-S25) with k_E^{in}=k_E^{out}=$1{\times}10^{-5}$ (which we neglect in the analytical approach here). Since V and k_3 both grow exponentially we can write

$$\dot{V} = \kappa V \quad \& \quad \dot{k}_3 = \zeta k_3 \tag{S35}$$

where κ and ζ are constants. The rate equations are (assuming sufficient amounts of N and M, and, for the sake of simplicity, we set $N/(k_7 + N)$=$M/(k_5 + M)$=1.0)

$$\dot{A} = k_2{\cdot}E - k_3{\cdot}A - \kappa A \tag{S36}$$

$$\dot{E} = k_4 \cdot E - k_6 \cdot A \cdot E - \kappa E \tag{S37}$$

Assuming steady state in A ($\dot{A}$=0) we can write from Eq. S36

$$E = \left(\frac{k_3 + \kappa}{k_2} \right) A_{ss} \tag{S38}$$

where E increases in relationship with k_3 in order to keep A at its steady state.

Calculating $\ddot{A}$ and noting that $\dot{A}$=0 and that ζ and κ are constants, gives

$$\ddot{A} = k_2 \dot{E} - \dot{k}_3 A_{ss} = k_2 \left[k_4 E - k_6 \cdot A_{ss} \cdot E - E \cdot \kappa \right] - \dot{k}_3 A_{ss} = 0 \tag{S39}$$

Inserting the expression for E_{ss} from Eq. S38 into Eq. S39 and collecting terms gives

$$A_{ss} = \frac{k_4}{k_6} - \frac{\kappa}{k_6} - \frac{\dot{k}_3}{k_6 (k_3 + \kappa)} \tag{S40}$$

In Eq. S40 k_4/k_6 is the theoretical set-point A_{set}^{theor} the controller defends when step-wise perturbations are applied. The term κ/k_6 is the offset from A_{set}^{theor} due to the exponential increase of V, while the term $\dot{k}_3/(k_6(k_3+\kappa))$ is the offset due to the exponential increase of k_3. This last term can be reduced to the ratio ζ/k_6 by using $\dot{k}_3$=ζk_3 and observing that

$$\lim_{k_3 \to \infty} \frac{\dot{k}_3}{k_6 (k_3 + \kappa)} = \lim_{k_3 \to \infty} \frac{\zeta k_3}{k_6 (k_3 + \kappa)} \overset{\text{L'Hôpital}}{=} \frac{\zeta}{k_6} \tag{S41}$$

Referring to Fig. 25c, the numerical steady state is calculated at the end of phase 2 to be 1.99. The same offset of 0.01 is obtained for κ/k_6 from Eq. S40 (rate constant values are found in Fig. 22). At the end of phase 3 in Fig. 25c the numerical A_{ss} value is 1.971, while the overall calculated offset from Eq. S40 is 1.97 which includes the exponential increase of k_3 (Eq. 48) with a ζ of 0.2.

Cell-internal compensatory flux with exponentially increasing values of $\dot{V}$ and $\dot{k_3}$ and non-negligible k_E^{in} and k_E^{out} terms (k_E^{in}=k_E^{out}=10.0)

The rate equations and conditions are the same as in the previous section, except that $\dot{E}$ now includes the term $k_E^{in}-k_E^{out}{\cdot}E$, i.e.,

$$\dot{A} = k_2{\cdot}E - k_3{\cdot}A - \kappa A \tag{S36}$$

$$\dot{E} = k_4{\cdot}E - k_6{\cdot}A{\cdot}E + k_E^{in} - k_E^{out}{\cdot}E - \kappa E \tag{S42}$$

We calculate $\ddot{A}$, set $\dot{A}$ and $\ddot{A}$ to zero, and then insert Eq. S37 into the $\ddot{A}$-expression. Then all E's are substituted with the expression from Eq. S38, which gives the equation for $\ddot{A}$ and A_{ss}:

$$\begin{aligned}\ddot{A} =& k_2\dot{E} - \dot{k_3}A_{ss} = k_2\left[k_4E - k_6{\cdot}A_{ss}{\cdot}E + k_E^{in} - k_E^{out}{\cdot}E - E{\cdot}\kappa\right] - \dot{k_3}A_{ss}\\ &= k_2\left[k_4\left(\frac{k_3+\kappa}{k_2}\right)A_{ss}-k_6{\cdot}A_{ss}{\cdot}\left(\frac{k_3+\kappa}{k_2}\right)A_{ss}+k_E^{in}-k_E^{out}{\cdot}\left(\frac{k_3+\kappa}{k_2}\right)A_{ss}-\kappa\left(\frac{k_3+\kappa}{k_2}\right)A_{ss}\right]\\ &- \dot{k_3}A_{ss}\\ &= k_4(k_3+\kappa)A_{ss}-k_6(k_3+\kappa)A_{ss}^2+k_2k_E^{in}-k_E^{out}(k_3+\kappa)A_{ss}-\kappa(k_3+\kappa)A_{ss}-\dot{k_3}A_{ss}\\ &= -k_6(k_3+\kappa)A_{ss}^2+A_{ss}\left[k_4(k_3+\kappa) - k_E^{out}(k_3+\kappa)-\kappa(k_3+\kappa)-\dot{k_3}\right]+k_2k_E^{in}\\ &= 0\end{aligned} \tag{S43}$$

Dividing the last expression in Eq. S43 by $-k_6(k_3+\kappa)$ gives

$$A_{ss}^2-A_{ss}\left[\frac{k_4}{k_6}-\frac{k_E^{out}}{k_6}-\frac{\kappa}{k_6}-\frac{\zeta k_3}{k_6(k_3+\kappa)}\right]-\frac{k_2k_E^{in}}{k_6(k_3+\kappa)}=0 \tag{S44}$$

where $\dot{k_3}$ has been substituted by ζk_3 (see Eq. S35). When k_3 becomes large the term $k_3/(k_3+\kappa)$ goes to 1 and $k_2k_E^{in}/k_6(k_3+\kappa)$ goes to 0, such that Eq. S44 can be written as:

$$A_{ss}^2-A_{ss}\left[\frac{k_4}{k_6}-\frac{k_E^{out}}{k_6}-\frac{\kappa}{k_6}-\frac{\zeta}{k_6}\right]=0 \tag{S45}$$

and A_{ss} becomes:

$$A_{ss} = \frac{k_4}{k_6}-\frac{\kappa}{k_6}-\frac{\zeta}{k_6}-\frac{k_E^{out}}{k_6} \tag{S46}$$
