# Supplementary material for: Homeostatic controllers compensating for growth and perturbations
Source: PLoS One. 2019 Aug 12;14(8):e0207831. doi: 10.1371/journal.pone.0207831 (PMC6690524; doi:10.1371/journal.pone.0207831)
Supplement: S6 Text — (PDF) [file pone.0207831.s007.pdf]

Supporting Material, File S6 Text

Homeostatic Controllers Compensating for Growth and Perturbations

P. Ruoff[1*], O. Agafonov[1], D. M. Tveit[2], K. Thorsen[2], T. Drengstig[2]
[1]Centre for Organelle Research
[2]Department of Electrical Engineering and Computer Science,
University of Stavanger, Stavanger, Norway

*Corresponding author. Address: Centre for Organelle Research, University of Stavanger, N-4036 Stavanger, Norway, Tel.: (47) 5183-1887, Fax: (47) 5183-1750, E-mail: peter.ruoff@uis.no

Steady states and theoretical set-point for motif 2 zero-order controller

Transporter-based compensatory flux with constant values of $\dot{V}$ and $\dot{k}_3$

We refer to the rate equations for A and E (Eqs. 43-44), which are written in the following form:

$$\dot{A} = \frac{k_2 k_4}{k_4 + E} \cdot \frac{1}{V} - k_3 \cdot A - A\left(\frac{\dot{V}}{V}\right) \tag{S1}$$

$$\dot{E} = k_8 \cdot A - k_9 - E\left(\frac{\dot{V}}{V}\right) \tag{S2}$$

by setting in Eq. 44 $M/(k_{11}+M)=E/(k_{10}+E)=1$.

Calculating $\ddot{A}$ gives

$$\ddot{A} = -\frac{k_2 k_4}{(k_4+E)^2} \cdot \frac{\dot{E}}{V} - \left(\frac{k_2 k_4}{k_4+E}\right)\frac{\dot{V}}{V^2} - \dot{k}_3 A + A\left(\frac{\dot{V}}{V}\right)^2 \tag{S3}$$

Inserting Eq. S2 into Eq. S3 leads to

$$\ddot{A} = -\frac{k_2 k_4}{(k_4+E)^2} \cdot \frac{1}{V}\left[k_8 \cdot A - k_9 - E\left(\frac{\dot{V}}{V}\right)\right] - \left(\frac{k_2 k_4}{k_4+E}\right)\frac{\dot{V}}{V^2} - \dot{k}_3 A + A\left(\frac{\dot{V}}{V}\right)^2 \tag{S4}$$

Multiplying Eq. S4 by $V(k_4+E)^2/(k_2 k_4)$ gives

$$\ddot{A} = -k_8 A + k_9 + E\left(\frac{\dot{V}}{V}\right) - (k_4+E)\left(\frac{\dot{V}}{V}\right) - \dot{k}_3 A \cdot \frac{V(k_4+E)^2}{k_2 k_4} + A\left(\frac{\dot{V}}{V}\right)^2 \cdot \frac{V(k_4+E)^2}{k_2 k_4} \tag{S5}$$

Setting the $\dot{V}/V$ terms in Eq. S5 to zero we get

$$\ddot{A} = -k_8 A + k_9 - \dot{k_3} A \cdot \frac{V(k_4 + E)^2}{k_2 k_4} \tag{S6}$$

Setting Eq. S1 to zero and neglecting the $\dot{V}/V$ terms gives the relationship between decreasing E and increasing V and k_3 to keep A at a constant steady state A_{ss}, i.e.

$$\frac{k_2 k_4}{(k_4 + E) \cdot V} = k_3 A_{ss} \quad \Rightarrow \quad (k_4 + E)^2 = \frac{(k_2 k_4)^2}{k_3^2 A_{ss}^2 V^2} \tag{S7}$$

Inserting $(k_2 k_4)^2$ from Eq. S7 into Eq. S6 and setting $\ddot{A}$=0 gives

$$A_{ss} = \underbrace{\frac{k_9}{k_8}}_{A_{set}^{theor}} - \underbrace{\frac{\dot{k_3} k_2 k_4}{k_3^2 A_{ss} V}}_{\text{offset}} \tag{S8}$$

For constant $\dot{k_3}$ and increasing values of V and k_3 the offset term $\dot{k_3} k_2 k_4 / k_3^2 A_{ss} V$ goes to zero and A_{ss} is kept by the controller at its theoretical set-point A_{set}^{theor}=k_9/k_8 as clearly seen in Fig. 13. Since a constant A_{ss} level by this controller type is maintained by decreasing E values the negative feedback loop will break when E becomes low and the controller reaches its capacity limits (Eq. 48).

Cell-internal compensatory flux with constant values of $\dot{V}$ and and $\dot{k_3}$

In this case the rate equations (Eqs. 62-63) are written as

$$\dot{A} = \frac{k_4 k_6}{k_4 + E} - k_3 \cdot A - A \left(\frac{\dot{V}}{V} \right) \tag{S9}$$

$$\dot{E} = k_8 \cdot A - k_9 - E \left(\frac{\dot{V}}{V} \right) \tag{S10}$$

by setting $N/(k_7{+}N){=}E/(k_{10}{+}E){=}M/(k_{11}{+}M){=}1$. Taking the second-time derivative $\ddot{A}$ gives

$$\ddot{A} = -\frac{k_4 k_6}{(k_4{+}E)^2}\dot{E} - \dot{k}_3 A + A\left(\frac{\dot{V}}{V}\right)^2 \tag{S11}$$

Inserting $\dot{E}$ from Eq. S10 into Eq. S11

$$\ddot{A} = -\frac{k_4 k_6}{(k_4{+}E)^2}\left[k_8{\cdot}A - k_9 - E\left(\frac{\dot{V}}{V}\right)\right] - \dot{k}_3 A + A\left(\frac{\dot{V}}{V}\right)^2 \tag{S12}$$

Setting Eq. S9 to zero and neglecting the $\dot{V}/V$ term, we have the condition how E has to decrease for increasing k_3 to keep A constant at A_{ss}, i.e.,

$$\frac{k_4 k_6}{(k_4{+}E)} = k_3 A_{ss} \quad \Rightarrow \quad (k_4{+}E)^2 = \frac{(k_4 k_6)^2}{k_3^2 A_{ss}^2} \tag{S13}$$

Substituting $(k_4{+}E)^2$ in Eq. S11 by $(k_4 k_6)^2/k_3^2 A_{ss}^2$, setting the resulting equation to zero, and neglecting the $\dot{V}/V$ terms, leads to

$$A_{ss} = \frac{k_9}{k_8} - \frac{\dot{k}_3 k_4 k_6}{k_3^2 k_8 A_{ss}} \tag{S14}$$

where $A_{set}^{theor}{=}k_9/k_8$ and the offset term is zero for $\dot{k}_3{=}0$, and goes to zero when $\dot{k}_3$ is constant and k_3 increases.

Cell-internal compensatory flux with exponential increase of $\dot{V}$ and $\dot{k}_3$

i) Exponential increase in V and constant k_3 (phase 2). We start again with the rate equations

$$\dot{A} = \frac{k_4 k_6}{k_4{+}E} - k_3{\cdot}A - A\left(\frac{\dot{V}}{V}\right) \tag{S9}$$

$$\dot{E} = k_8 \cdot A - k_9 - E\left(\frac{\dot{V}}{V}\right) \tag{S2}$$

In the case $\dot{k_3}$=0, but V increases exponentially, say $\dot{V}$=κV, A_{ss} and E_{ss} show constant value, where A_{ss} shows an offset above A_{set}^{theor} (overcompensation). The steady state in A can be calculated by setting Eq. S2 to zero, i.e.,

$$A_{ss} = \underbrace{\frac{k_9}{k_8}}_{A_{set}^{theor}} + \underbrace{\frac{\kappa}{k_8} E_{ss}}_{\text{overcompensated offset}} \tag{S15}$$

ii) Exponential increase in V and k_3 (phase 3). Assuming that $\dot{V}$=κV and $\dot{k_3}$=ζk_3 with κ and ζ constants, we can calculate $\ddot{A}$

$$\ddot{A} = -\frac{k_4 k_6}{(k_4{+}E)^2}\dot{E} - \dot{k_3} A \tag{S16}$$

assuming that $\dot{A}$=0. Inserting Eq. S2 (note that $\dot{V}/V$=κ) into Eq. S16 and setting Eq. S16 to zero gives the expression for the steady state of A, A_{ss},

$$-\frac{k_4 k_6}{(k_4{+}E)^2}\left[k_8 \cdot A_{ss} - k_9 - \kappa \cdot E\right] - \dot{k_3} A_{ss} = 0 \tag{S17}$$

leading to

$$A_{ss} = -\frac{k_4 k_6}{\dot{k_3}(k_4{+}E)^2}\left[k_8 \cdot A_{ss} - k_9 - \kappa \cdot E\right] \tag{S18}$$

Note, that while A is in a steady state, E is decreasing (derepressing) in order to increase the compensatory flux. Eq. S18 can be rewritten as

$$A_{ss} = -\underbrace{\frac{k_4 k_6 k_8}{\dot{k}_3 (k_4 + E)^2}}_{\gamma_0} \left[A_{ss} - \overbrace{\underbrace{\frac{k_9}{k_8}}_{A_{set}^{theor}} - \underbrace{\frac{\kappa}{k_8}}_{\text{overcompensated offset}} E}^{A_{set}^{app}} \right] \tag{S19}$$

where $A_{set}^{apparent}$ is an "apparent set-point". Thus, Eq. S19 can be written as

$$A_{ss} = -\gamma_0 (A_{ss} - A_{set}^{app}) \quad \Rightarrow \quad A_{ss} = \left(\frac{\gamma_0}{1 + \gamma_0} \right) A_{set}^{app} \tag{S20}$$

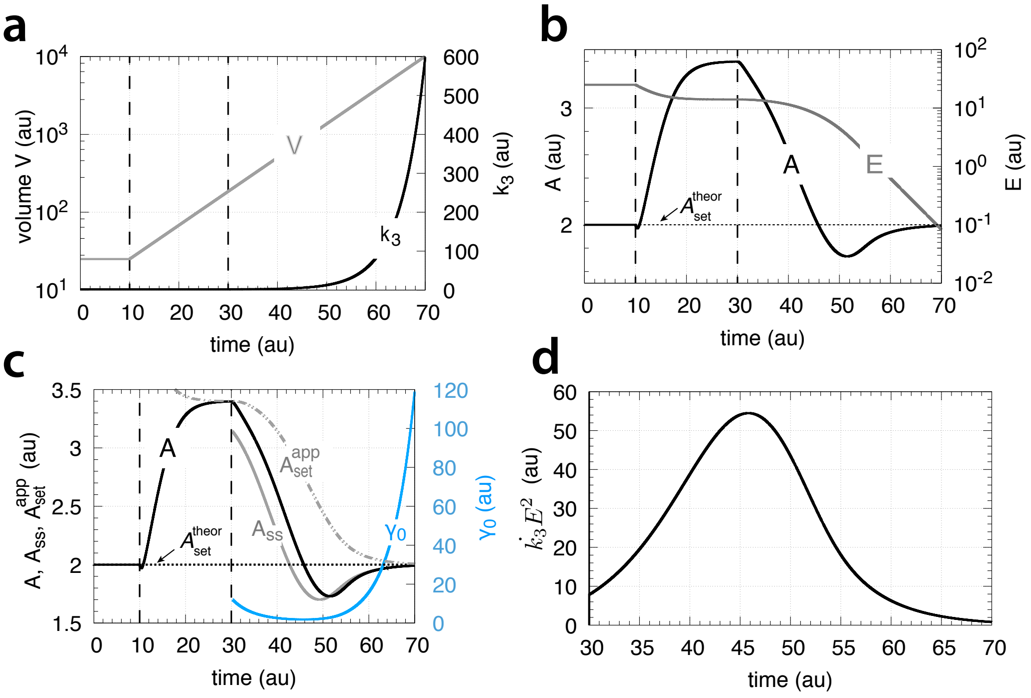

Figure S1. (a) Perturbation profile of V and k_3 (same figure as Fig. 14). (b) Response of controller (same results as in Fig. 25d). (c) Behaviors of A_{ss} and A_{set}^{app} as a function of time (Eq. S20). (d) By the end of phase 3 E^2 decreases more rapidly than the exponential increase of $\dot{k}_3$, which is indicated by the product $\dot{k}_3 E^2$ going to zero.

Fig. S1c shows how γ_0 and A_{set}^{app} changes with respect to the controller's behavior when exposed to exponential increase in V and k_3 with the response shown in Fig. 25d. For convenience the perturbation profile and the controller's response are repeated in Figs. S1a and b. The derepression by decreasing E leads to an increase in γ_0 (Fig. S1c, curve outlined in blue). The increase in γ_0 is the result of E^2 decreasing more rapidly than the exponential increase of $\dot{k}_3$. This is indicated in Fig. S1 where the product $\dot{k}_3 E^2$ during phase 3 decreases and $A_{ss} \rightarrow A_{set}^{theor}$.
