# Supplementary material for: Homeostatic controllers compensating for growth and perturbations
Source: PLoS One. 2019 Aug 12;14(8):e0207831. doi: 10.1371/journal.pone.0207831 (PMC6690524; doi:10.1371/journal.pone.0207831)
Supplement: S7 Text — (PDF) [file pone.0207831.s008.pdf]

# Supporting Material, File S7 Text

# Homeostatic Controllers Compensating for Growth and Perturbations

P. Ruoff[1*], O. Agafonov[1], D. M. Tveit[2], K. Thorsen[2], T. Drengstig[2]
[1]Centre for Organelle Research
[2]Department of Electrical Engineering and Computer Science,
University of Stavanger, Stavanger, Norway

*Corresponding author. Address: Centre for Organelle Research, University of Stavanger, N-4036 Stavanger, Norway, Tel.: (47) 5183-1887, Fax: (47) 5183-1750, E-mail: peter.ruoff@uis.no

## Novel antithetic integral controller arrangements and steady states in a motif 2 background

### Novel antithetic integral controller arrangements

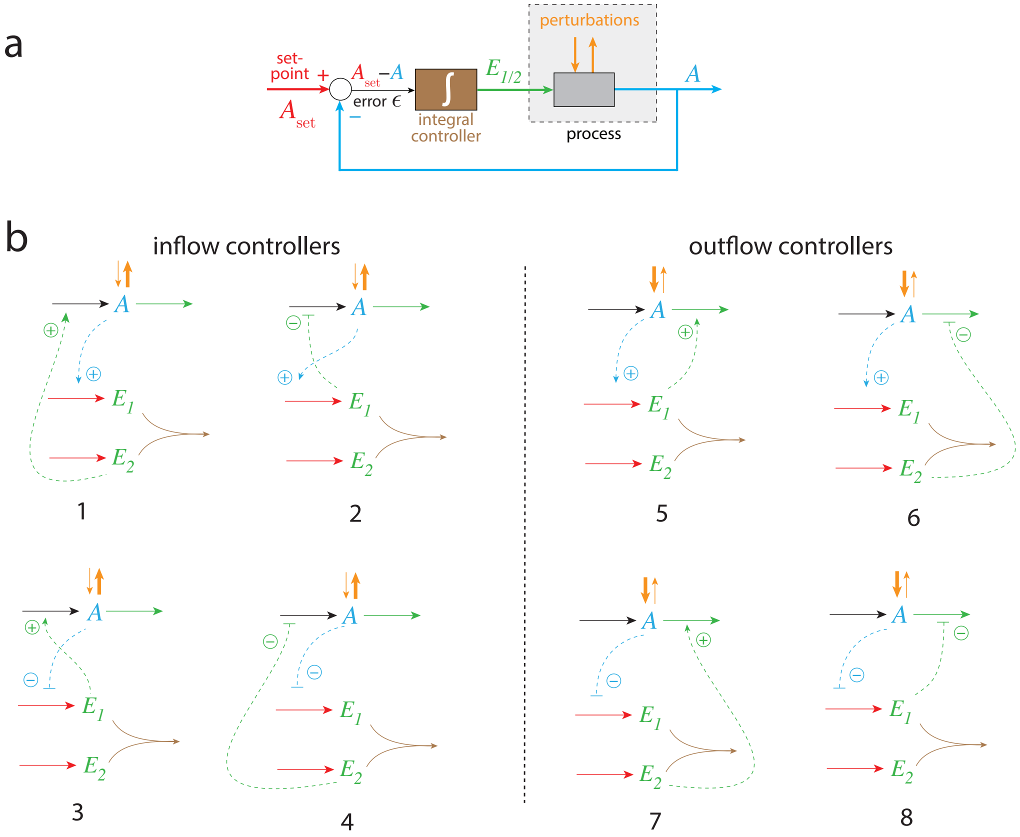


**Figure S1.** (a) Color-coded representation of a negative feedback loop containing an integral controller. (b) Molecular feedback schemes where the antithetic integral controller (outlined in brown) is embedded within different structural feedback loops which relate to the basic feedback motifs described in Ref. (1). Colors indicate how the different kinetic and signaling processes relate to the parts in the feedback loop in (a). Numbers define the different motifs.

Fig. S1a shows a generalized feedback loop containing an integral controller outlined in brown color. In Fig. S1b eight basic molecular feedback arrangements are shown in corresponding color code. The antithetic integral controller (also outlined in brown) is due to the removal of $E_1$ and $E_2$ by second-order kinetics. Activating and inhibitory signaling is shown by the dashed lines. In the following we describe some of the steady state behaviors in relation to the motif 2 antithetic controller.

**Steady states and theoretical set-point for motif 2 antithetic controller**

**Transporter-based compensatory flux with constant values of $\dot{V}$ and $\dot{k}_3$**

The rate equations (Eqs. 76, 22, and 23) are

$$\dot{A} = \frac{k_2 \cdot k_{10}}{k_{10} + E_1} \cdot \frac{1}{V} - k_3 \cdot A - A\left(\frac{\dot{V}}{V}\right) \tag{S1}$$

$$\dot{E}_1 = A\left(\frac{k_4 \cdot M}{k_5 + M}\right) - k_6 \cdot E_1 \cdot E_2 - E_1\left(\frac{\dot{V}}{V}\right) \tag{S2}$$

$$\dot{E}_2 = \frac{k_8 \cdot O}{k_9 + O} - k_6 \cdot E_1 \cdot E_2 - E_2\left(\frac{\dot{V}}{V}\right) \tag{S3}$$

In deriving an expression for the steady state in $A$, we assume that precursors $M$ and $O$ are in sufficient amounts such that

$$\frac{M}{k_5 + M} = \frac{O}{k_9 + O} = 1$$

We can get an expression for $A$ directly when inspecting Eq. S2. Rewriting Eq. S2 gives

$$k_4 \cdot A = \dot{E}_1 + k_6{\cdot}E_1{\cdot}E_2 + E_1\left(\frac{\dot{V}}{V}\right) \tag{S4}$$

During the first phase in Fig. 29a, when $\dot{V}=\dot{k_3}=0$, and $A$, $E_1$, and $E_2$ are in a steady state, we get

$$A_{ss} = \frac{k_6}{k_4}E_1{\cdot}E_2 = \frac{k_8}{k_4} = A_{set}^{theor} \tag{S5}$$

showing that $E_1{\cdot}E_2$ is constant. In case $V$ and $k_3$ are linearly increasing the $E_1\dot{V}/V$ term in Eq. S4 can be neglected, as $E_1$ is getting low and the $\dot{V}/V$ term goes to zero. $\dot{E}_1$ is negative, but low. Ignoring $\dot{E}_1$ shows that $A$ can be described in term of $E_1E_2$

$$A = \frac{k_6}{k_4}E_1{\cdot}E_2 \tag{S6}$$

In fact, $A$ and $k_6E_1E_2/k_4$ follow each other very closely (Fig. S2), even when the controller is no longer able to cope with the linear increase of $V$ and $k_3$

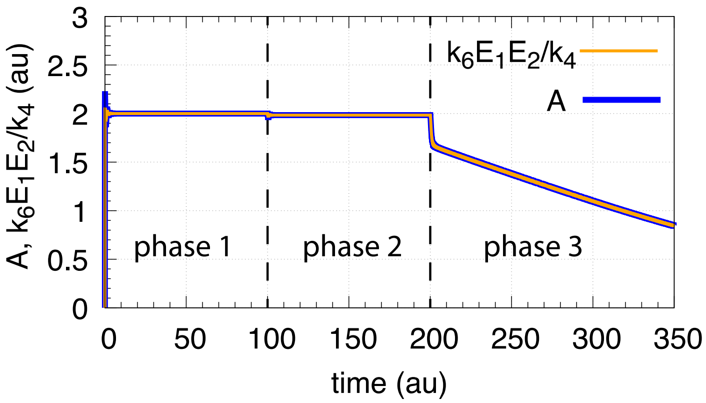


**Figure S2.** Same system as in Fig. 29a with $k_6E_1E_2/k_4$ (orange curve) overlayed on $A$ (blue curve), showing that $k_6E_1E_2/k_4$ describes the behavior of $A$ closely.

Fig. S2 shows that in phase 3 the product $E_1E_2$ can no longer be kept constant. Subtracting Eq. S2 from Eq. S3 and solving for $A$ gives

$$A = \underbrace{\frac{k_8}{k_4}}_{A_{set}^{theor}} - \frac{\mathrm{d}(E_2 - E_1)}{\mathrm{d}t} - (E_2 - E_1)\left(\frac{\dot{V}}{V}\right) \tag{S7}$$

indicating that the increase in $E_2$ (Fig. 29a) cannot be matched by the derepressing/decreasing $E_1$, resulting in the decrease of $A$ and the breakdown of the controller.

### Transporter-based compensatory flux with exponential increase of $\dot{V}$ and $\dot{k}_3$

In case $V$ and $k_3$ increase exponentially $k_6E_1E_2/k_4$ still describes the behavior of $A$, but the controller is not able (in comparison with a linear increase in $V$ and $k_3$) to keep $E_1E_2$ constant (see Fig. S3).

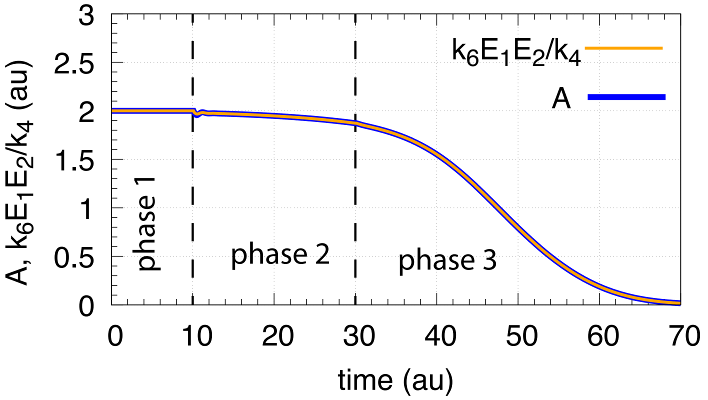


**Figure S3.** Same system as in Fig. 29b with $k_6E_1E_2/k_4$ (orange curve) overlayed on $A$ (blue curve).

The increasing $E_2$ values cannot be balanced by the decreasing $E_1$ concentration leading to controller breakdown.

**Cell-internal compensatory flux with constant values of $\dot{V}$ and and $\dot{k}_3$**

When the compensatory flux is cell internal the rate equation for $A$ (Eq. 76 with $N/(k_7{+}N) = 1$) becomes

$$\dot{A} = \frac{k_2{\cdot}k_{10}}{k_{10}{+}E_1} - k_3 \cdot A - A\left(\frac{\dot{V}}{V}\right) \tag{S8}$$

while the rate equations for $E_1$ and $E_2$ remain as described by Eqs. S2 and S3. Calculating $\ddot{A}$ and setting it to zero leads to the following expression

$$\ddot{A} = -\frac{k_2{\cdot}k_{10}}{(k_{10}{+}E_1)^2}\dot{E}_1 - \dot{k}_3{\cdot}A - k_3{\cdot}\dot{A} + A\left(\frac{\dot{V}}{V}\right)^2 = 0 \tag{S9}$$

Considering first the case that $k_3$ is kept constant ($\dot{k}_3$=0), while $V$ increases linearly, we observe (phase 2, Fig. 29c) that $A$ attains a stable steady state with $\dot{A}$=0. Considering further that $\dot{V}/V \to 0$, we arrive at the condition that $\dot{E}_1$=0, i.e.

$$\ddot{A} = -\frac{k_2{\cdot}k_{10}}{(k_{10}{+}E_1)^2}\dot{E}_1 = 0 \tag{S10}$$

Since $\dot{A} = \dot{E}_1 = \dot{V}/V = 0$, we arrive at Eq. S5 showing that in case of a cell-internal compensatory flux the m2-antithetic controller is able to keep $A$ at $A_{set}^{theor}$ as long as there is sufficient supply for $A$, $E_1$, and $E_2$ and that the maximum compensatory flux $k_2$ has not been reached.

When $k_3$ increases linearly, the term $\dot{k}_3{\cdot}A$ in Eq. S9 cannot be ignored. Inserting Eq. S2 into Eq. S9, observing from the numerical calculation that

$\dot{A}$=0, and approximating $\dot{V}/V = 0$, Eq. S9 is written as

$$\left[ k_4 A_{ss} - k_8 - \underbrace{E_1 \left( \frac{\dot{V}}{V} \right)}_{\to 0} \right] = -\frac{\dot{k}_3 (k_{10}+E_1)^2}{k_2 k_{10}} A_{ss} \tag{S11}$$

Dividing the left- and right-hand side of Eq. S11 by $k_4$, observing that $k_8/k_4 = A_{set}^{theor}$, and rearranging the equation, gives

$$A_{ss} \left( 1 + \frac{\dot{k}_3 (k_{10}+E_1)^2}{k_2 k_4 k_{10}} \right) = A_{set}^{theor} \tag{S12}$$

or

$$A_{ss} = \frac{A_{set}^{theor}}{1 + \frac{\dot{k}_3 (k_{10}+E_1)^2}{k_2 k_4 k_{10}}} \tag{S13}$$

Eq. S13 indicates that when both $V$ and $k_3$ increase linearly there should be an offset in $A_{ss}$ below $A_{set}^{theor}$, but the "offset term"

$$\frac{\dot{k}_3 (k_{10}+E_1)^2}{k_2 k_4 k_{10}}$$

is generally low, because $k_2$ represents the maximum compensatory flux, which in the calculations is $1 \times 10^5$. The "offset term" can further be reduced by increasing the aggressiveness of the controller when increasing $k_4$ and $k_8$ values, but maintaining the $k_8/k_4$ ratio, i.e., keeping $A_{set}^{theor}$ constant.

## Cell-internal compensatory flux with exponential increase of $\dot{V}$ and and $\dot{k}_3$

Fig. 29d shows that when only $V$ increases exponentially during phase 2 with $\dot{V} = \kappa V$ ($\kappa$ being a constant), and $k_3$ is kept constant ($\dot{k}_3 = 0$), the controller is able to maintain a constant steady state in $A$, as well as in $E_1$ and $E_2$. The rate equations in this case are

$$\dot{A} = \frac{k_2 \cdot k_{10}}{k_{10} + E_1} - k_3 \cdot A - \kappa A \tag{S14}$$

$$\dot{E}_1 = k_4 A - k_6 \cdot E_1 \cdot E_2 - \kappa E_1 \tag{S15}$$

$$\dot{E}_2 = k_8 - k_6 \cdot E_1 \cdot E_2 - \kappa E_2 \tag{S16}$$

Since $A$, $E_1$, and $E_2$ are during phase 2 in a steady state (Fig. 29d) we get an expression for $A_{ss}$, by setting Eq. S15 to zero and solving for $A_{ss}$

$$A_{ss} = \frac{k_6}{k_4} E_1 \cdot E_2 + \frac{\kappa}{k_4} E_1 \tag{S17}$$

Another expression for $A_{ss}$ can be found by calculating $\dot{E}_1 - \dot{E}_2$ and setting the resulting expression to zero ($E_1$ and $E_2$ are in a steady state)

$$\dot{E}_1 - \dot{E}_2 = k_4 \cdot A_{ss} - k_8 + \kappa (E_2 - E_1) = 0 \tag{S18}$$

which gives

$$A_{ss} = \underbrace{\frac{k_8}{k_4}}_{A_{set}^{theor}} + \underbrace{\frac{\kappa (E_1 - E_2)}{k_4}}_{\text{overcompensation part}} \tag{S19}$$

which identifies the factors leading to the overcompensation. Interestingly, increasing the controller aggressiveness by increasing $k_8$ and $k_4$, but keeping their ratio ($A_{set}^{theor}$) constant, the overcompensation can be reduced and $A_{ss}$ will approach $A_{set}^{theor}$.

Setting Eqs. S19 and S17 equal, and solving for $k_6 E_1 E_2 / k_4$ leads to the relationship

$$\frac{k_6}{k_4} E_1 \cdot E_2 = A_{set}^{theor} - \frac{\kappa E_2}{k_4} \tag{S20}$$

showing that $k_6 E_1 E_2 / k_4$ lies (under steady state conditions) slightly below $A_{set}^{theor}$.

When in phase 3 $k_3$ increases exponentially, $E_1$ decreases. The resulting derepression by $E_1$ moves $A_{ss}$ towards $A_{set}^{theor}$, but when the $E_1$ concentration becomes too low due to the increasing $E_2$ (which removes $E_1$) homeostasis is lost once $E_2$ exceeds $E_1$. For low $E_1$ values $\dot{E}_1$ is also low, and, as Eq. S15 indicates, the decreasing $A$ concentrations is quite well described by $k_6 E_1 E_2 / k_4$ (Fig. S4).

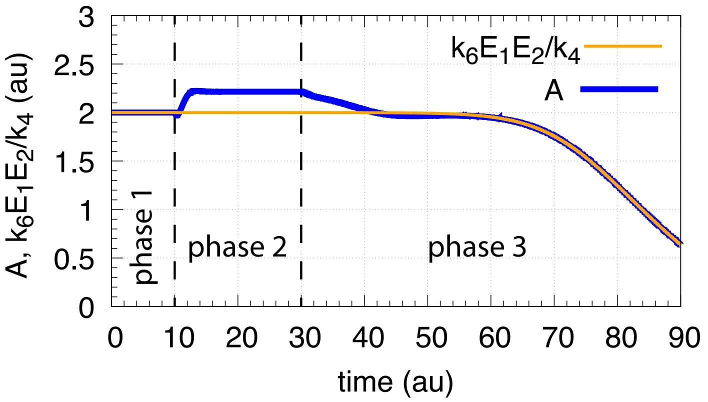


**Figure S4.** Same system as in Fig. 29d with $k_6 E_1 E_2 / k_4$ (orange curve) overlayed on $A$ (blue curve).

## References


[1] Drengstig, T.; Jolma, I.; Ni, X.; Thorsen, K.; Xu, X.; Ruoff, P. *Biophys J* **2012**, *103*(9), 2000–2010.
